# Unravelling Enzymatic Features in a Supramolecular Iridium Catalyst by Computational Calculations

**DOI:** 10.1002/chem.202201970

**Published:** 2022-08-12

**Authors:** Michele Tomasini, Lucia Caporaso, Jonathan Trouvé, Jordi Poater, Rafael Gramage‐Doria, Albert Poater

**Affiliations:** ^1^ Institut de Química Computacional i Catàlisi Departament de Química Universitat de Girona c/Mª Aurèlia Capmany 69 17003 Girona Catalonia Spain; ^2^ Department of Chemistry University of Salerno Via Ponte Don Melillo 84084 Fisciano Italy; ^3^ Univ Rennes CNRS ISCR – UMR 6226 F-35000 Rennes France; ^4^ Departament de Química Inorgànica i Orgànica & IQTCUB Universitat de Barcelona 08028 Barcelona Spain; ^5^ ICREA 08010 Barcelona Spain

**Keywords:** C−H functionalization, DFT calculations, enzyme mimics, iridium, supramolecular catalysis

## Abstract

Non‐biological catalysts following the governing principles of enzymes are attractive systems to disclose unprecedented reactivities. Most of those existing catalysts feature an adaptable molecular recognition site for substrate binding that are prone to undergo conformational selection pathways. Herein, we present a non‐biological catalyst that is able to bind substrates via the induced fit model according to in‐depth computational calculations. The system, which is constituted by an inflexible substrate‐recognition site derived from a zinc‐porphyrin in the second coordination sphere, features destabilization of ground states as well as stabilization of transition states for the relevant iridium‐catalyzed C−H bond borylation of pyridine. In addition, this catalyst appears to be most suited to tightly bind the transition state rather than the substrate. Besides these features, which are reminiscent of the action modes of enzymes, new elementary catalytic steps (i. e. C−B bond formation and catalyst regeneration) have been disclosed owing to the unique distortions encountered in the different intermediates and transition states.

## Graphical abstract

**Computational calculations** aiming at elucidating the reaction mechanism of a supramolecular iridium‐catalyzed C−H bond borylation enabled the identification of unique intermediates and transition states similar to those found in enzyme catalysis. The unexpected energies associated to the elementary steps are reached thanks to the relevant distortion effects at play, which are upgraded by the molecular‐recognition pocket from the catalyst.

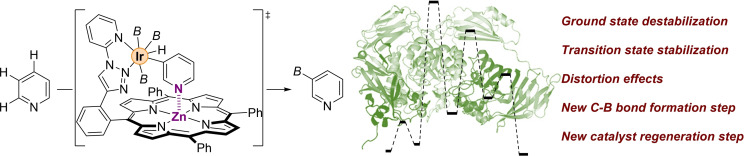

## Introduction

Chemical transformations catalyzed by enzymes, Nature's catalysts, exhibit highly high levels of activity and excellent levels of chemo‐, regio‐ and stereo‐selectivity.^[1]^ Although not fully understood, the main reasons for these superb performances appear to be the (1) physicochemical protection of the active site in a confined space within the enzyme,^[2]^ (2) the pre‐organization of the substrate around the active site by means of weak interactions occurring in the second coordination sphere,^[3]^ (3) the exploitation of cooperative reaction mechanisms (redox active, proton shuttles, etc.) including allosteric behavior by means of co‐factors,^[4]^ and (4) the subtle motions and distortions taking place near the active site to destabilize ground states and stabilize key transition states.^[5]^

Consequently, many of these strategies have been successfully implemented in abiological catalysts to improve state‐of‐the‐art catalysts.^[6]^ These so‐called supramolecular catalysts aim at mimicking such enzymatic properties in view to control the reaction outcome.^[7]^ For instance, catalysts confined in discrete (supra)molecular cages^[8]^ as well as those featuring substrate pre‐organization^[9]^ have been elegantly demonstrated to be powerful in achieving unique reactivities since the seminal studies by Breslow and Crabtree, independently.^[10]^

On the other hand, from the many supramolecular catalysts known to date, none of the studies involving them managed to identify the relevance of distortions occurring between the substrate and the catalyst. As currently known for biological enzymes, such distortions are known to play a central role in the activities and selectivities observed.^[5]^ The absence of these studies is likely based on the fact that most supramolecular catalysts are equipped with a hydrogen‐bonding or ion‐pairing substrate‐recognition site that are still flexible enough to accommodate substrates via conformational selection pathways.^[11]^ Although this translates into catalytic systems able to reach an atom‐precise reactivity in a predictive fashion,^[9]^ they fail to provide a model to identify and understand any distortion taking place between the catalyst and the substrate.^[12]^ Herein, we provide an in‐depth mechanistic study by purely computational means that highlights the importance of such distortions in a non‐biological catalyst appended with a rigid and unbendable substrate‐recognition site, aimed at destabilizing ground states and stabilizing transition states.^[13]^

**Figure 1:**
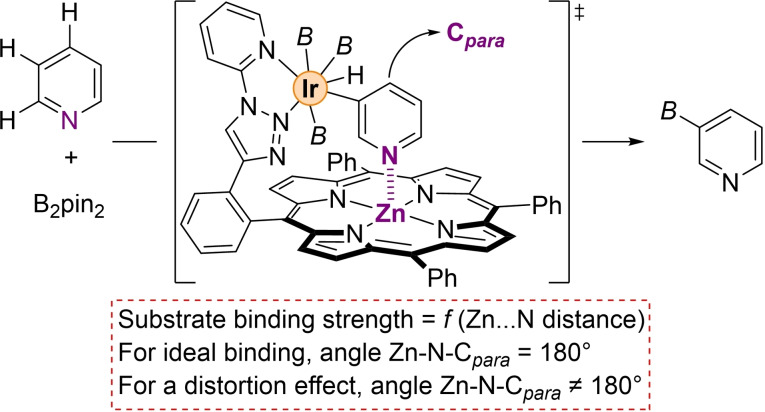
Supramolecular catalysis considered in this work in order to study how distortions effects are at play during the catalytic cycle in a similar manner as enzymes do for biological catalysis. *B*=(pinacolato)boron.

It has recently been shown by some of us that a supramolecular iridium catalyst equipped with a zinc‐porphyrin as a substrate‐recognition site led to *meta*‐selective C−H bond borylation of pyridines following the enzymatic Michaelis‐Menten rate equation law as enzymes do.^[14]^ The remote and kinetically labile Zn⋅⋅⋅N interaction enables the pyridine substrate to be located at a given distance from the catalytically active iridium site to reach the observed regio‐selectivity (Figure 1). This supramolecular catalyst features a zinc‐porphyrin moiety as a conformationally restricted, substrate‐recognition site since the zinc center can exclusively bind pyridines through the axial vacant site remaining almost unchanged before and after binding to pyridine substrates as shown by Sanders in organocatalysis.^[15]^ In addition, the catalytically active iridium intermediates should tightly activate the C−H bond from the pyridine substrate, thus imposing also a restricted conformation on the pyridine substrate while binding to the zinc center. As such, any changes deviating from the ideal linearity between the pyridine and the zinc binding can be followed as distortion effects between the substrate and the overall catalyst structure (Figure 1). We reasoned that this catalytic system could be an appropriate model to study catalyst‐substrate distortions at the molecular level by density functional theory (DFT) calculations. Moreover, owing to the rigidity of the porphyrin backbone, the distances between the zinc center in the substrate‐recognition site and the nitrogen atom from the substrate can directly be correlated to the strength of such non‐covalent interaction (Figure 1).

## Results and Discussion

For all the computed iridium complexes a singlet ground state has been considered throughout the reaction pathway, since test calculations on the triplet state lie at least 20 kcal mol^−1^ higher in energy (see Figure S1 in the Supporting Information).^[16]^ The Gibbs energy results were obtained at the B3LYP‐D3/Def2TZVP∼sdd(smd‐p‐xylene)//BP86‐D3/Def2SVP∼sdd level of theory.

**Scheme 1:**
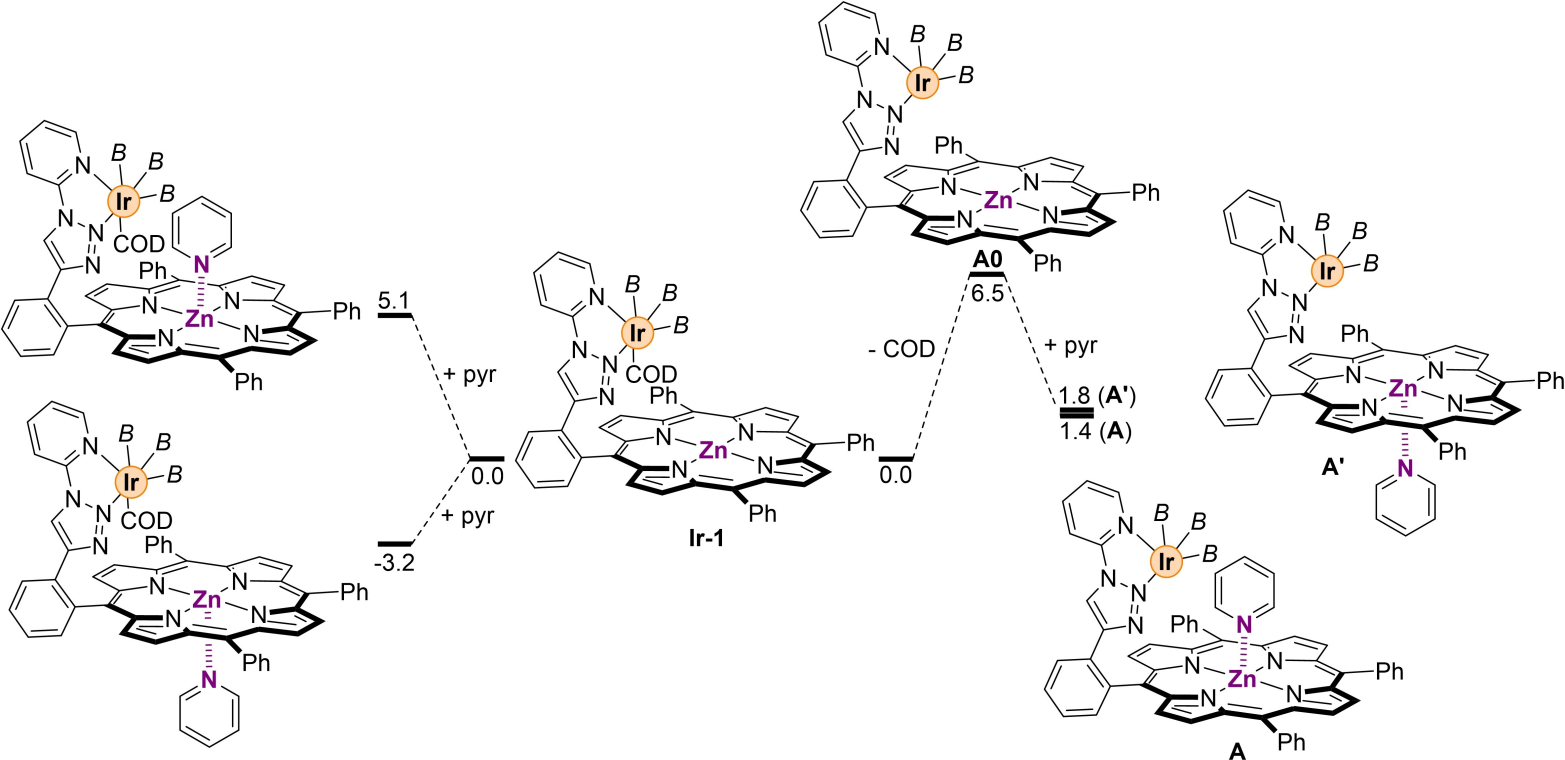
Probing pyridine substrate binding to the molecular recognition site of the supramolecular catalyst starting from the catalytically productive **Ir‐1** species. *B*=(pinacolato)boron, pyr=pyridine. Relative Gibbs free energies in kcal mol^−1^.

We initially focused on the formation of the active iridium(III) species and its compatibility with the binding of a pyridine substrate to the remote zinc‐porphyrin site (Scheme 1). The starting point of this study is the **Ir‐1** complex that experimentally forms upon reaction of the supramolecular ligand **L** with the [Ir(COD)Cl]_2_ (COD=1,5‐cyclooctadiene) precursor under borylating reaction conditions.^[14]^ **Ir‐1** species contains an octahedral iridium(III) atom coordinated to a peripheral *N*,*N*‐chelating moiety, three (pinacolato)boryl ligands and a COD ligand (Scheme 1). From the many possibilities, the *fac*‐coordination of the boryl ligands to the iridium atom is the more plausible as discussed by Hartwig.^[17]^ At this stage, the binding of pyridine to zinc below the porphyrin plane, that is the opposite site from the catalytically active iridium site, appears energetically more feasible (Δ*G*=−3.2 kcal mol^−1^, Scheme 1) than the binding above the plane (Δ*G*=+5.1 kcal mol^−1^, Scheme 1). This is hardly surprising since the binding of pyridine in the same face that the active iridium species will result in a highly sterically demanding system (Scheme 1). Interestingly, such large difference in energy regarding the binding of pyridine above and below the porphyrin plane of ΔΔ*G*=8.3 kcal mol^−1^ for **Ir‐1** is reduced to only ΔΔ*G*=0.4 kcal mol^−1^ (**A** vs. **A’**, Scheme 1), when the binding of pyridine to zinc occurs after the release of the labile COD ligand previously coordinated to iridium, that is from species **A0** (Scheme 1). As such, once the COD ligand is released from the iridium center (**A0**), the binding of pyridine from the same face (**A**) or the opposite one (**A’**) is energetically similar. For comparison purposes, we calculated the energy associated to the binding of pyridine to zinc when the system lacks the iridium species (see Figure S2 in the Supporting Information), which is known to occur in solution and in the solid state as shown previously.^[14]^ In this case, a Δ*G*=−2.3 kcal mol^−1^ is obtained that is significantly higher in energy than that observed with the binding of pyridine to the iridium‐coordinated species (**A0**→**A**) with a Δ*G*=−5.1 kcal mol^−1^ (Scheme 1).^[16]^

**Figure 2:**
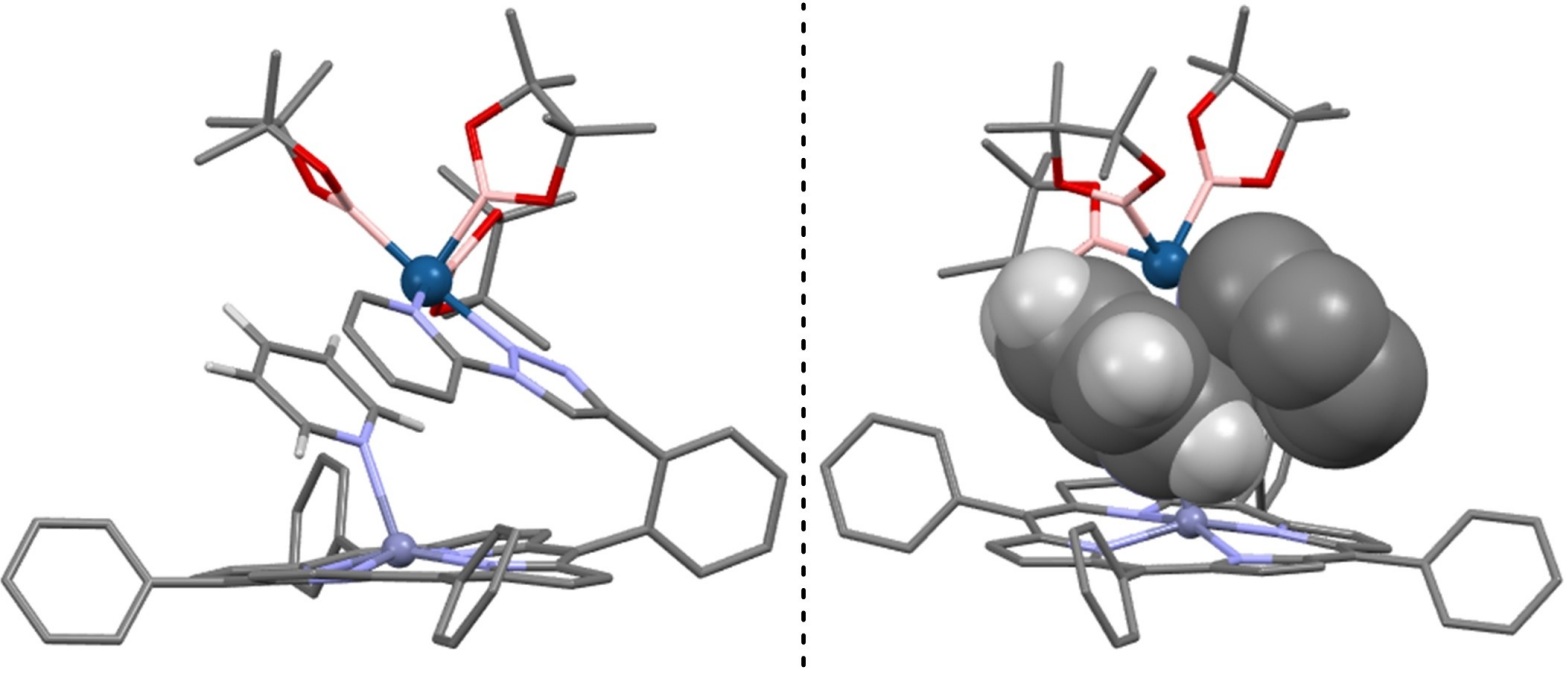
Molecular structure of the computed intermediate **A** (left, and with the fragments involved in π–π stacking in space‐filling representation, right). For the sake of clarity, all hydrogen atoms have been omitted except those belonging to the pyridine substrate, and the zinc and iridium atoms are shown in ball & stick representation.

A careful study of the molecular structure of the catalytically productive intermediate **A** (Figure 2) revealed a Zn⋅⋅⋅N distance of 2.191 Å with a Mayer Bond Order (MBO) of 0.122 (Table 1). The Zn⋅⋅⋅N distance in **A** is larger than that found experimentally in the X‐ray structure of the binding of pyridine to the supramolecular catalyst lacking the iridium site (d_Zn⋅⋅⋅N_=2.135 Å) which also displays an almost ideal binding of the pyridine substrate to the zinc center with an angle Zn−N‐C_*para*_=179°,^[14]^ as it could be expected.^[18]^ Notably, the weakening of the Zn⋅⋅⋅N binding in **A** is additionally followed by a strong deviation from the linearity in the binding of pyridine to the zinc center with an angle Zn−N‐C_*para*_=151° (Table 1). This highly distorted binding, that deviates almost 30° from the ideal case (see deviation angle definition in Table 1), is compensated by π⋅⋅⋅π interactions^[19]^ between the pyridine substrate and the iridium‐coordinated pyridine moiety from the supramolecular catalyst in the periphery (Figure 2, right). Intermediate **A** also indicated that the closest pyridinic C−H bond to iridium was the one in *meta* position with a distance Ir⋅⋅⋅C_*meta*_ of 2.620 Å compared to those in *ortho* (d_Ir⋅⋅⋅C*ortho*=_2.696 Å) and *para* (d_Ir⋅⋅⋅C*para*_=3.337 Å) positions. Although at first glance both *meta* and *ortho* C−H bonds seem accessible regarding the Ir⋅⋅⋅C−H distances, the C−H bond in *meta* results less sterically hindered than the C−H bond in *ortho* as shown by the %V_Bur_ descriptor developed by Cavallo (76.7 % for *meta* vs. 94.6 % for *ortho*, see Figure S8 in the Supporting Information).^[20]^ In other words, the *ortho*‐C−H bonds from pyridine are inaccessible for iridium due to the important steric shields provided by the porphyrin backbone. Overall, intermediate **A** appears as an example of a ground state highly destabilized by the synergy of the substrate‐recognition site and the steric effects encountered at the active site. In fact, these findings contrast with the common scenario in which the combination of a substrate S and a catalyst C leads to a more stable system S+C.^[21]^

**Table 1:** Summary of the key parameters relevant for substrate‐binding strength and distortion effects between the catalyst and the ligand encountered for intermediates and transition states involved in the catalytic cycle from **A**→**E**.

Intermediates or transition states	Zn⋅⋅⋅N distance [Å]	MBO^[a]^	Angle Zn−N‐C_*para*_ [°]	Deviation angle [°]^[b]^
**A**	2.191	0.122	151	29
**TS_AB_**	2.100	0.253	176	4
**B**	2.099	0.273	171	9
**TS_BC_**	2.141	0.264	172	8
**C**	2.192	0.117	164	16
**TS_CD_**	2.191	0.113	158	22
**D**	2.175	0.140	164	16
**E**	2.184	0.124	161	19

[a] Mayer Bond Order calculated for the Zn⋅⋅⋅N interaction. [b] Defined as the difference between 180° (ideal Zn−N−C_*para*_) and the observed Zn−N−C_*para*_ angle for a given species.

**Figure 3:**
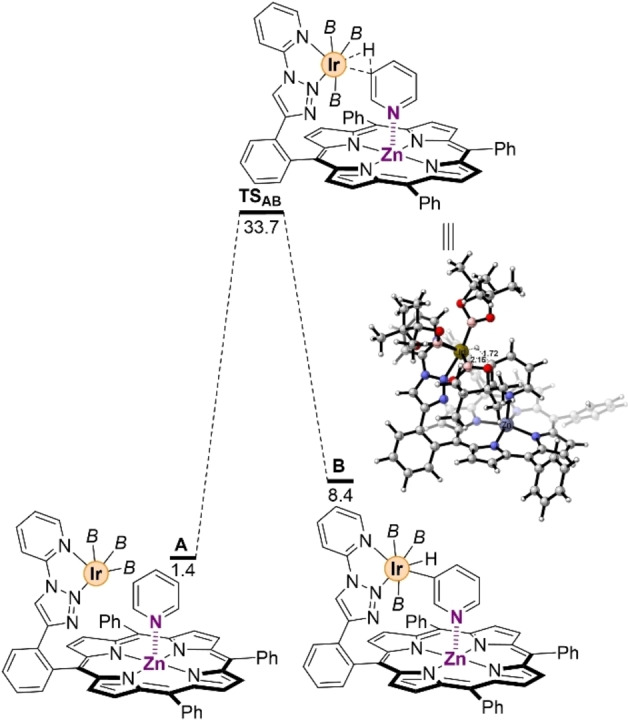
Probing the iridium‐mediated *meta*‐C−H bond activation step in a pyridine substrate. *B*=(pinacolato)boron. Relative Gibbs free energies in kcal mol^−1^, selected distances in Å.

Next, we evaluated the C−H bond activation step (Figure 3). In this case, the tight Zn⋅⋅⋅N interaction anchors the pyridine to the substrate‐recognition site and so, unlike in the mechanism reported by Hartwig,^[17g]^ the pyridine cannot change its orientation but it can only slightly rotate around the axes along the Zn⋅⋅⋅N interaction. Therefore, when the pyridine rotates around the Zn⋅⋅⋅N axes, the C−H bond at the *meta*‐position is activated and cleaved (C−H=1.010 Å in **A** vs. 1.722 Å in **TS_AB_**), and an Ir−H bond is formed (Ir−H=1.765 Å in **B**) through the transition state **TS_AB_** while the Zn⋅⋅⋅N interaction strengthens by 0.092 Å from **A** to **B** (Zn⋅⋅⋅N distance in **B**=2.099 Å, Figure 3 and Table 1). The overall process requires to overcome an energy barrier of 32.3 kcal mol^−1^ (Figure 3), becoming the rate determining step (rds) of the whole reaction pathway (see below). The validity of the computational method in the rds was checked with M06 and M06‐D3 instead of B3LYP−D3, with inappreciable differences of only 0.5 and 1.0 kcal mol^−1^, respectively.^[22]^ Moreover, from the pre‐catalyst **Ir‐1**, the **TS_AB_** increases by 1.4 kcal mol^−1^ up to 33.7 kcal mol^−1^. Thermodynamically, the latter transition state **TS_AB_** leads to a relatively unstable hepta‐coordinated species **B** (Δ*G*=8.4 kcal mol^−1^), in which the Zn⋅⋅⋅N distance remains constant (2.099 Å) with respect to that found in **TS_AB_** with a MBO of 0.253 (Table 1). The angles Zn−N−C_*para*_ were found to be 176° for **TS_AB_** and 171° for **B** indicating that these systems imply significantly less distortion systems compared to **A** (Table 1). Importantly, the fact that the distance is shorter in the **TS_AB_** and **B** rather than in **A** clearly indicates that the pyridine prefers to bind to the molecular‐recognition site during the rds rather than as a free substrate. Consequently, this observation strongly suggests that the supramolecular catalyst follows an induced fit mechanism with differential binding rather than a conformational selection one.^[23]^

For comparison purposes, the alternative reaction pathway in *para* was also calculated. Even though **TS_AB_** ^*para*^ requires to pay only an energetic cost by 20.6 kcal mol^−1^ (see Figure S3 in the Supporting Information), with additional weakening of the Zn⋅⋅⋅N interaction to 2.125 Å compared to the *meta* intermediate,^[16]^ at 80 °C the intermediate **B** ^*para*^ (formed after activation of the C_*para*_‐H bond in pyridine) is 6.9 kcal mol^−1^ less stable than the intermediate **B**. Next, the transition state **TS_BC_** ^*para*^ leading to the iridium‐mediated formation of the C−B bond in *para* position of the pyridine (see Figure S4 in the Supporting Information) becomes kinetically disfavored by 22.3 kcal mol^−1^ with respect to **TS_BC_** ^*meta*^. Contrarily, **B** ^*para*^ needs to overcome a very low kinetic barrier of only 5.3 kcal mol^−1^ to come back to **A**,^[24]^ thus we can assume a reversible process, while the energy barrier for the C−B bond formation in *para* is highly kinetically demanding, 34.1 kcal mol^−1^. To further explain why the *para* C−H activation results less stable than the preferred *meta* one, structurally Ir‐C^*para*^‐N angle is 160.8° and therefore, the linearity of the Ir<C_para_‐>N angle is partially lost unlike in an iridium catalysis not assisted by zinc. The same distortion is present in the **TS_AB_** ^***para***^. In addition, we also performed the calculation for the corresponding *ortho* C−H activation and the energy barrier went up by 16.1 kcal mol^−1^. As additional computations, we calculated the acidity of each pyridine proton upon binding to zinc for a model system lacking the iridium site (see Figure S5 in the Supporting Information). Using this simplification, the *meta* proton of the pyridine bonded to zinc was found to be the most reactive since its removal is favored by 1.0 and 2.7 kcal mol^−1^ with respect to the *para* and *ortho* ones, respectively.^[16]^ In contrast, in the absence of Zn⋅⋅⋅N binding, the removal of the *para* proton of the pyridine is favored by 1.7 and 7.8 kcal mol^−1^ with respect to the *meta* and *ortho* ones, respectively.^[16]^ These findings indicate that there is minimal (almost negligible) modification of the acidity of the hydrogen atoms from pyridine upon binding to the zinc‐porphyrin.^[25]^ However, it is known from experimental results that no C−H borylation takes place if the iridium site is not covalently linked to the substrate‐recognition site.^[14]^ This was further supported by additional computational calculations on the energy costs associated to overcome the rate‐determining step, i. e. **TS_AB_**, using zinc‐porphyrinoids lacking the covalently attached iridium site, namely zinc‐tetraphenylporphyrin and zinc‐salphen (see Figure S6 in the Supporting Information). The calculations show larger activation energy barriers by 9.2 and 11.9 kcal mol^−1^ higher in energy, respectively.^[16]^ In addition, we also computed the **TS_AB_** with a system lacking the zinc‐porphyrin substrate‐recognition site, and the energy barrier increased by 3.1 kcal mol^−1^, which is a significant value considering that the homogeneous catalysis takes place at 80 °C.^[16]^

**Figure 4:**
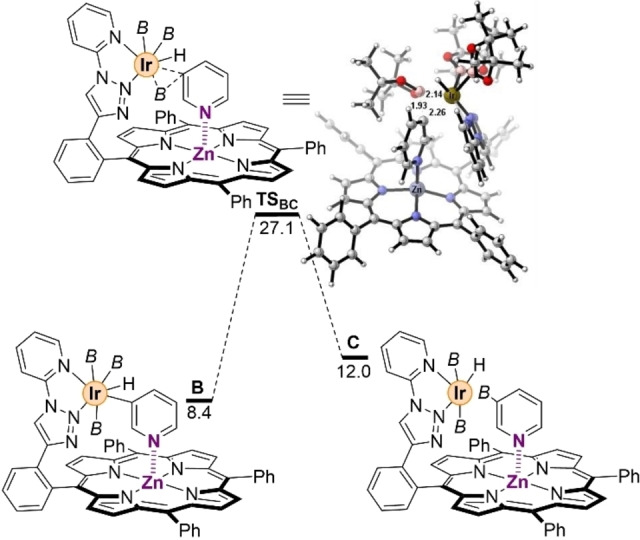
Probing the iridium‐mediated *meta*‐carbon‐borylation step in a pyridine substrate. *B*=(pinacolato)boron. Relative Gibbs free energies in kcal mol^−1^, selected distances in Å.

Unexpectedly, the formation of the hydride species in intermediate **B** results in an elongation of one of the Ir−B bonds from 2.048 Å to 2.179 Å with a formal B−H bonding interaction with the hydride ligand (Figure 4). The hydride interacts with the empty p orbital of the boron moiety, and it makes less reactive this boryl group compared to the other two ones. The next step of the reaction mechanism occurs upon the rotation of the other boryl group located *trans* to the one involved in the B−H interaction leading to the formation of a C−B bond in **TS_BC_**, overcoming an energy barrier of 18.7 kcal mol^−1^, with concomitant weakening (almost cleavage) of the H−B interaction from 1.383 Å to 2.070 Å. In **TS_BC_**, the B−C_*meta*_ distance shortens to 1.927 Å while the Ir−B bond results only quite activated (2.145 Å in **TS_BC_** vs. 2.105 Å in **B**, Figure 4). As a result, the pyridine substrate is borylated at the *meta* position and intermediate **C** is formed, even though the interaction with zinc becomes slightly weaker again (Zn⋅⋅⋅N distance=2.141 Å) but still significant, with an associated MBO of 0.264. The intermediate **C** presents an octahedral geometry at the iridium center with a η^2^‐borylated pyridine as ligand and a weakening of the Zn⋅⋅⋅N distance to 2.193 Å comparable to that found in intermediate **A** (Figure 4). Similarly, a remarkable bending of 16 degrees in the deviation angle was found for the binding of the *meta*‐borylated pyridine to zinc (Table 1), which is also stabilized by π⋅⋅⋅π interactions between the borylated pyridine substrate and the iridium‐coordinated pyridine moiety from the supramolecular catalyst in the periphery. Analogously to **TS_BC_**, no H⋅⋅⋅B interaction is present and the intermediate **C** results 3.7 kcal mol^−1^ less stable than **B**.

**Figure 5:**
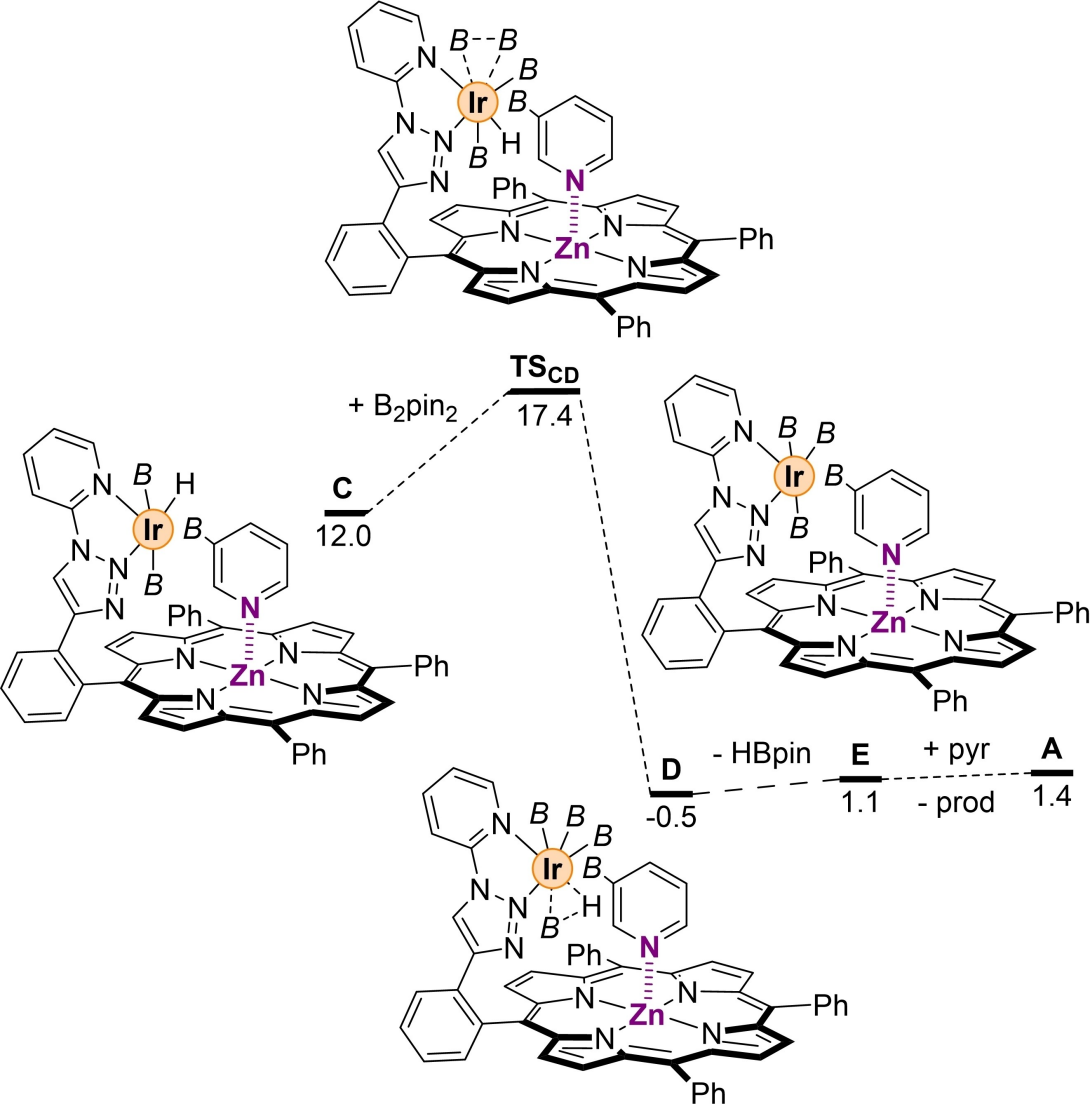
Catalyst regeneration from a η^2^‐coordinated borylated pyridine to iridium and closing of the catalytic cycle. *B*=(pinacolato)boron, pyr=pyridine, prod=3‐borylated pyridine product. Relative Gibbs free energies in kcal mol^−1^.

We then focused on catalyst regeneration. This latter consists mainly of two different elementary steps: (1) the oxidative addition of a molecule of B_2_pin_2_ and (2) the reductive elimination of HBpin at the iridium site (Figure 5). Initially, we considered these two steps to occur without the borylated pyridine product binding to the molecular‐recognition site, namely with an iridium center that has a vacant coordination site since the prior intermediate **C** displays η^2^‐coordination to iridium.^[16]^ The corresponding intermediate formed after product decoordination releases only 2.8 kcal mol^−1^, and the resulting oxidative process is highly kinetically demanding, with an energy barrier of 22.0 kcal mol^−1^, as it could be expected. In fact, the resulting intermediate is rather unstable since this step is thermodynamically endergonic by 20.0 kcal mol^−1^.^[16]^ On the other hand, in the case that the borylated pyridine product is still binding to zinc as well as to iridium, steps (1) and (2) are energetically accessible (Figure 5). Indeed, when B_2_pin_2_ reacts with the iridium center the process yields a highly stable iridium(V)tetrakis(boryl)hydride complex (intermediate **D**, Δ*G*=−0.5 kcal mol^−1^) overcoming an energy barrier of only 5.4 kcal mol^−1^. Comparing this system with the one analyzed by Sakaki,^[26]^ it is noted that the two systems are comparable in energy (5.4 vs. 8.0 kcal mol^−1^). However, while the diboron molecule is added at the vacancy left by the borylated benzene in Sakaki's system,^[26]^ in the present supramolecular system this process takes place on the opposite side. In fact, the change on this crucial elementary step occurs because the borylated pyridine product remains interacting with the zinc center from the molecular recognition site; and the reactive system cannot rotate along the bond that connects the porphyrin backbone to the iridium site. Therefore, the B_2_pin_2_ is added on the side that results to be less hindered. At the approach of the diboron towards the iridium complex, this last one isomerizes into an octahedral hydride complex with a η^2^‐B_2_pin_2_ ligand as shown in the transition state **TS_CD_** (Figure 5 and Table S1 in the Supporting Information) in order to minimize the steric hindrance of the boryl group. Next, a molecule of HBpin is released into solution leading to the formation of intermediate **E** (ΔG=1.1 kcal mol^−1^) with the process facilitated by the fact that one of the Ir−B bond results already activated (2.185 Å in **D** vs. 2.048 Å in **A**) in intermediate **D** and by interaction of the borylated pyridine product to the iridium center. The intermediates and transition states from **C**→**E**, reveal weaker Zn⋅⋅⋅N interactions compared to the one observed in the key transition state **TS_AB_** (see above). In other words, this supramolecular catalyst appears to be designed to better fit the key rate‐determining step rather than the other intermediates or transition states of the catalytic cycle, a feature reminiscent from enzymes, but observed herein for a transition metal catalyst. The catalytic cycle is closed with formation of intermediate **A** after product release and substrate binding to the molecular recognition site of the supramolecular catalyst (Figure 5). As it could be expected from all the above‐stated discussion, we found a linear correlation between the two key descriptors of this study: the Zn⋅⋅⋅N distance and the deviation angle (see Figure S7 in the Supporting Information).

For completing this study, and with the aim to better understand the role of pyridine, the aromaticity of the pyridine ring was analyzed throughout the full catalytic cycle.[^16^, ^27^] Importantly, its binding to zinc first, and later functionalization with iridium, makes the pyridine ring to become less aromatic. Next, consequently, we wondered whether the inclusion of either an electron‐donor or electron‐acceptor group in the pyridine substrate could affect the reaction barrier of the iridium‐catalyzed *meta*‐C−H bond borylation. For such, we embarked in the analysis of the rate‐determining step, namely the energy associated to **TS_AB_**, for a series of 3‐functionalized pyridines as the substrates for the supramolecular iridium‐catalyzed C−H bond borylation reaction (Table 2). Overall, the inclusion of either electron‐donor or electron‐acceptor substituents (Me, OMe, CF_3_, ^*t*^Bu) in the initial substrate led to no major changes associated to the energy of **TS_AB_** (Table 2). However, a trend was roughly established by comparing the ΔG_rds_ and the MBOs (a higher ΔG_rds_ corresponds to a higher MBO), thereby confirming the key role played by the zinc center in its interaction with the pyridine substrates. And such minor role of the inclusion of a substituent is also supported by the analysis of the aromaticity of a series of both substituted intermediate **A** and **TS_AB_** systems (see Table S1 in the Supporting Information).^[16]^ The electron‐withdrawing trifluoromethyl group caused the largest decrease of aromaticity in **A** (MCI_CF3_=0.027), whereas the other systems presented a more constant aromatic pyridine ring regardless of the substituent.

**Table 2:** Selected parameters found for the transition state **TS_AB_** considering different 3‐functionalized‐pyridine substrates.

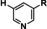	ΔG_rds_ [kcal mol^−1^]	MBO^[a]^	%V_bur_	HOMO (a.u.)	LUMO (a.u.)
R=H	33.7	0.122	58.8	−0.186	−0.092
R=Me	33.2	0.139	63.2	−0.184	−0.090
R=OMe	31.2	0.097	72.6	−0.182	−0.088
R=CF_3_	32.0	0.112	72.9	−0.181	−0.088
R=^*t*^Bu	30.9	0.115	82.6	−0.180	−0.087

[a] Mayer Bond Order calculated for the Zn⋅⋅⋅N interaction.

**Figure 6:**
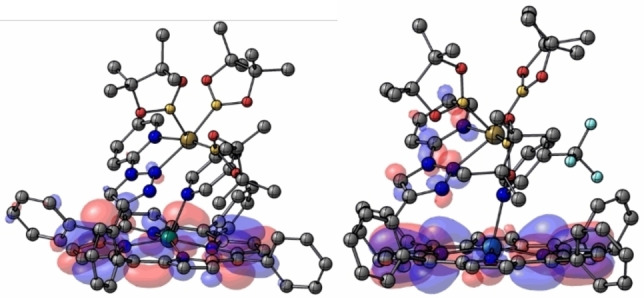
LUMO orbitals for intermediate **A** with pyridine (left) and 3‐(trifluoromethyl)pyridine (right) as the substrates.

Interestingly, the reactivity of the different substituents on 3‐functionalized pyridine substrates, defined by the bottleneck of the energy barrier for the rate‐determining step, can be directly related to the energy of the LUMO of the rds. Actually, there is a linear correlation among both variables (R^2^=0.909).^[16]^ Even though the electronics seem fundamental, they are equally important as the sterics, because the calculation on the metal center of the %V_Bur_, developed by Cavallo,^[20]^ led to an identical good agreement (R^2^=0.901).^[16]^ The additional effect of both contributions led us to apply a multilinear regression to find the equation: ΔG_rds_=−0.122 * %V_Bur_−314.259 * *E*_LUMO_+11.992,^[16]^ even though the short set of data does not allow a clear confirmation of this good correlation (R^2^=0.940). In summary, there is a clear trend in which the larger the substituent is, the lower is the kinetic cost, whereas from an electronic point of view, the electron‐withdrawing groups enlarge the energy barrier of the rate‐determining step. For instance, the pyridine substrate with a *tert*‐butyl substituent is the most sterically hindered, and despite not being the most electron‐donating group, it is the one that requires a lower reaction barrier. Thus, it can be concluded that steric effects are more important than electronic ones, bearing in mind the methoxy group is the most electron‐donating one. However, the electronics also drive the kinetics. To better exemplify this case, the LUMO orbitals for intermediate **A** with pyridine and 3‐trifluoromethyl‐pyridine substrates, respectively, were calculated (Table 2) and displayed in Figure 6 showing significant differences (for the other substituents, see the Supporting Information).^[16]^ In the case of 3‐trifluoromethylpyridine substrate the LUMO is slightly delocalized in the triazolopyridine moiety from the supramolecular catalyst, while in the case of unfunctionalized pyridine, no delocalization beyond the porphyrin backbone is observed.

For the sake of comparison, we placed the substituents in relative *meta*‐position because (1) otherwise, experimentally a second borylation in the second *meta*‐position of pyridine could occur, and (2) *ortho*‐ and *para*‐functionalized pyridine substrates are unreactive with this catalytic system.^[14]^ Nevertheless, we calculated the energy of the transition state of the C−H activation step when a boryl group is present in *meta* position and it is only 22.4 kcal mol^−1^ higher than the isolated reactants since the sterical hindrance plays a key role to support the best substitution.

## Conclusion

In conclusion, we have thoroughly studied the reaction mechanism for the *meta*‐selective C−H bond borylation of pyridines using a supramolecular iridium catalyst by means of DFT calculations underlying the relevance of distortion effects and substrate binding strength as key descriptors. The reaction mechanism for this supramolecular system displayed several features reminiscent from enzymatic behaviors such as the uncommon ground‐state destabilization upon binding the substrate to the catalyst (uphill formation of intermediate **A**) as well as the tight binding of the substrate in the rate‐determining step over the other elementary steps, thereby indicating that the overall structure of the supramolecular catalyst fits better the transition state rather than the substrate and/or the product which is in line with an induced fit mechanism rather than a conformational selection one.[^12^, ^13^, ^22^] In addition, the exquisite levels for *meta*‐selectivity were rationalized by the precise atomic distance between the active site and the substrate binding site that leaves a single C−H bond at close proximity enough to react with the catalytically productive iridium site. Furthermore, we managed to identify an unprecedented pathway for the regeneration of the iridium catalyst thanks to the additional stabilization ensured by the presence of a η^2^‐coordinated product to iridium as well as a new type of C−B bond‐forming elementary step. Additional studies indicate the subtleties associated for correlating the ΔG_rds_ with the different substitution patterns of different pyridine substrates. Overall, this work sheds light on the unexpected reaction mechanisms that might be encountered in supramolecular catalysts aiming at mimicking enzymes, thereby upgrading the rational for the further development of more powerful catalysts tunable at the second coordination sphere.

## Experimental Section

XYZ coordinates and energies of all computed species discussed in this contribution are provided in the Supporting Information.

## Conflict of interest

The authors declare no conflict of interest.

1

## Supporting information

As a service to our authors and readers, this journal provides supporting information supplied by the authors. Such materials are peer reviewed and may be re‐organized for online delivery, but are not copy‐edited or typeset. Technical support issues arising from supporting information (other than missing files) should be addressed to the authors.

Supporting Information

## Data Availability

The data that support the findings of this study are available in the supplementary material of this article.
