# Supplementary material for: Unravelling Enzymatic Features in a Supramolecular Iridium Catalyst by Computational Calculations
Source: Chemistry. 2022 Aug 12;28(57):e202201970. doi: 10.1002/chem.202201970 (PMC9804516; doi:10.1002/chem.202201970)
Supplement: Supplementary file 1 — Supporting Information [file CHEM-28-0-s001.pdf]

Chemistry–A European Journal

Supporting Information

Unravelling Enzymatic Features in a Supramolecular Iridium Catalyst by Computational Calculations

Michele Tomasini, Lucia Caporaso, Jonathan Trouvé, Jordi Poater,* Rafael Gramage-Doria,* and Albert Poater*

Table of Contents

1. Additional Figures…………………………………………………………...S3

2. Aromaticity analyses…………………………………………………………...S7

3. Computational Details and Cartesian Coordinates………………………………..S8

1. Additional Figures

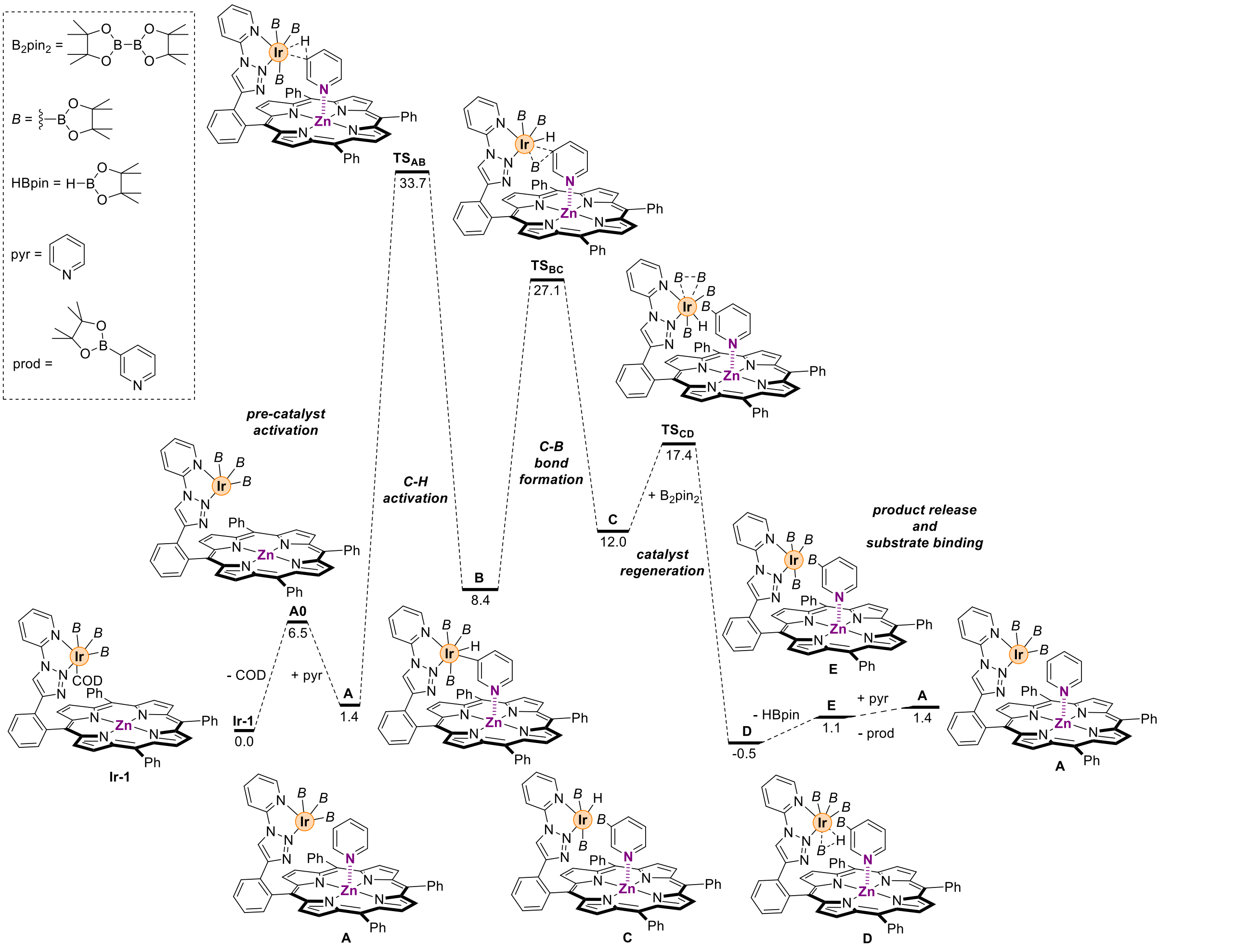

Figure S1. Full catalytic cycle computed for the supramolecular iridium-catalyzed *meta*-C-H bond borylation of pyridine. Relative Gibbs free energies in kcal/mol.

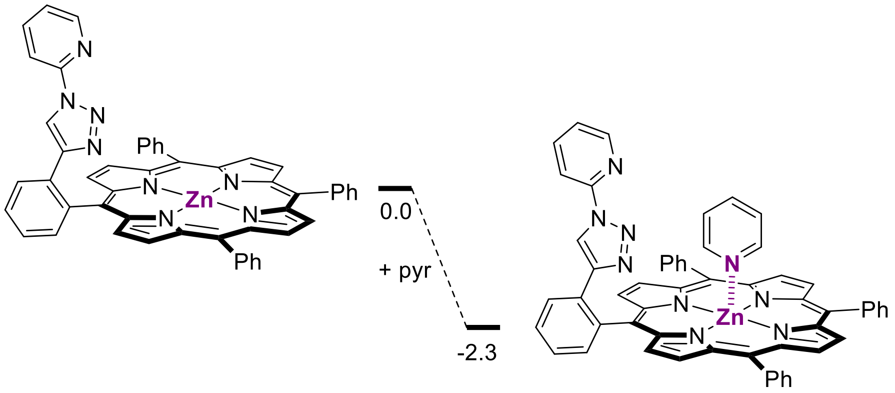

Figure S2. Binding of a pyridine substrate to a ligand system lacking the iridium fragment. The energetic values reported are in kcal/mol. Relative Gibbs free energies in kcal/mol.

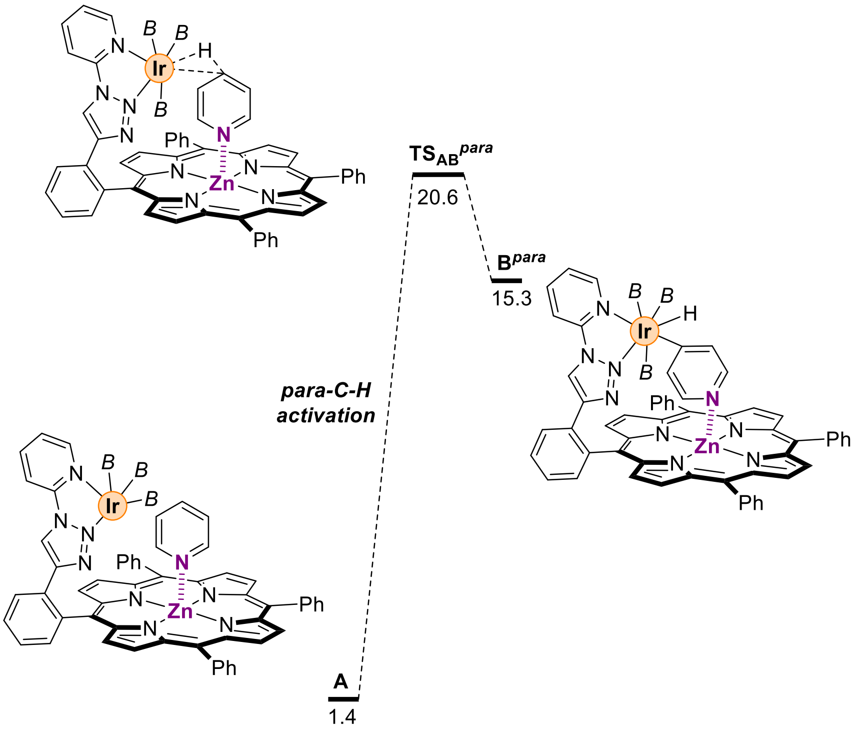

Figure S3. Computational calculations involving a hypothetic pathway for the supramolecular iridium-catalyzed *para*-C-H bond borylation of pyridine. Relative Gibbs energies in kcal/mol.

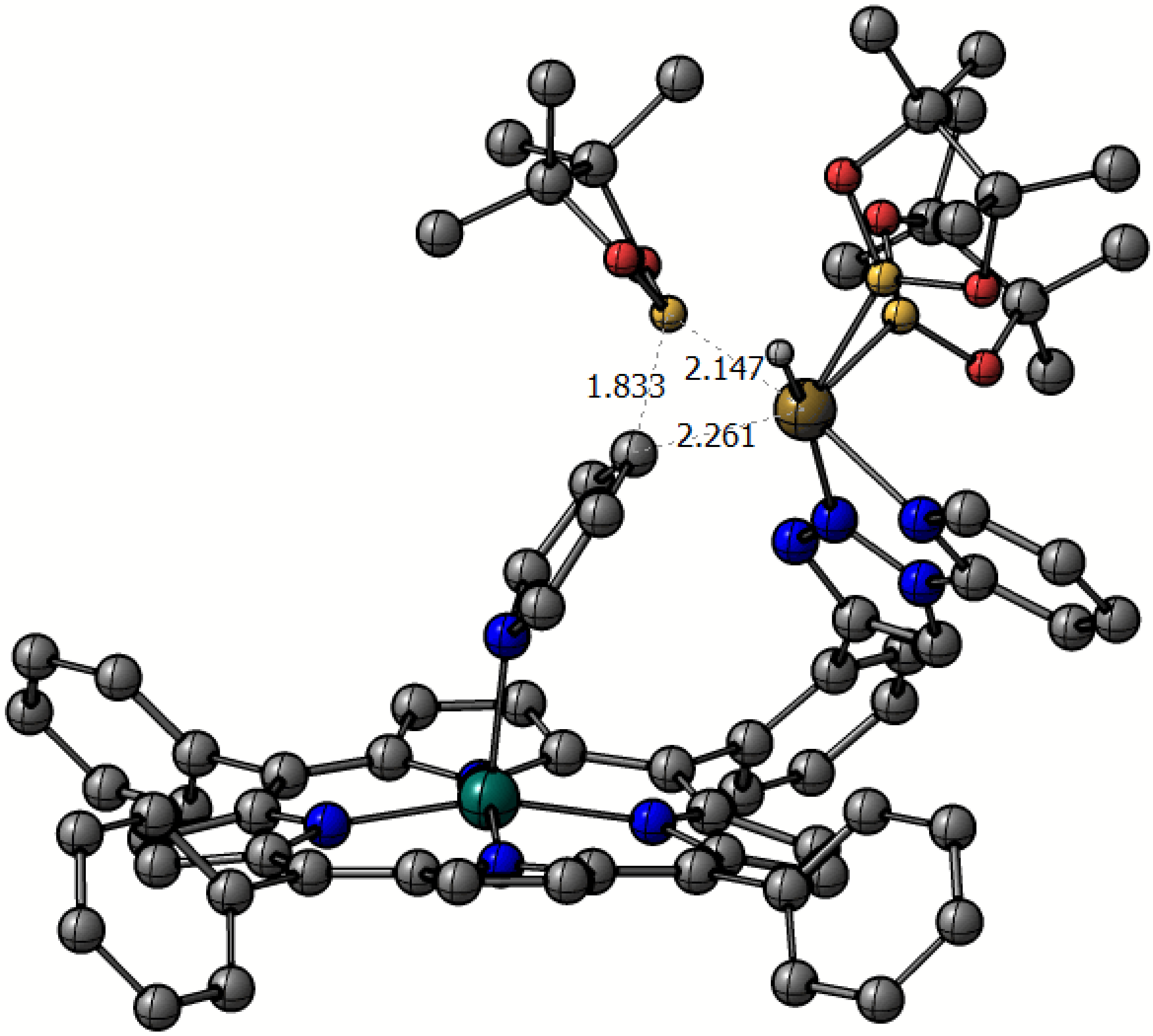

Figure S4. View of the transition state $\mathbf{TS_{BC}}^{para}$ (selected distances are given in Å).

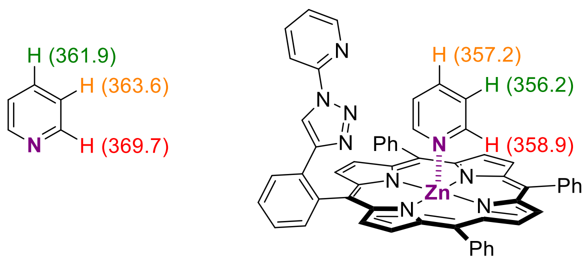

Figure S5. Gibbs energy for the acid dissociation processes of pyridine C-H bonds at 298K in a *p*-xylene solution in the absence or presence of binding to zinc-porphyrin **L**. Relative Gibbs free energies in kcal/mol.

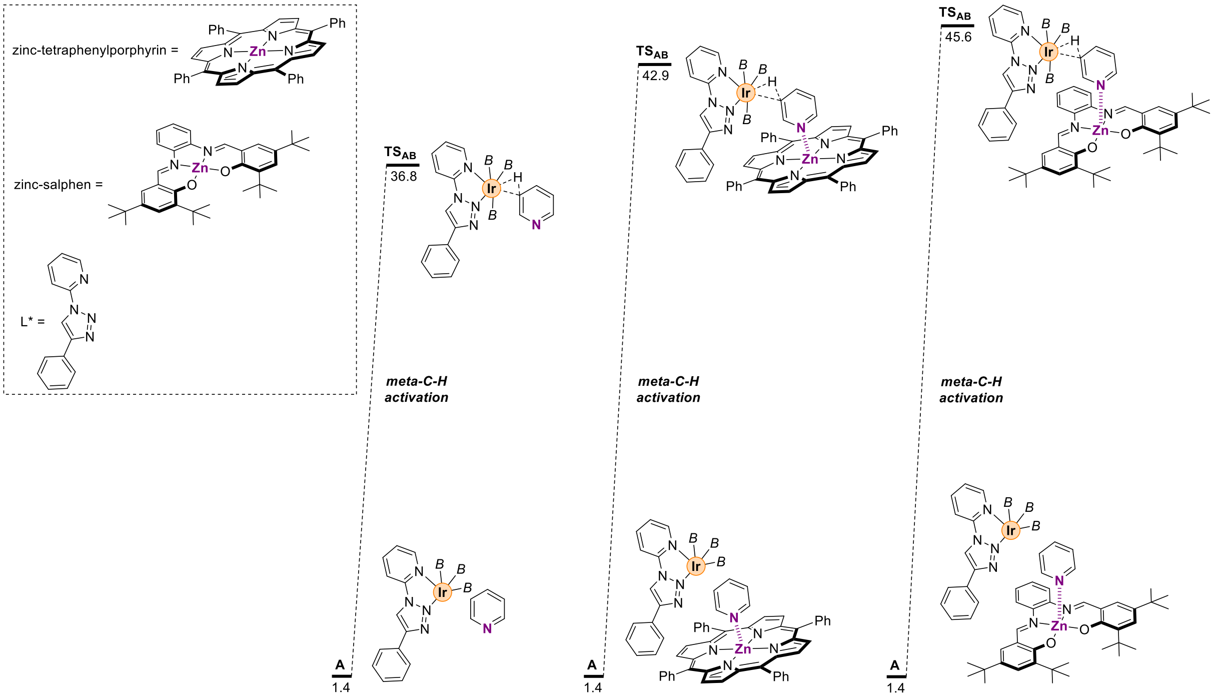

Figure S6. Computational calculations involving a hypothetic key transition state for the iridium-catalyzed *meta*-C-H bond borylation of pyridine using a combination of ligand L* (left), L* and zinc-tetraphenylporphyrin (middle) and L* and zinc-salphen. Relative Gibbs free energies in kcal/mol.

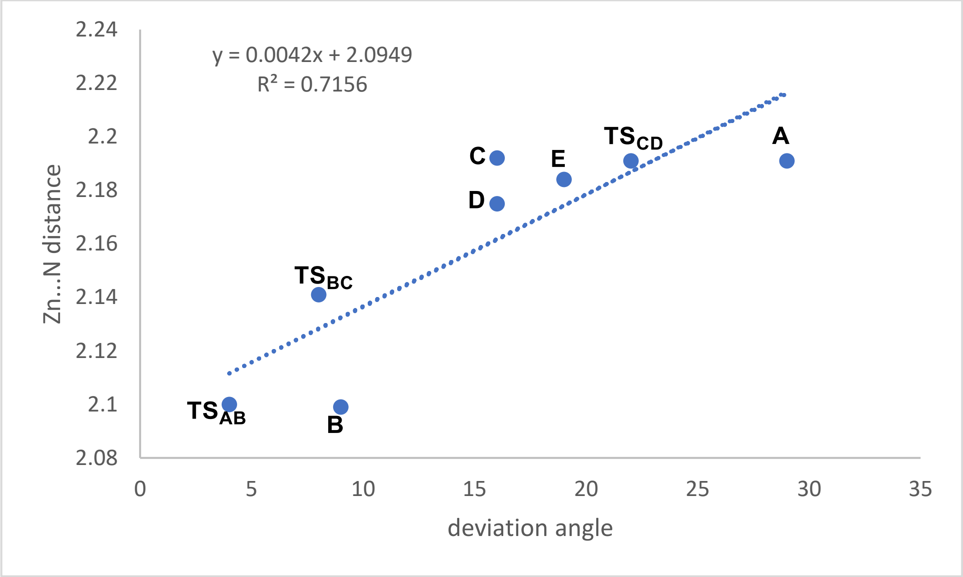

Figure S7. Plot of two descriptors: Zn…N distance *versus* deviation angle (see Table 1 in the main text) for all the intermediates and transition states involved in the supramolecular catalysis.

2. Aromaticity analyses

The aromaticity of the ring among iridium and zinc was analyzed throughout the full catalytic cycle by means of electronic MCI, geometric HOMA and magnetic NICS based aromaticity criteria (see Table S1). Importantly, its binding to zinc first, and later functionalization with iridium, makes the pyridine ring to become less aromatic. Thus, although the pyridine ring remains aromatic, its aromaticity ranges from a maximum MCI of 0.053 in intermediate **B** to a minimum MCI of 0.035 in intermediate **C**, with that of **A** in between (0.041).

Table S1. HOMA, MCI and NICS(0) aromaticity criteria computed for the different systems under analysis at the ZORA-BP86/TZ2P level of theory with ADF on the optimized geometries computed in Gaussian.

	HOMA	MCI	NICS(0)
**pyridine**	0.937	0.070	-6.4
**B**	0.883	0.041	-12.0
**TS_{BC}^{para}**	0.922	0.054	-10.0
**TS_{BC}^{meta}**	0.925	0.049	-9.6
**C**	0.922	0.053	-10.2
**TS_{CD}**	0.903	0.049	-10.3
**D**	0.773	0.035	-11.1
**borylated pyridine**	0.905	0.067	-5.8
**TS_{BC}^{para}**	0.922	0.054	-10.0
**TS_{BC}^{meta}**	0.925	0.049	-9.6
**$TS_{BC,CF3}$**	0.923	0.046	-10.4
**$TS_{BC,OMe}$**	0.889	0.037	-10.5
**$TS_{BC,Me}$**	0.919	0.046	-9.9
**$TS_{BC,tBu}$**	0.927	0.045	-10.3
**$TS_{BC,ZnTPP}$**	0.923	0.053	-10.2
**$TS_{BC, ZS}$**	0.920	0.044	-6.2
**CF_3-pyridine**	0.937	0.066	-7.1
**$B_{CF3, anticlockwise}$**	0.652	0.027	-11.3
**$B_{CF3, clockwise}$**	0.870	0.037	-12.7
**OMe-pyridine**	0.909	0.058	-7.4
**$B_{OMe,anticlockwise}$**	0.879	0.038	-11.9
**$B_{OMe,clockwise}$**	0.867	0.035	-13.1
**Me-pyridine**	0.920	0.066	-6.5
**$B_{Me,anticlockwise}$**	0.860	0.041	-12.2
**$B_{Me,clockwise}$**	0.882	0.040	-12.3
**tBu-pyridine**	0.914	0.065	-6.7
**$B_{tBu,anticlockwise}$**	0.833	0.041	-11.4
**$B_{tBu,clockwise}$**	0.883	0.041	-12.3

3. Computational Details and Cartesian Coordinates

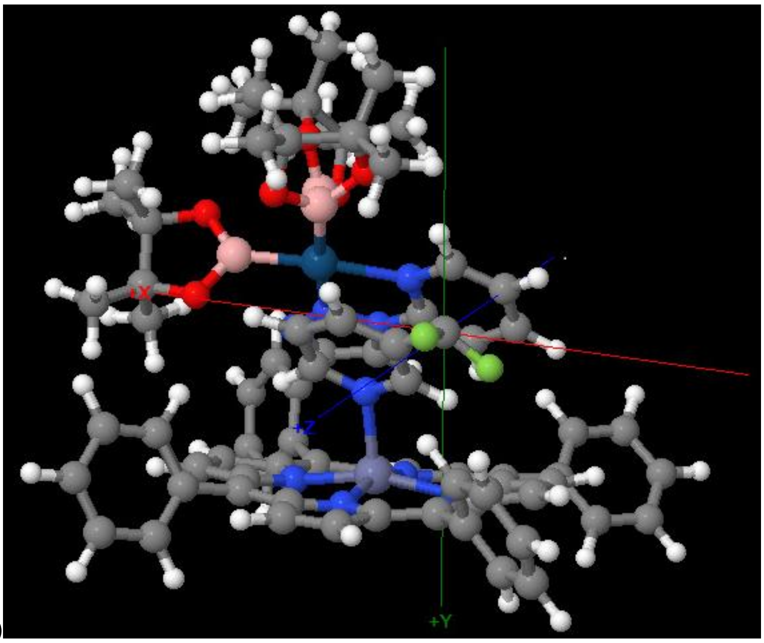

a)

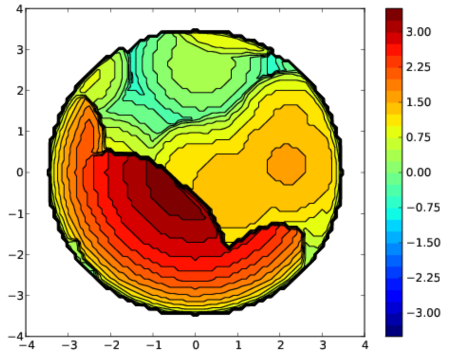

b) *Meta*:

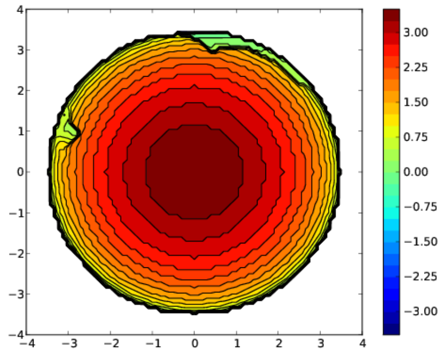

Ortho:

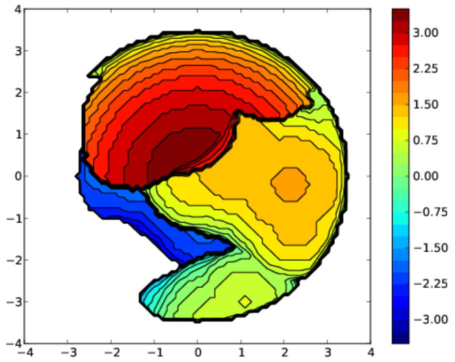

c) H:

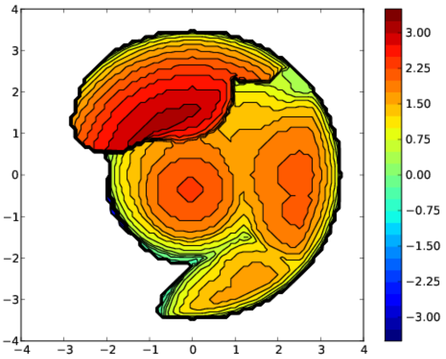

Me:

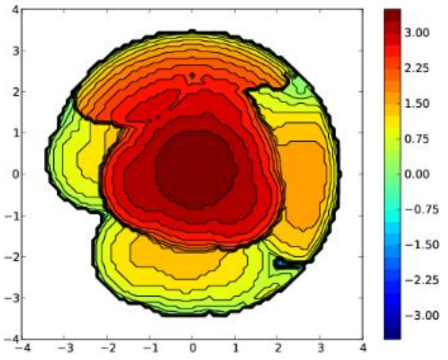

*ᵗ*Bu:

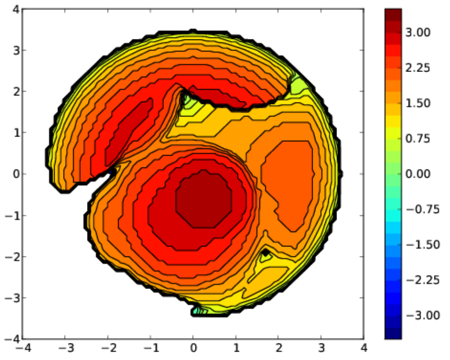

OMe:

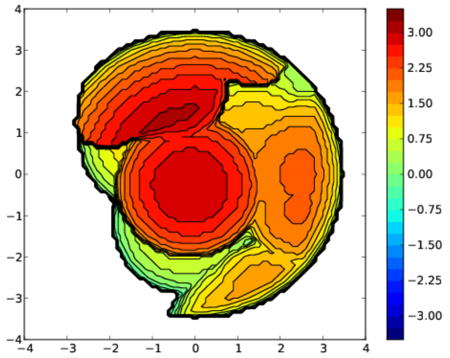

CF_3:

Figure S8. a) Orientation of the axes for the calculation of the %V_{Bur} values on the xy plane; b) Steric maps of intermediate A, for the pyridinic C-H bond closest to iridium, in *meta* and *ortho*; c) Steric maps of the transition state **TS_{AB}** considering different 3-functionalized-pyridine substrates: with R = H, Me, ^{t}Bu, OMe, and CF_3 (with a radius of 3.5 Å, the isocontour curves of the steric maps are given in Å).

R	LUMO	
H	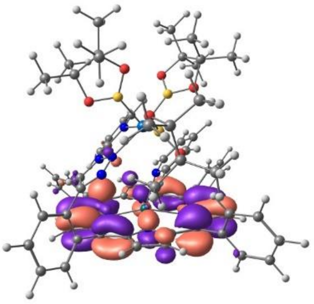	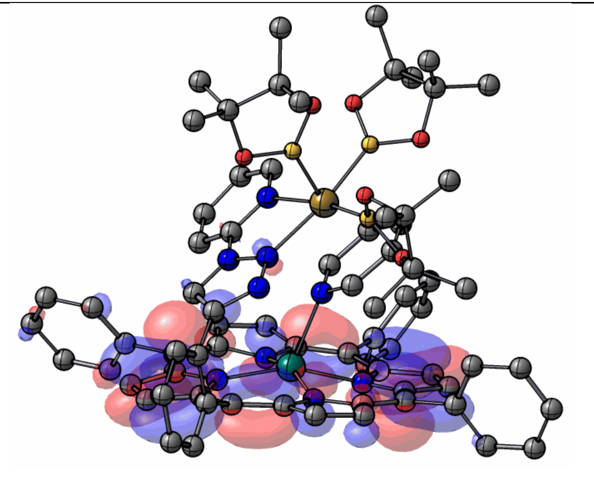
CF_3	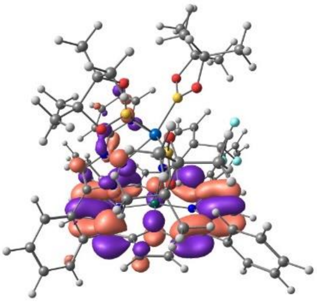	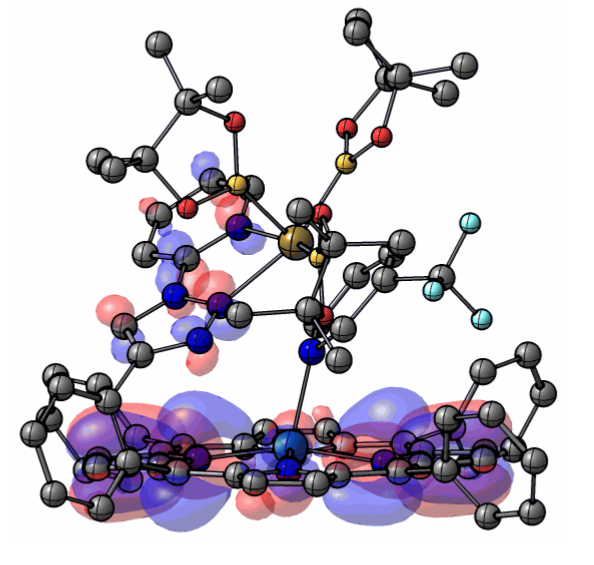
CH_3	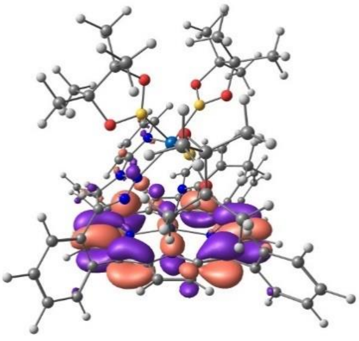	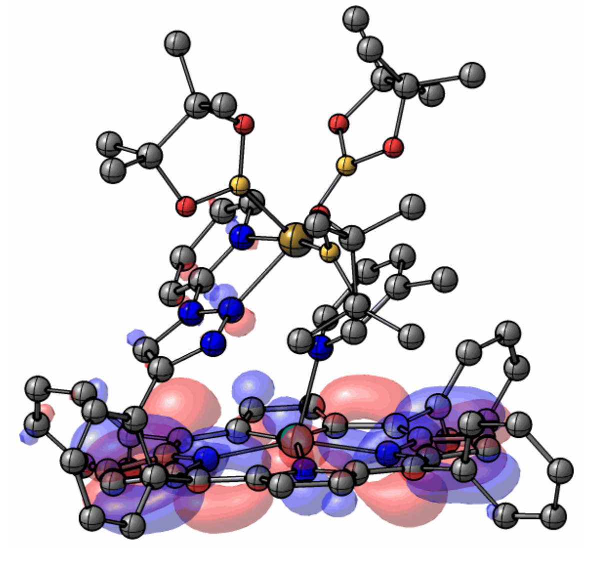

OCH_3	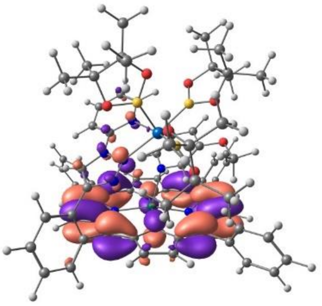	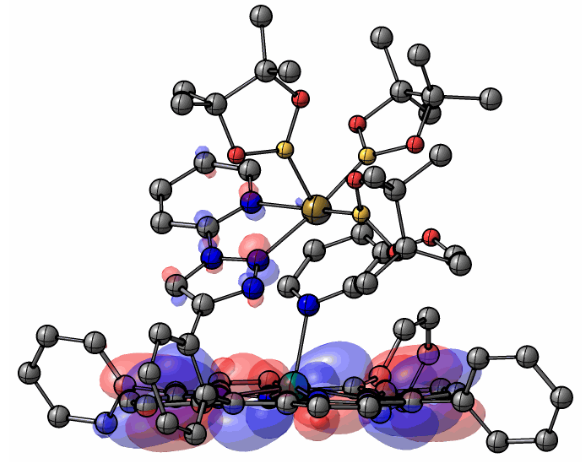
^{t}Bu	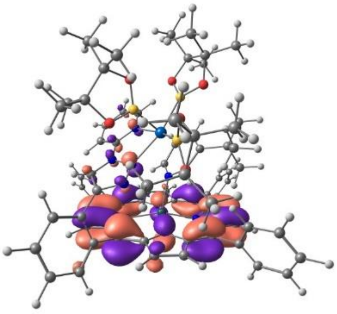	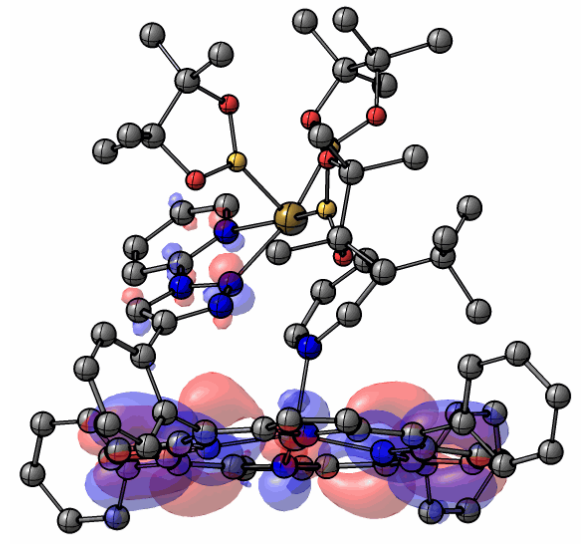

Figure S9. Views of the LUMO orbitals for intermediate **A**, with different R substituents (R = H, CF_3, CH_3, OCH_3, and ^{t}Bu).

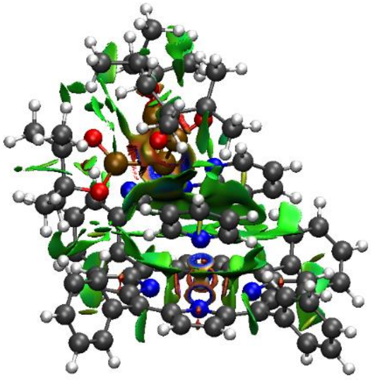

Int A

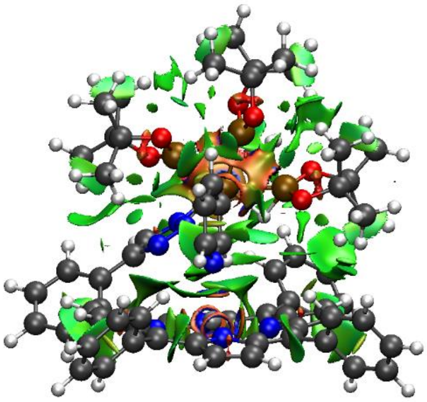

Int B

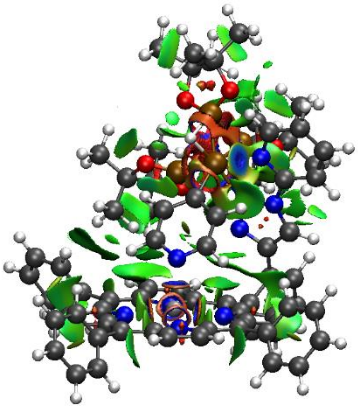

Int B para

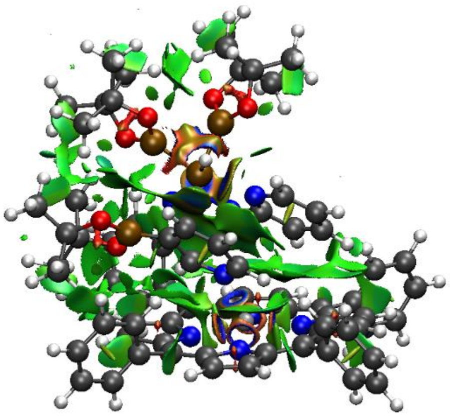

Int C

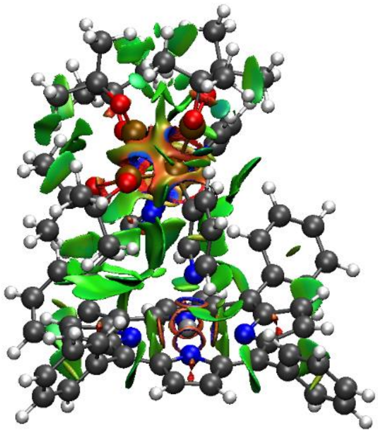

TS AB - meta

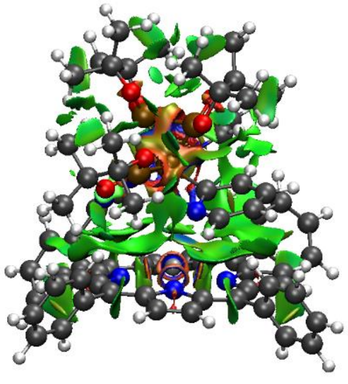

TS AB - ortho

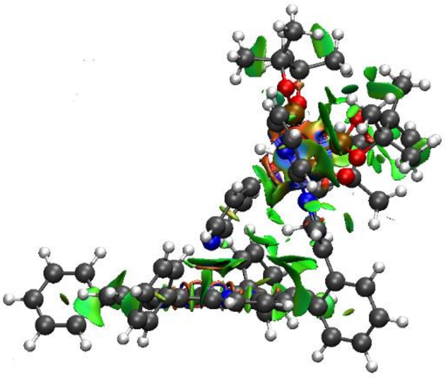

TS AB - para

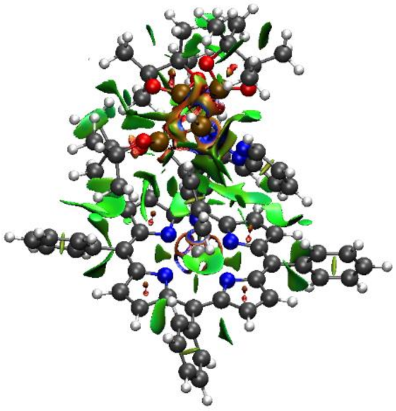

TS BC - meta

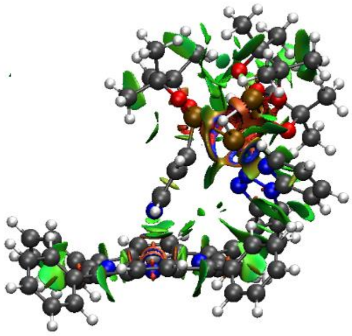

TS BC – para

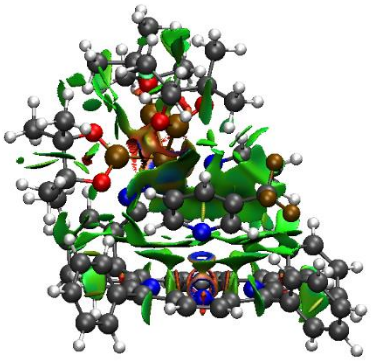

Int A – CF3

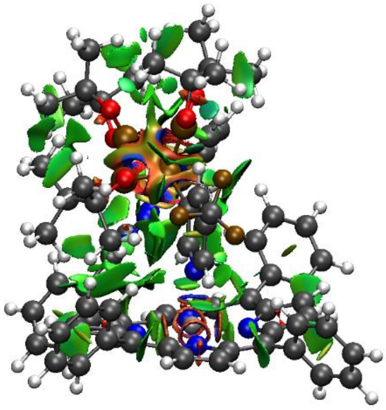

TS AB – CF3

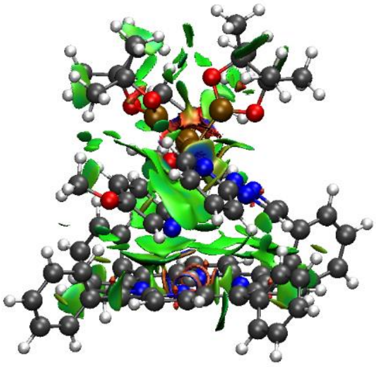

Int A - OMe

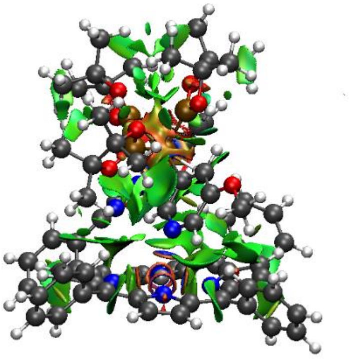

TS AB – OMe

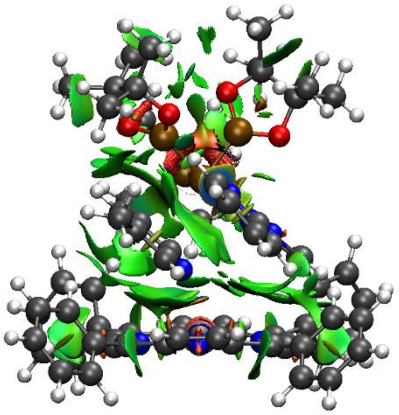

Int A - Me

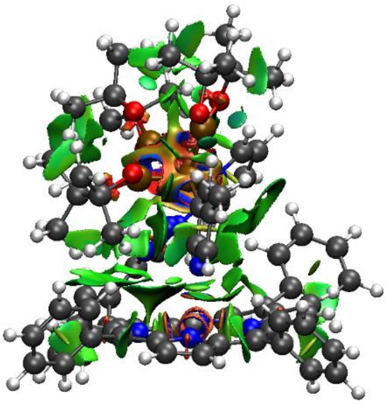

TS AB – Me

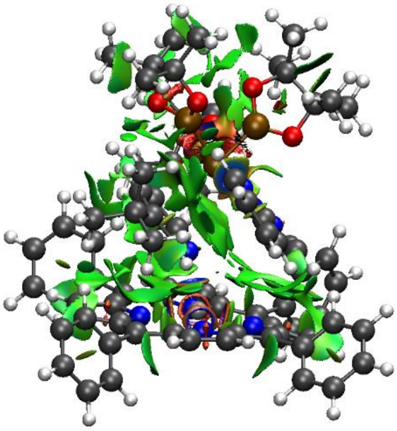

Int A - tBu

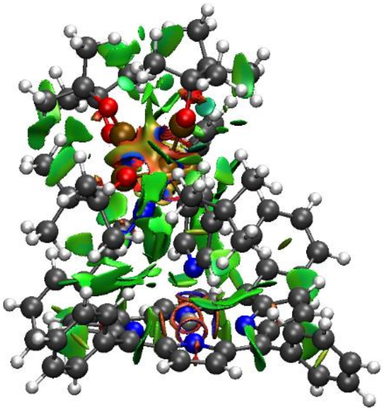

TS AB – tBu

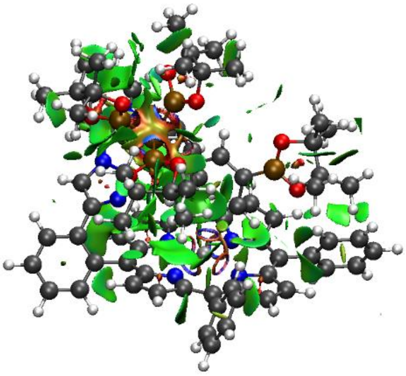

TS AB - Bpin

Figure S10. NCI plots for a selected list of intermediates and transitions states. The isosurface represents a value of 0.5 with a colour scale for the reduced density gradient from -0.05 to 0.05.

Table S2. xyz coordinates and absolute energies of all optimized geometries.

Pyridine

Zero-point correction= 0.086037 (Hartree/Particle)
Thermal correction to Energy= 0.092077
Thermal correction to Enthalpy= 0.093196
Thermal correction to Gibbs Free Energy= 0.053116

solvent: -248.388239

C	0.480082	0.000000	-0.467292
C	0.480082	1.206991	0.251478
C	0.480082	1.148731	1.658211
N	0.480082	0.000000	2.358613
C	0.480082	-1.148731	1.658211
C	0.480082	-1.206991	0.251478
H	0.480082	-0.000000	-1.569245
H	0.480082	2.179561	-0.265681
H	0.480082	2.082221	2.251298
H	0.480082	-2.082221	2.251298
H	0.480082	-2.179561	-0.265681

COD

Zero-point correction= 0.174467 (Hartree/Particle)
Thermal correction to Energy= 0.185061
Thermal correction to Enthalpy= 0.186180
Thermal correction to Gibbs Free Energy= 0.135201

solvent: -312.146379

C	-1.487188	4.692933	1.375935
C	-1.202741	3.401471	1.095592
C	-0.013038	2.827985	0.351294
C	1.252009	2.669547	1.228127
C	1.864115	3.914273	1.826332
C	1.658700	5.219629	1.541374
C	0.715353	5.835596	0.527087
C	-0.742586	5.960915	1.029935
H	-2.417022	4.872504	1.948296
H	-1.911875	2.650499	1.490726
H	2.601823	3.700891	2.623002
H	2.226552	5.946060	2.151931
H	-0.286076	1.821101	-0.030106
H	0.227261	3.426951	-0.548978
H	1.017332	1.973791	2.066881
H	2.037217	2.138567	0.637322
H	1.076103	6.857484	0.283269
H	0.734947	5.281364	-0.431747
H	-0.751425	6.613514	1.933643

H	-1.338904	6.525464	0.272845

B_2pin_2

Zero-point correction= 0.351761 (Hartree/Particle)
Thermal correction to Energy= 0.379475
Thermal correction to Enthalpy= 0.380593
Thermal correction to Gibbs Free Energy= 0.290388

solvent: -822.918304

B	-3.746289	6.412156	5.448946
C	-3.752023	5.228665	7.415478
C	-5.147912	5.081289	6.686784
B	-3.186671	7.411992	4.175063
C	-2.407699	8.034555	2.107825
C	-2.558333	9.304329	3.038370
C	-1.341281	10.229029	3.068592
H	-1.127588	10.626680	2.054846
H	-1.538573	11.087331	3.741942
H	-0.441587	9.705833	3.442948
C	-3.837694	10.108692	2.758485
H	-3.991605	10.833549	3.582560
H	-3.770860	10.669339	1.804326
H	-4.723899	9.443961	2.719970
C	-0.958724	7.533192	2.001257
H	-0.961736	6.528450	1.533612
H	-0.334263	8.208000	1.381666
H	-0.495876	7.440199	3.004182
C	-3.027481	8.177135	0.717538
H	-2.551768	9.011312	0.161370
H	-2.872097	7.243766	0.139885
H	-4.116508	8.361979	0.774079
C	-3.129347	3.914346	7.886647
H	-2.158982	4.115601	8.383536
H	-3.793290	3.407102	8.616967
H	-2.939982	3.227258	7.040841
C	-3.774117	6.253525	8.560593
H	-4.327727	5.873214	9.442734
H	-2.731161	6.470721	8.865831
H	-4.237040	7.205677	8.232348
C	-6.367491	5.329304	7.574638
H	-7.295503	5.203219	6.981428
H	-6.392975	4.604851	8.414873
H	-6.367833	6.354854	7.988915
C	-5.296248	3.752139	5.929446
H	-5.433610	2.897051	6.621714
H	-6.182574	3.815275	5.267213
H	-4.410140	3.556841	5.292811
O	-2.724303	8.701375	4.360696
O	-3.149054	7.016029	2.851227

O	-2.910897	5.798054	6.362791
O	-5.081350	6.133493	5.672712

HBpin

Zero-point correction= 0.184452 (Hartree/Particle)
Thermal correction to Energy= 0.197800
Thermal correction to Enthalpy= 0.198919
Thermal correction to Gibbs Free Energy= 0.142987

solvent: -412.057475

B	1.714190	0.128961	15.052543
O	2.563091	1.118399	15.486168
O	1.203985	-0.653712	16.059863
C	2.476026	1.148654	16.947354
C	1.923473	-0.299394	17.284750
C	3.035857	-1.345486	17.456831
C	0.950062	-0.357322	18.461960
C	1.488291	2.270610	17.303656
C	3.860838	1.456060	17.516897
H	0.048713	0.256131	18.276478
H	1.440205	-0.000021	19.390993
H	0.620539	-1.403037	18.625213
H	2.579282	-2.355195	17.463419
H	3.589679	-1.203248	18.406456
H	3.756667	-1.303483	16.615799
H	3.850002	1.390973	18.624451
H	4.163218	2.484337	17.234095
H	4.626091	0.759281	17.126877
H	1.837701	3.214950	16.840885
H	0.476298	2.051812	16.907836
H	1.416016	2.421151	18.399490
H	1.439676	-0.038220	13.886189

Borylated pyridine

Zero-point correction= 0.252758 (Hartree/Particle)
Thermal correction to Energy= 0.272782
Thermal correction to Enthalpy= 0.273900
Thermal correction to Gibbs Free Energy= 0.200867

solvent: -659.264811

C	1.253025	4.142128	-0.050203
C	2.725053	1.953426	0.696201
C	1.320564	2.960685	-0.813898
C	1.976995	4.200158	1.149541
C	2.742172	3.081831	1.552762
N	2.039867	1.879604	-0.458477
B	3.567653	3.083671	2.876945
H	3.308969	1.056495	0.974702

H	0.762140	2.884225	-1.765930
H	0.645388	4.993595	-0.395660
H	1.957300	5.104126	1.780334
O	3.556797	4.137755	3.767849
O	4.375806	2.037224	3.269201
C	4.613190	3.879806	4.744886
C	4.783076	2.306379	4.646717
C	5.854004	4.649039	4.265399
C	4.158735	4.392267	6.110984
C	6.211474	1.801121	4.847049
C	3.807430	1.539073	5.552582
H	3.171104	3.981365	6.392057
H	4.072955	5.497255	6.088522
H	4.895420	4.118758	6.894173
H	5.587658	5.717935	4.144392
H	6.202339	4.270777	3.283288
H	6.689005	4.574892	4.990635
H	6.899049	2.209480	4.082987
H	6.233342	0.695769	4.766731
H	6.586736	2.082220	5.852588
H	4.088100	1.621804	6.621721
H	2.770851	1.911751	5.427616
H	3.821345	0.468421	5.267128

Ir-1

Zero-point correction= 1.392724 (Hartree/Particle)
Thermal correction to Energy= 1.515458
Thermal correction to Enthalpy= 1.516576
Thermal correction to Gibbs Free Energy= 1.224983

solvent: -5832.27171012

N	2.720589	-0.716300	-0.480008
C	3.150229	-0.453300	-1.767668
C	3.796415	-1.261770	0.197367
C	4.542362	-0.845728	-1.909289
C	3.790329	-1.719867	1.539867
C	2.661582	-1.705119	2.398277
N	1.399118	-1.252804	2.064137
C	0.628923	-1.334099	3.207579
C	1.425677	-1.866112	4.299187
C	2.682192	-2.105178	3.796632
C	-0.725052	-0.941606	3.321001
C	-1.532585	-0.466269	2.260140
N	-1.117019	-0.257117	0.960528
C	-2.223829	0.168113	0.248959
C	-2.951419	-0.172604	2.374415
C	-3.381245	0.215858	1.126883
C	-2.238028	0.533303	-1.118708
C	-1.115677	0.499148	-1.983098

N	0.156858	0.073057	-1.644757
C	0.960514	0.309966	-2.744959
C	-1.117587	1.011762	-3.341997
C	0.168975	0.898701	-3.813207
C	2.353833	0.058737	-2.823563
C	3.351471	-0.097706	-6.510825
C	4.038178	1.345050	-4.201365
C	2.705189	-0.370717	-5.293913
C	4.342553	0.898027	-6.577256
C	4.683974	1.618249	-5.418455
C	3.038879	0.347557	-4.122442
C	-5.176203	2.875350	-1.771627
C	-4.268493	0.357360	-2.624488
C	-5.920941	2.145869	-2.716217
C	-3.981948	2.348638	-1.252092
C	-3.517376	1.080861	-1.672050
C	-5.463304	0.886028	-3.142226
C	-1.371941	-1.059900	4.665373
C	-2.689736	-1.341869	7.160039
C	-1.652265	-2.335657	5.200719
C	-1.754733	0.094539	5.403558
C	-2.412219	-0.067089	6.645895
C	-2.303728	-2.483004	6.435413
C	6.197684	-1.392997	2.237146
C	6.396315	-4.071821	3.054249
C	5.075817	-2.242843	2.099429
C	7.400018	-1.874262	2.779820
C	7.503224	-3.215070	3.191588
C	5.193095	-3.589954	2.512619
C	-1.491819	1.461981	4.917097
C	-2.336755	2.563322	5.006270
H	-3.360596	2.664009	5.372629
N	-0.297426	1.867367	4.363231
N	-0.357988	3.149868	4.134751
N	-1.609498	3.610356	4.502347
C	-1.879080	4.985941	4.470824
C	-2.201548	7.708323	4.575607
C	-3.129143	5.497222	4.869699
C	-0.999340	7.120257	4.179619
C	-3.296030	6.884184	4.912178
N	-0.830332	5.770875	4.102372
Ir	1.127719	4.727354	3.928433
Zn	0.804422	-0.491538	0.245280
B	2.716956	3.639342	4.662231
B	0.978896	4.870376	6.009633
B	2.390958	6.330865	4.066155
O	1.953440	7.668613	4.160179
O	3.792188	6.301262	4.014705
C	4.277089	7.584429	4.482708
C	4.406590	7.473907	6.012201
C	5.630841	7.875219	3.836999

C	3.105385	8.546339	4.047777
C	2.893674	9.756688	4.957628
C	3.204696	8.981407	2.576690
O	1.144614	6.017832	6.781880
O	0.484470	3.813511	6.786509
C	0.996311	5.652591	8.184313
C	0.132094	4.328643	8.094757
C	0.465060	3.264189	9.140593
C	-1.381752	4.600183	8.061644
C	0.320546	6.810447	8.919953
C	2.409057	5.407969	8.732492
H	-3.384605	2.921173	-0.523584
H	-5.523515	3.867362	-1.440853
H	-6.857182	2.560341	-3.122970
H	-6.042040	0.308693	-3.880930
H	-3.906212	-0.629378	-2.953682
H	-3.947572	4.819013	5.146353
H	-4.260720	7.316614	5.217634
H	-2.278604	8.804689	4.614270
H	-0.098135	7.706415	3.937701
H	-1.623541	5.379392	7.310843
H	-1.775469	4.921286	9.047319
H	-1.899053	3.666111	7.759573
H	-0.177798	2.373948	8.984154
H	0.291293	3.644919	10.168560
H	1.518404	2.937431	9.053414
H	2.397754	5.132088	9.806386
H	3.005200	6.334844	8.616546
H	2.910215	4.617396	8.143602
H	0.994891	7.690512	8.928523
H	-0.621652	7.113208	8.424888
H	0.100836	6.533968	9.972298
H	3.414516	7.271583	6.463713
H	4.845583	8.387196	6.463612
H	5.055420	6.607823	6.248322
H	5.981832	8.895868	4.096567
H	6.381955	7.147237	4.205267
H	5.583696	7.782939	2.735067
H	3.807199	10.386210	4.995843
H	2.632888	9.440626	5.985009
H	2.062210	10.381669	4.571338
H	4.017751	9.717958	2.413482
H	3.382497	8.105059	1.921155
H	2.244756	9.446465	2.273342
H	-1.989714	1.430042	-3.856967
H	0.555691	1.210061	-4.789910
H	4.299579	1.910831	-3.293307
H	5.455958	2.403006	-5.462885
H	4.849190	1.112136	-7.531703
H	3.083060	-0.671141	-7.412469
H	1.934239	-1.155166	-5.237126

H	5.128824	-0.774633	-2.832077
H	4.326622	-4.260225	2.399202
H	6.471189	-5.125708	3.366574
H	8.446032	-3.592533	3.618344
H	8.259936	-1.194292	2.888302
H	6.110305	-0.341350	1.925037
H	3.557056	-2.482306	4.336983
H	1.075420	-2.015496	5.326199
H	-2.676582	0.831059	7.226591
H	-3.195413	-1.444156	8.133024
H	-2.513574	-3.489930	6.829258
H	-1.359737	-3.222878	4.617773
H	-3.545043	-0.273294	3.290141
H	-4.393690	0.500135	0.819085
O	3.468380	3.946200	5.813657
O	3.096318	2.385186	4.167770
C	4.506632	2.941989	5.955277
C	3.858024	1.718532	5.210389
C	5.756827	3.486145	5.244710
C	4.790882	2.706607	7.438691
C	4.843488	0.754467	4.557616
C	2.834288	0.972544	6.083163
H	3.866274	2.459203	7.993625
H	5.230243	3.618445	7.891877
H	5.512800	1.873536	7.569193
H	6.014542	4.471654	5.681217
H	5.553277	3.644088	4.167180
H	6.627541	2.808266	5.358791
H	5.480516	1.271322	3.815132
H	4.286467	-0.041233	4.027646
H	5.497596	0.272640	5.313598
H	3.321815	0.341357	6.854607
H	2.143889	1.692160	6.569734
H	2.225503	0.326258	5.423213
C	4.940507	-1.351612	-0.694798
H	5.916613	-1.774112	-0.432121
C	-1.601382	4.674517	1.317823
C	-1.244581	3.385989	1.138379
C	-0.010902	2.890278	0.436209
C	1.201860	2.622100	1.346739
C	1.914930	3.840234	1.906272
C	1.665899	5.210745	1.665813
C	0.682238	5.891369	0.705740
C	-0.846403	5.925419	0.944001
H	-2.563065	4.866273	1.829154
H	-1.903521	2.603427	1.558074
H	2.956126	3.606714	2.175203
H	2.577264	5.824685	1.773686
H	-0.266307	1.941626	-0.064325
H	0.282349	3.575530	-0.382086
H	0.932982	1.920662	2.168386

H	1.962668	2.068312	0.744164
H	0.995509	6.955792	0.652081
H	0.887411	5.487337	-0.310806
H	-1.049732	6.672719	1.737760
H	-1.312788	6.366809	0.030495

A0

Zero-point correction= 1.216637 (Hartree/Particle)
Thermal correction to Energy= 1.326598
Thermal correction to Enthalpy= 1.327717
Thermal correction to Gibbs Free Energy= 1.066386

solvent: -5520.09152269

N	0.942925	2.083317	1.932144
C	1.656561	2.786774	0.978891
C	1.671184	2.154982	3.108631
C	2.896645	3.267467	1.554466
C	1.254891	1.643288	4.366461
C	0.110351	0.824269	4.556345
N	-0.695214	0.319615	3.550503
C	-1.707934	-0.383361	4.173647
C	-1.539046	-0.326019	5.614335
C	-0.406543	0.412072	5.851634
C	-2.802059	-1.000702	3.528213
C	-3.008863	-1.046263	2.128420
N	-2.261344	-0.380089	1.174974
C	-2.897079	-0.578174	-0.038985
C	-4.140811	-1.700515	1.500617
C	-4.068707	-1.417887	0.154689
C	-2.528840	0.007227	-1.276460
C	-1.469575	0.931981	-1.449566
N	-0.624168	1.384838	-0.452932
C	0.244626	2.283320	-1.042515
C	-1.114592	1.557295	-2.712371
C	-0.068831	2.413241	-2.455325
C	1.289016	2.971863	-0.376895
C	3.599639	4.618534	-2.949585
C	1.966551	5.356753	-0.785221
C	2.891546	3.635917	-2.237283
C	3.489566	5.972868	-2.585824
C	2.671672	6.337942	-1.501722
C	2.063923	3.992481	-1.148757
C	-4.920373	0.237644	-4.254772
C	-3.322936	-1.686058	-2.976715
C	-4.892474	-1.081664	-4.740836
C	-4.152340	0.591811	-3.133198
C	-3.342203	-0.363042	-2.477890
C	-4.091161	-2.042130	-4.097644
C	-4.020200	-1.267811	4.370382

C	-6.392096	-1.550860	5.878286
C	-4.328293	-2.494115	4.982481
C	-4.917956	-0.163719	4.521968
C	-6.094297	-0.314710	5.274604
C	-5.510951	-2.635520	5.733914
C	2.635574	0.991454	6.382680
C	3.133241	3.687554	6.974821
C	2.077929	1.999540	5.559865
C	3.424669	1.330254	7.493488
C	3.680204	2.681534	7.790922
C	2.326942	3.357655	5.873134
C	-4.487204	1.098063	3.858713
C	-4.612630	1.447075	2.516515
H	-5.197151	1.003742	1.705563
N	-3.497586	1.875687	4.420091
N	-2.993099	2.645645	3.501975
N	-3.662220	2.408410	2.310978
C	-3.236185	3.066011	1.135867
C	-2.306784	4.492540	-1.011934
C	-4.009574	3.073826	-0.033659
C	-1.603520	4.417939	0.192685
C	-3.524096	3.799566	-1.129207
N	-2.045847	3.701015	1.250394
Ir	-1.312485	4.066867	3.329918
Zn	-0.696941	0.916427	1.570227
B	0.171268	5.436733	3.049076
O	0.034015	6.641907	2.310884
O	1.441219	5.407415	3.631263
C	2.116626	6.676353	3.472396
C	2.604095	7.099983	4.864789
C	3.316709	6.480479	2.537166
C	0.973871	7.603859	2.864462
C	0.208932	8.387224	3.943307
C	1.431706	8.542158	1.747140
H	-4.172923	1.620860	-2.745604
H	-5.548636	0.995552	-4.749618
H	-5.494268	-1.361049	-5.620113
H	-4.059517	-3.076755	-4.474855
H	-2.691245	-2.435272	-2.474117
H	-4.967540	2.538869	-0.080424
H	-4.102698	3.839615	-2.064311
H	-1.893367	5.074826	-1.846703
H	-0.659135	4.952906	0.352232
H	1.777043	7.029557	5.595000
H	3.018022	8.129509	4.857106
H	3.406498	6.409288	5.196571
H	3.907551	7.413523	2.433879
H	3.975856	5.695079	2.956514
H	2.998810	6.148619	1.531965
H	0.843462	9.154786	4.430251
H	-0.199020	7.694256	4.704282

H	-0.652751	8.895711	3.469205
H	2.183560	9.267169	2.121525
H	1.875120	7.984216	0.901186
H	0.562352	9.113190	1.362328
H	-1.603604	1.365377	-3.673896
H	0.448629	3.070766	-3.162375
H	1.336555	5.646292	0.072843
H	2.581753	7.395998	-1.208206
H	4.044648	6.742902	-3.144704
H	4.247501	4.323470	-3.790421
H	2.982821	2.573646	-2.513247
H	3.667064	3.823483	1.010279
H	1.875028	4.148829	5.253242
H	3.324627	4.748733	7.199753
H	4.306714	2.948022	8.657237
H	3.854483	0.533968	8.122048
H	2.456384	-0.066246	6.132925
H	0.023331	0.689469	6.818153
H	-2.218130	-0.778694	6.345573
H	-6.773525	0.543931	5.392053
H	-7.315015	-1.663932	6.468521
H	-5.743674	-3.601268	6.210056
H	-3.633895	-3.340448	4.864378
H	-4.894368	-2.296982	2.028440
H	-4.754611	-1.738570	-0.637620
C	2.911885	2.865985	2.868261
H	3.691811	3.040456	3.616190
B	-1.103193	4.519861	5.324240
C	-0.718281	5.648107	7.323539
C	-1.573812	4.344582	7.593433
B	-2.547475	5.638364	3.345323
C	-4.097438	7.083939	2.450122
C	-3.826290	7.450358	3.959318
C	-3.598306	8.934934	4.236400
H	-4.504817	9.523592	3.984497
H	-3.372721	9.084815	5.311585
H	-2.744870	9.329866	3.655262
C	-4.894447	6.879522	4.907866
H	-4.537995	6.977082	5.952645
H	-5.859601	7.418201	4.817886
H	-5.059723	5.801894	4.707869
C	-3.256540	7.923392	1.473267
H	-3.328515	7.465268	0.465712
H	-3.610532	8.972503	1.409484
H	-2.188127	7.903817	1.771802
C	-5.568665	7.067929	2.039732
H	-6.027251	8.068736	2.180919
H	-5.655884	6.796782	0.967808
H	-6.144016	6.326509	2.626181
C	0.480809	5.824963	8.256831
H	1.041912	6.740578	7.978070

H	0.144267	5.935788	9.308881
H	1.174755	4.967940	8.194934
C	-1.561464	6.933198	7.305415
H	-1.943405	7.192374	8.313741
H	-0.931077	7.770839	6.944619
H	-2.402229	6.828836	6.595924
C	-2.831020	4.565398	8.438005
H	-3.364380	3.602251	8.570257
H	-2.571981	4.960961	9.442442
H	-3.525569	5.270724	7.943406
C	-0.739402	3.188719	8.170548
H	-0.451119	3.368041	9.226428
H	-1.345219	2.262312	8.120408
H	0.180785	3.032109	7.572397
O	-2.592536	6.725382	4.218007
O	-3.574565	5.729553	2.386677
O	-0.240915	5.427517	5.970207
O	-1.979454	3.955204	6.259856

A

Zero-point correction= 1.303784 (Hartree/Particle)
Thermal correction to Energy= 1.422115
Thermal correction to Enthalpy= 1.423234
Thermal correction to Gibbs Free Energy= 1.140394

solvent: -5768.50884856

N	2.156839	-0.004599	-0.502445
C	2.438037	0.608469	-1.702931
C	3.361183	-0.400214	0.036434
C	3.870453	0.536085	-1.971760
C	3.548342	-0.963138	1.326820
C	2.512486	-1.273113	2.244522
N	1.167746	-1.051403	2.041333
C	0.515687	-1.482979	3.176812
C	1.481841	-1.984550	4.143710
C	2.720877	-1.868507	3.558408
C	-0.884151	-1.470168	3.368461
C	-1.838038	-1.003993	2.428113
N	-1.559026	-0.288725	1.283150
C	-2.771850	0.071337	0.729017
C	-3.275023	-1.125473	2.598555
C	-3.857480	-0.462051	1.539594
C	-2.942205	0.850202	-0.447478
C	-1.884520	1.254400	-1.306722
N	-0.560632	0.882159	-1.182125
C	0.113048	1.424861	-2.258045
C	-2.045326	2.107087	-2.475863
C	-0.806679	2.211063	-3.066888
C	1.503178	1.290416	-2.523674

C	2.164286	2.261243	-6.164578
C	2.901197	3.101729	-3.589054
C	1.672192	1.582683	-5.036835
C	3.025700	3.361959	-6.008046
C	3.393243	3.780088	-4.716259
C	2.034210	1.993838	-3.734191
C	-6.431026	2.429537	-0.334190
C	-4.892849	0.831964	-2.054861
C	-6.969748	2.008916	-1.562794
C	-5.127753	2.052864	0.030511
C	-4.333191	1.251914	-0.824000
C	-6.195732	1.206605	-2.420537
C	-1.386403	-1.755492	4.754485
C	-2.216558	-2.142731	7.427705
C	-1.667735	-3.048671	5.231784
C	-1.532368	-0.641385	5.632458
C	-1.935521	-0.845107	6.965117
C	-2.086759	-3.241737	6.560964
C	5.533250	-0.364177	2.767749
C	7.094474	-2.369925	1.555836
C	4.960342	-1.189034	1.771522
C	6.880205	-0.532233	3.133973
C	7.663981	-1.531933	2.530777
C	5.752893	-2.196438	1.176653
C	-1.178350	0.694252	5.088949
C	-1.846808	1.432170	4.116815
H	-2.822623	1.292720	3.644224
N	0.054217	1.270125	5.305837
N	0.173406	2.298201	4.515124
N	-0.972825	2.422265	3.765516
C	-1.041433	3.418803	2.768679
C	-1.055272	5.415073	0.893414
C	-2.027920	3.383638	1.772630
C	-0.084382	5.347488	1.904210
C	-2.028388	4.402230	0.811379
N	-0.062671	4.352641	2.821580
Ir	1.646536	3.847198	4.187274
C	2.372212	3.994291	0.346659
C	2.189405	2.020993	2.279434
C	1.329415	3.045703	0.369639
C	3.362529	3.924356	1.326426
C	3.266778	2.937340	2.340951
N	1.250452	2.065860	1.286068
Zn	0.351843	0.195886	0.582157
B	3.008964	3.098675	5.499976
B	0.977329	4.912659	5.776802
B	2.832678	5.498360	3.938644
O	2.318533	6.743928	3.529918
O	4.224595	5.587426	4.100313
C	4.586043	6.991137	4.175633
C	4.603847	7.357947	5.669768

C	5.973524	7.180167	3.559601
C	3.406182	7.691068	3.378335
C	2.959425	9.035224	3.958743
C	3.682119	7.823986	1.871923
O	1.571648	6.010372	6.399109
O	-0.263374	4.618148	6.361429
C	0.832581	6.294233	7.614969
C	-0.593877	5.704891	7.269803
C	-1.358655	5.135873	8.464618
C	-1.470307	6.692443	6.481412
C	0.864168	7.803599	7.860815
C	1.531810	5.542289	8.758590
H	-4.715751	2.387065	0.995166
H	-7.027474	3.058967	0.345318
H	-7.991700	2.302938	-1.849777
H	-6.612800	0.862562	-3.380435
H	-4.290863	0.194807	-2.721224
H	-2.735421	2.546796	1.720472
H	-2.771569	4.381816	-0.000228
H	-1.024300	6.237898	0.164442
H	0.724643	6.087935	2.017796
H	-0.907647	7.109577	5.621756
H	-1.831383	7.528251	7.114852
H	-2.350792	6.148764	6.083194
H	-2.344333	4.750266	8.132910
H	-1.534446	5.920738	9.229696
H	-0.805348	4.299628	8.931729
H	1.065869	5.759013	9.741557
H	2.593692	5.858223	8.790015
H	1.525937	4.452073	8.567638
H	1.900857	8.121686	8.093711
H	0.530557	8.366273	6.968265
H	0.218538	8.078629	8.720795
H	2.138061	1.163752	2.965254
H	0.536559	3.054838	-0.393324
H	2.388329	4.765656	-0.437435
H	4.210228	4.624858	1.340882
H	3.606852	7.177802	6.118346
H	4.913290	8.409371	5.840133
H	5.324468	6.690882	6.184127
H	6.247368	8.255257	3.523301
H	6.729315	6.653327	4.176576
H	6.027439	6.765989	2.534371
H	3.796414	9.764248	3.968430
H	2.578814	8.909186	4.989626
H	2.139761	9.458327	3.342429
H	4.464371	8.578776	1.652659
H	3.997293	6.852819	1.441881
H	2.747201	8.133512	1.361321
H	-2.982847	2.570709	-2.803281
H	-0.539299	2.774484	-3.967925

H	3.183064	3.423787	-2.573917
H	4.065702	4.642990	-4.585189
H	3.411585	3.893051	-6.892645
H	1.876953	1.924223	-7.173392
H	1.000879	0.717711	-5.155688
H	4.369326	0.912008	-2.871988
H	5.303458	-2.843572	0.406935
H	7.698022	-3.162800	1.085720
H	8.718808	-1.658482	2.822238
H	7.321560	0.129350	3.894924
H	4.920495	0.414250	3.252997
H	3.693044	-2.163422	3.969536
H	1.235847	-2.381131	5.135451
H	-2.025127	0.022411	7.637151
H	-2.535303	-2.295925	8.470719
H	-2.305739	-4.258772	6.923183
H	-1.545941	-3.905910	4.551524
H	-3.769348	-1.663346	3.416518
H	-4.925162	-0.368206	1.310048
O	3.085419	3.390040	6.866650
O	3.920013	2.061541	5.180387
C	4.220464	2.686040	7.425543
C	4.356497	1.467614	6.438400
C	5.419102	3.648152	7.354591
C	3.907133	2.308088	8.873050
C	5.777714	0.928803	6.290041
C	3.372960	0.328130	6.751526
H	2.943446	1.769155	8.945026
H	3.831883	3.224675	9.492806
H	4.708645	1.668136	9.297816
H	5.151627	4.582077	7.888720
H	5.636194	3.916238	6.300884
H	6.329744	3.221406	7.822556
H	6.445498	1.678704	5.823692
H	5.776815	0.019815	5.657060
H	6.192806	0.650223	7.281158
H	3.667779	-0.238080	7.658910
H	2.342546	0.717734	6.874750
H	3.354762	-0.367139	5.888770
H	4.076168	2.784971	3.066835
C	4.445548	-0.072740	-0.883798
H	5.508102	-0.278155	-0.713193

A’

Zero-point correction= 1.303730 (Hartree/Particle)
Thermal correction to Energy= 1.422169
Thermal correction to Enthalpy= 1.423288
Thermal correction to Gibbs Free Energy= 1.139777

solvent: -5768.50761486

N	1.898955	1.488289	0.636005
C	2.132176	2.449605	-0.323395
C	3.129089	1.095502	1.123762
C	3.564555	2.655047	-0.471005
C	3.344667	0.143580	2.161527
C	2.319281	-0.605820	2.805130
N	0.985912	-0.631497	2.446803
C	0.341547	-1.431250	3.367158
C	1.298541	-1.934031	4.341512
C	2.525886	-1.433288	3.986627
C	-1.051257	-1.676989	3.416184
C	-2.010578	-1.175734	2.497770
N	-1.761513	-0.282817	1.477275
C	-2.985626	0.072550	0.946051
C	-3.439201	-1.419057	2.604536
C	-4.046363	-0.646793	1.638703
C	-3.205540	1.058666	-0.051216
C	-2.202518	1.876423	-0.634556
N	-0.855426	1.839794	-0.336741
C	-0.242803	2.804209	-1.110260
C	-2.453245	2.900555	-1.636689
C	-1.241160	3.487565	-1.919191
C	1.146230	3.106331	-1.106710
C	1.888092	5.346393	-4.124809
C	2.138516	5.403559	-1.328052
C	1.486066	4.238017	-3.360494
C	2.409772	6.489952	-3.492985
C	2.536090	6.512882	-2.092356
C	1.599946	4.253678	-1.951558
C	-6.625861	2.686328	-0.593634
C	-5.322540	0.271803	-1.190756
C	-7.311023	1.677522	-1.294151
C	-5.294215	2.486188	-0.193831
C	-4.621359	1.277962	-0.487108
C	-6.654762	0.469345	-1.590587
C	-1.601513	-2.147255	4.736453
C	-2.646705	-2.804869	7.278296
C	-1.900541	-3.482065	5.056827
C	-1.834241	-1.125243	5.711107
C	-2.349697	-1.463297	6.973217
C	-2.422563	-3.810275	6.322951
C	5.567704	0.940339	3.076594
C	6.636280	-1.651859	2.870573
C	4.754704	-0.107156	2.587082
C	6.900184	0.696007	3.438805
C	7.441139	-0.598952	3.340198
C	5.305099	-1.406421	2.492841
C	-1.444467	0.248564	5.288841
C	-2.098418	1.077889	4.380293
H	-3.105078	1.033485	3.954948
N	-0.133878	0.663362	5.391705

N	0.053580	1.658840	4.572782
N	-1.136994	1.941965	3.935595
C	-1.155034	2.942373	2.937801
C	-0.990574	4.945735	1.072178
C	-2.351031	3.442455	2.400619
C	0.147081	4.377307	1.655033
C	-2.257047	4.459613	1.440907
N	0.070837	3.378738	2.562136
Ir	1.792529	2.726999	3.857016
Zn	0.114849	0.455659	0.899107
B	3.048875	2.168828	5.347468
B	1.435138	4.360042	4.941882
B	3.350361	3.851679	3.148372
O	3.325267	4.695661	2.016576
O	4.578807	3.999539	3.805368
C	5.353056	5.082176	3.257767
C	5.558668	6.099522	4.390131
C	6.706824	4.495641	2.828085
C	4.443504	5.622929	2.049068
C	3.832391	7.011608	2.294880
C	5.161442	5.597407	0.694955
O	2.162549	5.554566	4.993106
O	0.246129	4.465672	5.680844
C	1.577660	6.375644	6.040292
C	0.084176	5.861549	6.047209
C	-0.615380	5.943251	7.402891
C	-0.773465	6.508115	4.944811
C	1.748052	7.848092	5.666738
C	2.332363	6.046707	7.338323
H	-4.756339	3.270735	0.359609
H	-7.133691	3.633670	-0.351590
H	-8.355472	1.832497	-1.607978
H	-7.183171	-0.324748	-2.142051
H	-4.805032	-0.671983	-1.424141
H	-3.323415	3.042413	2.718292
H	-3.167832	4.873107	0.983486
H	-0.876391	5.736498	0.317773
H	1.172120	4.702217	1.420332
H	-0.251173	6.461912	3.967111
H	-1.022707	7.565241	5.169860
H	-1.718492	5.936012	4.848828
H	-1.655275	5.567607	7.314968
H	-0.655556	6.992500	7.763163
H	-0.093175	5.325376	8.157390
H	1.984036	6.665952	8.190069
H	3.411551	6.241232	7.176338
H	2.231749	4.970810	7.582421
H	2.823975	8.116903	5.678262
H	1.357317	8.060399	4.653426
H	1.223596	8.500804	6.395412
H	4.581060	6.391129	4.816307

H	6.104353	7.001229	4.044421
H	6.147472	5.623268	5.199722
H	7.386061	5.268102	2.414372
H	7.193771	4.048229	3.718344
H	6.580696	3.691539	2.077801
H	4.604115	7.804901	2.361111
H	3.228631	7.002449	3.221555
H	3.156409	7.259250	1.449640
H	6.037115	6.278108	0.701662
H	5.506814	4.579356	0.439752
H	4.479257	5.930539	-0.109841
H	-3.430940	3.141721	-2.068773
H	-1.044983	4.315840	-2.608638
H	2.261289	5.404953	-0.233535
H	2.950329	7.400578	-1.587575
H	2.722718	7.359514	-4.092474
H	1.798238	5.314967	-5.222497
H	1.080482	3.340576	-3.853901
H	4.032139	3.351400	-1.175003
H	4.678018	-2.227283	2.109958
H	7.049043	-2.670126	2.787589
H	8.485869	-0.789040	3.634137
H	7.513251	1.526680	3.823180
H	5.134779	1.940302	3.213710
H	3.481405	-1.544598	4.505808
H	1.047431	-2.562959	5.203227
H	-2.513066	-0.669541	7.718584
H	-3.050823	-3.064903	8.269297
H	-2.652410	-4.860352	6.564186
H	-1.718683	-4.266206	4.305376
H	-3.913171	-2.084025	3.336375
H	-5.117782	-0.557175	1.425717
O	3.279816	2.881198	6.538348
O	3.713995	0.932913	5.395703
C	4.333222	2.203294	7.268346
C	4.173114	0.720427	6.755639
C	5.664856	2.838922	6.832969
C	4.101117	2.397561	8.766783
C	5.464685	-0.095916	6.717823
C	3.061391	-0.046380	7.492347
H	3.073501	2.104373	9.054050
H	4.236618	3.466161	9.032093
H	4.824135	1.799726	9.360520
H	5.612652	3.929367	7.025320
H	5.815178	2.704827	5.743656
H	6.529390	2.418805	7.386828
H	6.208124	0.358098	6.037632
H	5.255531	-1.118220	6.340741
H	5.905020	-0.186564	7.732974
H	3.352989	-0.306343	8.530826
H	2.123702	0.544095	7.507407

H	2.846818	-0.982747	6.940286
C	4.183490	1.808820	0.416210
H	5.258147	1.674228	0.577995
N	0.501155	-1.020342	-0.626011
C	0.970374	-0.665437	-1.837457
C	0.301999	-2.322384	-0.345576
C	1.263201	-1.612013	-2.831041
H	1.112674	0.416513	-1.996614
C	0.566051	-3.335861	-1.279149
H	-0.078524	-2.538033	0.666989
C	1.056620	-2.972399	-2.545203
H	1.648403	-1.284106	-3.808182
H	0.391531	-4.389375	-1.013627
H	1.277433	-3.742404	-3.301476

A + COD

Zero-point correction= 1.481234 (Hartree/Particle)
Thermal correction to Energy= 1.611757
Thermal correction to Enthalpy= 1.612876
Thermal correction to Gibbs Free Energy= 1.304128

solvent: -6080.67784433

N	2.707104	-0.814351	-1.482754
C	3.117821	-1.011969	-2.783168
C	3.818066	-0.941660	-0.680620
C	4.540413	-1.331168	-2.800534
C	3.836787	-0.808753	0.735566
C	2.684813	-0.654223	1.553110
N	1.394663	-0.461786	1.099285
C	0.575571	-0.482696	2.212456
C	1.371753	-0.671610	3.412825
C	2.680896	-0.751424	3.007188
C	-0.833370	-0.361366	2.208835
C	-1.623742	-0.109578	1.058720
N	-1.158291	0.002525	-0.236613
C	-2.256705	0.231717	-1.038881
C	-3.066096	0.067889	1.080488
C	-3.458560	0.299087	-0.217856
C	-2.253173	0.279354	-2.459744
C	-1.136471	-0.033666	-3.285010
N	0.107048	-0.426201	-2.834373
C	0.911173	-0.576196	-3.943537
C	-1.120921	0.068163	-4.738156
C	0.146060	-0.277581	-5.146810
C	2.298319	-0.894718	-3.937650
C	3.192512	-2.297402	-7.378620
C	3.964193	-0.178799	-5.705630
C	2.579960	-2.132739	-6.125256
C	4.193244	-1.403339	-7.799737

C	4.576651	-0.342967	-6.959004
C	2.958066	-1.072103	-5.269665
C	-5.293439	2.337610	-3.503314
C	-4.201321	-0.199426	-4.013059
C	-5.949403	1.458268	-4.383544
C	-4.095016	1.950292	-2.881801
C	-3.528786	0.677063	-3.129551
C	-5.399633	0.188116	-4.634850
C	-1.581430	-0.775924	3.451870
C	-3.151830	-1.802810	5.591520
C	-1.982604	-2.132148	3.491658
C	-1.967095	0.071342	4.526086
C	-2.754327	-0.459624	5.578592
C	-2.755210	-2.648524	4.541955
C	6.093374	0.220629	1.141803
C	6.791483	-1.780100	2.977869
C	5.175055	-0.822318	1.405833
C	7.337255	0.269258	1.790599
C	7.686984	-0.726497	2.719493
C	5.550293	-1.831737	2.321701
C	-1.606953	1.501065	4.611550
C	-2.457756	2.573168	4.869705
H	-3.544129	2.629023	4.970476
N	-0.318025	1.974755	4.535827
N	-0.335894	3.268821	4.718581
N	-1.637921	3.669224	4.927750
C	-1.915221	5.035024	5.102506
C	-2.285383	7.720448	5.501352
C	-3.183933	5.478781	5.514522
C	-1.058439	7.190848	5.097392
C	-3.374738	6.850433	5.707540
N	-0.863250	5.864208	4.880635
Ir	1.113600	4.869850	4.801547
Zn	0.831367	-0.115248	-0.889760
B	2.758774	3.734962	5.312009
B	1.043246	5.042832	6.856923
B	2.295754	6.524630	5.073523
O	1.901105	7.649106	5.845983
O	3.568424	6.766852	4.538421
C	4.178671	7.826255	5.313909
C	4.864037	7.148914	6.515786
C	5.198060	8.563700	4.446750
C	2.920245	8.676736	5.734025
C	3.048218	9.396767	7.076231
C	2.463810	9.651129	4.635787
O	2.093164	5.224393	7.763727
O	-0.191156	5.114402	7.525248
C	1.541308	5.313639	9.106189
C	0.042807	5.725154	8.822600
C	-0.981261	5.180508	9.817936
C	-0.133281	7.241491	8.632029

C	2.350767	6.345278	9.892711
C	1.662099	3.927090	9.754591
H	-3.578003	2.636965	-2.192571
H	-5.715941	3.335085	-3.301898
H	-6.889479	1.761328	-4.871351
H	-5.912422	-0.510560	-5.315172
H	-3.776207	-1.198181	-4.198755
H	-3.995042	4.760998	5.698452
H	-4.354119	7.233383	6.031747
H	-2.381436	8.804717	5.657972
H	-0.163264	7.816549	4.970001
H	0.597998	7.623285	7.890679
H	-0.024162	7.797155	9.585731
H	-1.149465	7.429935	8.229544
H	-1.996580	5.524043	9.532555
H	-0.769313	5.547387	10.843861
H	-0.987197	4.074109	9.828766
H	1.275274	3.923589	10.793865
H	2.728853	3.634802	9.781436
H	1.117083	3.168573	9.158217
H	3.390668	5.983808	10.024985
H	2.392516	7.312296	9.357778
H	1.910447	6.511865	10.897883
H	4.112997	6.631597	7.148024
H	5.438299	7.870048	7.133208
H	5.554704	6.374900	6.126643
H	5.602455	9.449524	4.979933
H	6.043890	7.887371	4.209312
H	4.751925	8.896542	3.489942
H	3.888705	10.121567	7.057587
H	3.220926	8.676137	7.897179
H	2.115603	9.953834	7.301325
H	3.155816	10.510713	4.524082
H	2.385093	9.126640	3.661833
H	1.461021	10.046485	4.898504
H	-1.962408	0.384214	-5.364852
H	0.535872	-0.301027	-6.170770
H	4.255984	0.655417	-5.048442
H	5.354941	0.366001	-7.284039
H	4.673551	-1.532446	-8.782690
H	2.889321	-3.133367	-8.029118
H	1.799132	-2.833701	-5.790767
H	5.124806	-1.585248	-3.692081
H	4.856604	-2.665024	2.512945
H	7.064139	-2.573635	3.691789
H	8.658036	-0.684881	3.237672
H	8.030560	1.099342	1.581934
H	5.808914	1.011043	0.429560
H	3.565922	-0.880687	3.636043
H	0.977933	-0.695080	4.434708
H	-3.032821	0.205522	6.411443

H	-3.757996	-2.190327	6.425352
H	-3.049426	-3.709904	4.537200
H	-1.683362	-2.783863	2.655897
H	-3.693905	0.014780	1.977898
H	-4.475218	0.457881	-0.594761
O	4.085178	3.932556	4.896879
O	2.665013	2.561948	6.075876
C	4.824062	2.711687	5.144214
C	4.008770	2.074586	6.334846
C	4.755414	1.870391	3.859656
C	6.274754	3.070588	5.468390
C	3.983804	0.546165	6.350872
C	4.440748	2.622094	7.703153
H	6.332799	3.811267	6.288423
H	6.756339	3.515998	4.574170
H	6.851019	2.166668	5.756876
H	5.156919	2.466146	3.015607
H	3.707574	1.601683	3.619254
H	5.352816	0.938918	3.927838
H	3.468725	0.155657	5.455348
H	3.430612	0.184867	7.241464
H	5.012414	0.130503	6.387161
H	5.454955	2.274283	7.987086
H	4.409840	3.729898	7.704937
H	3.723680	2.263716	8.466750
C	4.977534	-1.273259	-1.497589
H	5.987074	-1.466440	-1.116991
C	-1.607439	4.723104	1.954256
C	-1.200023	3.454465	1.740408
C	0.090585	2.995749	1.113505
C	1.309302	2.840323	2.042122
C	1.961251	4.093550	2.562975
C	1.636786	5.430975	2.348015
C	0.615314	6.040200	1.387606
C	-0.897176	6.024903	1.689254
H	-2.613592	4.855600	2.395628
H	-1.889817	2.645380	2.041838
H	2.984055	3.935289	2.939387
H	2.454173	6.125341	2.600640
H	-0.092492	2.008149	0.652933
H	0.360184	3.669930	0.280290
H	1.059695	2.163221	2.887219
H	2.093969	2.279536	1.488017
H	0.890870	7.110356	1.273801
H	0.790021	5.593804	0.383408
H	-1.082421	6.687611	2.560160
H	-1.412173	6.536403	0.840115
C	2.621638	2.386125	-0.968620
C	3.019949	3.716762	-1.166171
C	2.180136	4.565826	-1.905836
C	0.979997	4.049929	-2.425001

C	0.650575	2.713367	-2.156452
N	1.452505	1.905167	-1.434888
H	2.458279	5.617793	-2.075548
H	3.244362	1.668779	-0.410065
H	3.966267	4.077081	-0.736885
H	0.295222	4.675740	-3.016300
H	-0.290782	2.263459	-2.510974

A’ + COD

Zero-point correction= 1.479864 (Hartree/Particle)
Thermal correction to Energy= 1.611048
Thermal correction to Enthalpy= 1.612166
Thermal correction to Gibbs Free Energy= 1.297606

solvent: -6080.68463311

N	2.643877	-0.649871	-0.578337
C	3.066049	-0.389684	-1.866131
C	3.721814	-1.180284	0.101116
C	4.462136	-0.777257	-2.011454
C	3.726572	-1.619703	1.452946
C	2.607082	-1.610328	2.326364
N	1.338785	-1.183084	2.000289
C	0.574543	-1.276472	3.141276
C	1.384230	-1.788769	4.236237
C	2.644365	-2.002189	3.730415
C	-0.790874	-0.917158	3.249790
C	-1.607660	-0.442011	2.192347
N	-1.203532	-0.240412	0.890492
C	-2.299338	0.224216	0.193159
C	-3.021005	-0.118108	2.322198
C	-3.451019	0.292816	1.081687
C	-2.308691	0.630499	-1.165861
C	-1.193052	0.610592	-2.042705
N	0.068049	0.140985	-1.738232
C	0.870193	0.402937	-2.828037
C	-1.192887	1.184998	-3.379890
C	0.086337	1.056838	-3.867708
C	2.261368	0.127047	-2.918565
C	3.177312	-0.002980	-6.627801
C	3.971343	1.369517	-4.309987
C	2.546553	-0.269773	-5.401511
C	4.208269	0.951040	-6.699194
C	4.602528	1.636476	-5.535942
C	2.934233	0.411256	-4.223788
C	-5.185392	3.072163	-1.722116
C	-4.354445	0.558959	-2.661645
C	-5.956793	2.394633	-2.684032
C	-4.003172	2.495751	-1.228537
C	-3.577301	1.229104	-1.691370

C	-5.537249	1.137085	-3.153787
C	-1.432729	-1.049216	4.595788
C	-2.748520	-1.351082	7.089760
C	-1.723545	-2.328577	5.115711
C	-1.796943	0.098119	5.353704
C	-2.456361	-0.072193	6.593456
C	-2.373828	-2.486287	6.350029
C	6.126500	-1.233620	2.141691
C	6.388875	-3.898775	2.984555
C	5.024829	-2.110843	2.012164
C	7.340194	-1.680954	2.688067
C	7.475482	-3.014956	3.112615
C	5.174470	-3.450461	2.438848
C	-1.506311	1.466967	4.886632
C	-2.341662	2.576890	4.951383
H	-3.377346	2.686805	5.279835
N	-0.288381	1.861000	4.377756
N	-0.327771	3.144085	4.149997
N	-1.585834	3.617012	4.475778
C	-1.842420	4.994525	4.426944
C	-2.144541	7.720310	4.496735
C	-3.097017	5.519957	4.791571
C	-0.938366	7.118312	4.134882
C	-3.253248	6.908648	4.816476
N	-0.778589	5.766963	4.076170
Ir	1.177299	4.707301	3.968071
Zn	0.663091	-0.711216	0.082786
B	2.744390	3.619854	4.746836
B	0.980717	4.880644	6.038899
B	2.447381	6.303553	4.107473
O	2.017067	7.646843	4.164907
O	3.850043	6.266083	4.095973
C	4.329196	7.553908	4.555113
C	4.412658	7.470880	6.089728
C	5.703389	7.824914	3.944850
C	3.177146	8.514775	4.068746
C	2.948187	9.743794	4.949062
C	3.321842	8.921252	2.593122
O	1.144222	6.035889	6.801632
O	0.454151	3.840614	6.818524
C	0.959505	5.689683	8.203922
C	0.076689	4.378807	8.109805
C	0.365635	3.324281	9.178582
C	-1.431277	4.674383	8.033770
C	0.286407	6.866372	8.911716
C	2.355739	5.428784	8.786510
H	-3.382432	3.027232	-0.488400
H	-5.502086	4.062785	-1.357923
H	-6.883439	2.848220	-3.070666
H	-6.136332	0.600840	-3.907239
H	-4.020965	-0.425688	-3.025911

H	-3.927157	4.851283	5.056301
H	-4.221050	7.351978	5.095403
H	-2.213379	8.817642	4.521938
H	-0.027008	7.694283	3.906723
H	-1.640217	5.447995	7.267490
H	-1.845560	5.013824	9.004859
H	-1.955957	3.745353	7.728759
H	-0.288949	2.443034	9.019656
H	0.174269	3.723129	10.196577
H	1.414875	2.978219	9.120987
H	2.316463	5.169131	9.863863
H	2.970248	6.343524	8.669942
H	2.855890	4.621334	8.219954
H	0.974558	7.735668	8.925744
H	-0.639538	7.178465	8.392338
H	0.038890	6.605283	9.961871
H	3.406414	7.281347	6.514786
H	4.842905	8.390194	6.537499
H	5.049533	6.606144	6.360833
H	6.053515	8.847854	4.196664
H	6.438346	7.098739	4.347760
H	5.687926	7.713281	2.843804
H	3.864801	10.367853	5.001749
H	2.655704	9.449121	5.974287
H	2.132652	10.366835	4.527203
H	4.144218	9.649605	2.439658
H	3.512388	8.031366	1.959815
H	2.374073	9.386119	2.253377
H	-2.055389	1.654106	-3.866885
H	0.474765	1.405578	-4.831198
H	4.272214	1.911331	-3.399633
H	5.404069	2.390838	-5.584261
H	4.703184	1.161055	-7.660695
H	2.864724	-0.547449	-7.533261
H	1.741422	-1.019223	-5.342406
H	5.049370	-0.705869	-2.933852
H	4.322844	-4.141024	2.334281
H	6.488611	-4.947224	3.308400
H	8.426968	-3.365684	3.543020
H	8.183307	-0.979370	2.790893
H	6.012367	-0.186693	1.821823
H	3.527959	-2.359625	4.270283
H	1.038919	-1.940897	5.264785
H	-2.709562	0.821514	7.185943
H	-3.254931	-1.461474	8.061515
H	-2.591526	-3.496133	6.732203
H	-1.437595	-3.211077	4.522000
H	-3.607512	-0.202013	3.244398
H	-4.456896	0.614299	0.788988
O	3.470370	3.934619	5.913501
O	3.139122	2.363620	4.269148

C	4.513368	2.939920	6.078243
C	3.889018	1.707923	5.325918
C	5.772424	3.491220	5.388862
C	4.771740	2.713462	7.567920
C	4.893574	0.750074	4.692256
C	2.858768	0.954137	6.184333
H	3.838631	2.462323	8.106831
H	5.195653	3.630940	8.024567
H	5.497284	1.886456	7.716200
H	6.014491	4.480181	5.826516
H	5.586915	3.644761	4.307477
H	6.646007	2.820243	5.520936
H	5.543934	1.269798	3.963593
H	4.351794	-0.046436	4.147398
H	5.533795	0.270350	5.461527
H	3.339851	0.332958	6.967971
H	2.152362	1.668537	6.655408
H	2.269338	0.298211	5.516406
C	4.867609	-1.268411	-0.792914
H	5.850721	-1.673376	-0.527959
C	-1.492836	4.613329	1.290447
C	-1.137622	3.320074	1.141951
C	0.108105	2.800163	0.479268
C	1.302888	2.552914	1.418223
C	2.007597	3.780703	1.961433
C	1.768339	5.145706	1.693988
C	0.805812	5.808466	0.700791
C	-0.725684	5.856003	0.913973
H	-2.465395	4.817309	1.776370
H	-1.812897	2.551355	1.560895
H	3.039169	3.550933	2.268171
H	2.674862	5.763435	1.819604
H	-0.137749	1.835739	0.005383
H	0.417643	3.460385	-0.353968
H	1.018048	1.871333	2.249821
H	2.066747	1.975611	0.845945
H	1.126488	6.869436	0.625076
H	1.023519	5.376311	-0.301230
H	-0.936143	6.614157	1.695831
H	-1.174187	6.289008	-0.012528
N	0.289365	-2.759832	-0.463825
C	-0.389851	-3.566952	0.373358
C	0.753344	-3.248418	-1.629754
C	-0.636562	-4.915049	0.072974
H	-0.736011	-3.098528	1.310273
C	0.555377	-4.584561	-2.009716
H	1.301981	-2.531064	-2.263127
C	-0.153420	-5.432436	-1.141367
H	-1.196580	-5.544549	0.780780
H	0.952644	-4.949597	-2.968741
H	-0.327088	-6.487091	-1.408154

TS_{AB}*^{meta}*

Zero-point correction= 1.299949 (Hartree/Particle)
Thermal correction to Energy= 1.417245
Thermal correction to Enthalpy= 1.418364
Thermal correction to Gibbs Free Energy= 1.140263

solvent: -5768.45727433

N	3.220420	-0.425898	-0.653512
C	3.605548	0.051172	-1.889523
C	4.265877	-1.171023	-0.156064
C	4.929200	-0.464556	-2.214413
C	4.295173	-1.829194	1.105400
C	3.183540	-1.983907	1.977812
N	1.901026	-1.553166	1.718303
C	1.110565	-1.967008	2.767854
C	1.932612	-2.654481	3.755096
C	3.219612	-2.659941	3.268087
C	-0.292344	-1.759108	2.871640
C	-1.063094	-0.984783	1.963607
N	-0.540049	-0.182099	0.974324
C	-1.555885	0.630657	0.532685
C	-2.497909	-0.761016	2.060770
C	-2.797183	0.274488	1.203915
C	-1.426021	1.686348	-0.411835
C	-0.340532	1.835451	-1.315233
N	0.750501	0.995766	-1.373428
C	1.566509	1.462624	-2.374262
C	-0.209034	2.884269	-2.318675
C	0.961621	2.635972	-2.997246
C	2.862797	0.962527	-2.690767
C	3.498449	1.855316	-6.348906
C	4.739381	2.206979	-3.855345
C	2.901065	1.338458	-5.187757
C	4.719155	2.549328	-6.267825
C	5.336623	2.724495	-5.016151
C	3.513667	1.502388	-3.922443
C	-3.660270	4.412144	1.006024
C	-3.311934	3.071478	-1.441259
C	-4.478044	4.735946	-0.092481
C	-2.673592	3.422048	0.881767
C	-2.484479	2.736345	-0.345853
C	-4.300261	4.063155	-1.314995
C	-0.997664	-2.338475	4.056677
C	-2.365918	-3.527521	6.252670
C	-0.997077	-3.738585	4.255133
C	-1.702293	-1.526086	4.998728
C	-2.378948	-2.135994	6.079515
C	-1.667832	-4.331967	5.335380

C	6.724180	-1.582459	1.742565
C	6.981813	-4.349444	2.140759
C	5.601434	-2.417643	1.533420
C	7.956137	-2.121751	2.147767
C	8.089088	-3.507411	2.349344
C	5.749697	-3.809719	1.736147
C	-1.773754	-0.058912	4.858136
C	-2.881362	0.771165	5.022083
H	-3.919084	0.553726	5.284299
N	-0.703420	0.715308	4.474532
N	-1.087272	1.953467	4.400781
N	-2.431009	2.029523	4.711474
C	-3.062328	3.278748	4.669050
C	-4.164514	5.795088	4.597121
C	-4.459246	3.402941	4.777770
C	-2.788020	5.585053	4.478633
C	-5.017118	4.684197	4.743551
N	-2.235283	4.347385	4.507623
Ir	-0.085974	3.893124	4.463681
C	3.297025	3.736660	1.516173
C	1.087029	2.263149	2.168668
C	3.042575	2.493736	0.921251
C	1.268914	3.483169	2.851964
C	2.412090	4.233392	2.486506
N	1.951732	1.778866	1.257942
Zn	1.443575	-0.040219	0.339612
B	1.535607	2.918394	5.428557
B	-0.319748	4.019696	6.594428
B	0.652052	5.775108	4.862930
O	-0.186489	6.828781	5.290844
O	1.963244	6.230378	4.758813
C	2.061640	7.543905	5.373347
C	2.520442	7.320186	6.822551
C	3.090001	8.372355	4.602250
C	0.573953	8.068339	5.267756
C	0.117107	8.944624	6.433796
C	0.271398	8.752471	3.924775
O	-1.539534	3.625926	7.156952
O	0.466753	4.678690	7.535602
C	-1.714625	4.395430	8.381194
C	-0.220506	4.629535	8.823371
C	0.030141	5.943317	9.562700
C	0.357077	3.444523	9.605925
C	-2.565206	3.587989	9.359041
C	-2.422873	5.700135	7.976705
H	-2.018839	3.181044	1.735726
H	-3.785770	4.937434	1.965461
H	-5.253469	5.512800	0.003573
H	-4.942768	4.305960	-2.176619
H	-3.185183	2.530497	-2.392056
H	-5.090099	2.509507	4.876275

H	-6.106857	4.813354	4.825217
H	-4.557897	6.821280	4.572315
H	-2.062943	6.408885	4.404167
H	-0.079979	3.372030	10.622383
H	1.451693	3.578126	9.704031
H	0.178053	2.497647	9.059959
H	1.102180	6.027831	9.831468
H	-0.233261	6.815715	8.935881
H	-0.565090	5.985184	10.498461
H	-2.664352	6.332572	8.854948
H	-3.369469	5.444230	7.458661
H	-1.794882	6.280147	7.268509
H	-2.153600	2.572051	9.507990
H	-3.595783	3.484657	8.962314
H	-2.625258	4.097993	10.342844
H	0.225149	1.613088	2.367220
H	3.700493	2.039507	0.162627
H	4.194001	4.309166	1.232207
H	2.617116	5.184724	2.996508
H	3.482222	6.770073	6.805385
H	2.669867	8.276790	7.363487
H	1.789077	6.690285	7.364041
H	3.121088	9.414455	4.983031
H	4.097379	7.928181	4.731813
H	2.867679	8.395645	3.518544
H	0.739269	9.860448	6.507332
H	0.179706	8.396972	7.392724
H	-0.937369	9.254872	6.284582
H	0.766523	9.740842	3.839558
H	-0.823556	8.905815	3.835486
H	0.597615	8.115832	3.077774
H	-0.912066	3.709793	-2.478292
H	1.399549	3.220180	-3.814815
H	5.213737	2.356796	-2.872866
H	6.287440	3.276293	-4.941657
H	5.187640	2.955289	-7.178540
H	3.009231	1.710587	-7.325513
H	1.947702	0.790569	-5.249004
H	5.465437	-0.293933	-3.154769
H	4.884232	-4.467891	1.561442
H	7.079986	-5.437131	2.287094
H	9.055317	-3.931127	2.666469
H	8.817391	-1.454372	2.311856
H	6.613871	-0.497460	1.589157
H	4.113508	-3.074986	3.747074
H	1.579582	-3.065097	4.707653
H	-2.896824	-1.495321	6.811273
H	-2.892227	-3.981494	7.106865
H	-1.651198	-5.426666	5.456202
H	-0.466020	-4.365725	3.522600
H	-3.185984	-1.318790	2.706374

H	-3.775141	0.729116	1.006123
O	1.280146	1.845357	6.284102
O	2.886440	3.019421	5.141093
C	2.539803	1.176311	6.588121
C	3.488257	1.724945	5.447127
C	2.289449	-0.331653	6.540988
C	2.974922	1.619507	7.989750
C	4.941996	1.953737	5.858055
C	3.419615	0.872165	4.170064
H	3.105932	2.719206	8.028503
H	2.192378	1.338911	8.720970
H	3.922204	1.130333	8.294135
H	3.240461	-0.896651	6.627828
H	1.628340	-0.629218	7.380250
H	1.790945	-0.619659	5.597098
H	5.024034	2.700145	6.670702
H	5.409519	1.004131	6.191207
H	5.514692	2.333100	4.987845
H	3.889279	1.429172	3.336522
H	2.372792	0.653670	3.882045
H	3.947345	-0.094615	4.288176
H	-0.183153	4.407467	2.886217
C	5.344059	-1.209475	-1.134335
H	6.284492	-1.760958	-1.020457

TS_{AB}^{para}

Zero-point correction= 1.298385 (Hartree/Particle)
Thermal correction to Energy= 1.416940
Thermal correction to Enthalpy= 1.418058
Thermal correction to Gibbs Free Energy= 1.132034

solvent: -5768.46983856

N	2.773412	-0.161660	-0.980962
C	3.240244	0.207791	-2.223403
C	3.861696	-0.579277	-0.246641
C	4.679005	-0.018242	-2.291741
C	3.828555	-1.053240	1.092938
C	2.649817	-1.288789	1.848068
N	1.363039	-1.042254	1.419787
C	0.527568	-1.385923	2.458752
C	1.306701	-1.881920	3.584212
C	2.626240	-1.808646	3.210953
C	-0.880578	-1.250019	2.472610
C	-1.669226	-0.683482	1.439545
N	-1.191979	-0.173547	0.252912
C	-2.281378	0.266869	-0.466147
C	-3.120637	-0.575526	1.473533
C	-3.502087	0.017978	0.291626
C	-2.236668	0.845395	-1.763750

C	-1.066047	0.993363	-2.558174
N	0.203569	0.589055	-2.205363
C	1.042074	0.930691	-3.243812
C	-1.030868	1.626228	-3.870349
C	0.274926	1.581860	-4.298037
C	2.450006	0.731032	-3.282397
C	3.565676	0.834120	-6.937135
C	4.106971	2.180107	-4.533168
C	2.900493	0.461062	-5.757473
C	4.504257	1.881441	-6.918859
C	4.772429	2.553237	-5.712637
C	3.161744	1.128084	-4.538064
C	-5.446419	2.859887	-2.258906
C	-4.082081	0.740983	-3.497475
C	-5.983915	2.263155	-3.413542
C	-4.230942	2.398647	-1.726999
C	-3.529600	1.332978	-2.337944
C	-5.297178	1.202054	-4.030626
C	-1.614079	-1.817375	3.652257
C	-3.132701	-2.960704	5.760550
C	-1.831331	-3.210515	3.720917
C	-2.150421	-0.996398	4.678207
C	-2.914031	-1.575433	5.716971
C	-2.579419	-3.781827	4.762791
C	5.950124	-0.125511	2.071466
C	6.786849	-2.667258	2.915307
C	5.139422	-1.249626	1.787331
C	7.156432	-0.267830	2.776365
C	7.577054	-1.539992	3.204029
C	5.578357	-2.523574	2.212693
C	-1.917193	0.464539	4.697947
C	-2.857847	1.486753	4.621254
H	-3.935041	1.464406	4.440500
N	-0.672935	1.028052	4.879140
N	-0.811756	2.318994	4.924414
N	-2.138170	2.643072	4.755873
C	-2.537841	3.995484	4.789130
C	-3.157596	6.664857	4.860535
C	-3.892451	4.359147	4.881228
C	-1.836430	6.208764	4.778557
C	-4.202935	5.724233	4.911735
N	-1.525016	4.891666	4.737613
Ir	0.534567	3.988949	5.134345
C	2.172775	2.877748	2.610564
C	-0.037626	2.719653	0.986197
C	2.150074	2.115374	1.440128
C	-0.086284	3.538712	2.120810
C	1.000535	3.544382	3.029867
N	1.047913	1.982577	0.671598
Zn	0.825117	0.095944	-0.281411
B	2.154631	2.986105	5.889318

B	-0.088076	4.025653	7.159860
B	1.506714	5.675518	5.873134
O	0.888143	6.935481	5.918096
O	2.815009	5.755968	6.335717
C	3.022912	7.065599	6.931868
C	2.802540	6.919447	8.445182
C	4.457789	7.508600	6.640303
C	1.904043	7.933410	6.223230
C	1.278968	9.015356	7.104019
C	2.361513	8.524349	4.880023
O	-1.318144	3.431574	7.489196
O	0.464254	4.649324	8.272816
C	-1.704311	3.910674	8.806654
C	-0.304564	4.255186	9.445208
C	-0.320384	5.416666	10.438942
C	0.397211	3.028067	10.046690
C	-2.491148	2.814445	9.522550
C	-2.578163	5.156210	8.576500
H	-3.803276	2.868178	-0.826906
H	-5.974296	3.696080	-1.772678
H	-6.936547	2.624741	-3.832328
H	-5.713997	0.724734	-4.931976
H	-3.546623	-0.094968	-3.974142
H	-4.678654	3.594710	4.945728
H	-5.251912	6.048447	4.987912
H	-3.359133	7.745462	4.890789
H	-0.968757	6.887226	4.787426
H	-0.107109	2.671856	10.967784
H	1.437735	3.308267	10.304420
H	0.450986	2.207907	9.303704
H	0.699753	5.583505	10.839500
H	-0.653226	6.356911	9.959615
H	-0.993227	5.193678	11.293127
H	-2.991858	5.560513	9.522617
H	-3.422392	4.881423	7.911742
H	-1.994495	5.951459	8.069337
H	-1.934364	1.858532	9.522255
H	-3.458614	2.646300	9.006483
H	-2.706731	3.103852	10.572159
H	-0.911819	2.591443	0.325402
H	3.024682	1.523679	1.126238
H	3.064773	2.881955	3.252536
H	1.428244	4.808200	4.040158
H	3.491908	6.141830	8.830197
H	3.004655	7.867526	8.983766
H	1.773073	6.574763	8.654918
H	4.628259	8.548220	6.989691
H	5.166052	6.844591	7.175633
H	4.692347	7.449663	5.560875
H	2.048199	9.739697	7.443096
H	0.789922	8.574126	7.992753

H	0.510907	9.573952	6.530651
H	3.097939	9.342131	5.014644
H	1.479702	8.934077	4.346804
H	2.813382	7.740517	4.239447
H	-1.890409	2.065211	-4.389329
H	0.687420	1.977956	-5.232823
H	4.308633	2.709598	-3.588794
H	5.501534	3.379053	-5.690474
H	5.026079	2.174052	-7.843931
H	3.353272	0.298961	-7.876569
H	2.169255	-0.362422	-5.767273
H	5.307592	0.147987	-3.173919
H	4.957607	-3.404414	1.985604
H	7.115282	-3.668345	3.237812
H	8.521823	-1.653467	3.759081
H	7.767314	0.621523	2.999059
H	5.612547	0.870351	1.743332
H	3.506334	-2.065704	3.809212
H	0.893815	-2.207470	4.545920
H	-3.315694	-0.923340	6.508937
H	-3.724236	-3.399286	6.579442
H	-2.737494	-4.871612	4.790518
H	-1.411123	-3.843992	2.924342
H	-3.763881	-0.934605	2.286218
H	-4.519004	0.239232	-0.052368
O	2.102671	2.239424	7.072031
O	3.424766	2.894670	5.309493
C	3.465709	1.854807	7.409541
C	4.148764	1.830677	5.989944
C	3.434684	0.512239	8.136602
C	4.030394	2.965961	8.310480
C	5.640428	2.161546	5.979480
C	3.873400	0.530999	5.218594
H	4.033529	3.931318	7.765052
H	3.368612	3.079898	9.191298
H	5.054878	2.732703	8.665543
H	4.464146	0.135477	8.310976
H	2.938571	0.630322	9.121655
H	2.870163	-0.243905	7.558842
H	5.829667	3.184430	6.355979
H	6.205967	1.436661	6.601374
H	6.030590	2.096912	4.943201
H	4.183947	0.654110	4.161944
H	2.792601	0.287157	5.220241
H	4.439322	-0.327060	5.635086
H	-1.009597	4.099194	2.335121
C	5.066165	-0.491999	-1.059785
H	6.070909	-0.786970	-0.735308

B

Zero-point correction= 1.302166 (Hartree/Particle)
Thermal correction to Energy= 1.419709
Thermal correction to Enthalpy= 1.420827
Thermal correction to Gibbs Free Energy= 1.142783

solvent: -5768.46983856

N	3.517041	0.303874	-0.633628
C	3.952870	1.265801	-1.516447
C	4.501989	-0.654029	-0.548809
C	5.241917	0.868732	-2.068711
C	4.489626	-1.773323	0.330626
C	3.387513	-2.161956	1.144133
N	2.121177	-1.626932	1.084713
C	1.355500	-2.284982	2.021017
C	2.183405	-3.247057	2.739570
C	3.440791	-3.180680	2.185520
C	-0.036660	-2.073799	2.223026
C	-0.804079	-1.070325	1.569073
N	-0.285505	0.021365	0.912305
C	-1.330341	0.890451	0.689630
C	-2.251285	-0.950775	1.652443
C	-2.578249	0.278019	1.125694
C	-1.208849	2.217700	0.189025
C	-0.040630	2.730471	-0.442933
N	1.102682	2.003083	-0.693635
C	1.971580	2.830926	-1.367263
C	0.124831	4.088344	-0.945274
C	1.366791	4.146662	-1.533065
C	3.274196	2.478480	-1.820516
C	4.148248	4.855922	-4.683508
C	5.231407	4.028539	-2.228852
C	3.468918	3.909333	-3.899196
C	5.371482	5.392823	-4.243135
C	5.910311	4.975736	-3.012593
C	4.000041	3.480093	-2.659849
C	-3.876618	4.165864	2.084541
C	-2.978812	3.901454	-0.566667
C	-4.472577	4.931407	1.066400
C	-2.836738	3.278072	1.775140
C	-2.356055	3.136901	0.448287
C	-4.022934	4.790515	-0.259220
C	-0.731896	-2.916396	3.243397
C	-1.997616	-4.606853	5.162275
C	-0.707055	-4.326567	3.122627
C	-1.434306	-2.354608	4.358941
C	-2.049346	-3.212825	5.300494
C	-1.326421	-5.165328	4.060918
C	6.963034	-1.994909	0.816821

C	6.896916	-4.740670	0.237063
C	5.737866	-2.588003	0.428083
C	8.135632	-2.761402	0.913957
C	8.107247	-4.137552	0.624161
C	5.724352	-3.973887	0.140347
C	-1.595668	-0.899191	4.548600
C	-2.730375	-0.218930	4.996671
H	-3.711602	-0.574345	5.319583
N	-0.627840	0.031806	4.250152
N	-1.111752	1.216163	4.465704
N	-2.401637	1.107555	4.929001
C	-3.087009	2.278731	5.310544
C	-4.284321	4.645964	6.010017
C	-4.475760	2.285159	5.517736
C	-2.908492	4.555477	5.770692
C	-5.081606	3.493971	5.884207
N	-2.308959	3.387334	5.433731
Ir	-0.227553	3.157529	4.867127
C	3.942035	2.086719	3.838211
C	1.421527	1.948794	2.824428
C	3.673504	1.421633	2.633235
C	1.567021	2.601620	4.065751
C	2.886392	2.678780	4.561020
N	2.418852	1.364415	2.139110
Zn	1.744590	0.338176	0.436958
B	0.306868	2.129464	6.624281
B	0.503464	4.579700	6.167214
B	-0.025212	4.985735	3.699280
O	-0.822720	6.121690	3.936288
O	1.026845	5.267795	2.836067
C	1.038022	6.700577	2.595391
C	2.037007	7.318264	3.582697
C	1.477001	6.949179	1.152718
C	-0.462609	7.092958	2.905249
C	-0.657376	8.503610	3.456546
C	-1.400765	6.839541	1.713690
O	-0.287522	4.990984	7.255264
O	1.748166	5.204896	6.202332
C	0.384210	6.101179	7.909459
C	1.890704	5.864913	7.493340
C	2.713964	7.141413	7.319147
C	2.623792	4.881169	8.419126
C	0.115661	6.021436	9.412143
C	-0.212196	7.385720	7.313062
H	-2.355185	2.691428	2.568745
H	-4.206614	4.272369	3.129876
H	-5.284957	5.636573	1.303780
H	-4.495228	5.373957	-1.065753
H	-2.645291	3.780246	-1.608765
H	-5.065793	1.369837	5.373714
H	-6.168159	3.536377	6.053107

H	-4.721495	5.616872	6.283869
H	-2.234650	5.419650	5.837485
H	2.821144	5.321398	9.417464
H	3.594430	4.614602	7.955139
H	2.048786	3.941743	8.529515
H	3.737301	6.881853	6.981389
H	2.274101	7.821920	6.566960
H	2.793857	7.685043	8.283401
H	0.217001	8.301661	7.766739
H	-1.306369	7.388950	7.494391
H	-0.057496	7.407227	6.216054
H	0.381708	5.025752	9.814707
H	-0.961463	6.192429	9.612785
H	0.696267	6.793959	9.957749
H	0.433755	1.869193	2.348706
H	4.452028	0.921009	2.033831
H	4.976275	2.133612	4.215541
H	3.077790	3.192935	5.513189
H	3.022237	6.832273	3.440260
H	2.152686	8.410389	3.428834
H	1.725597	7.114453	4.623733
H	1.381704	8.024276	0.893659
H	2.537846	6.653248	1.028669
H	0.883972	6.346830	0.441552
H	-0.320049	9.258129	2.716217
H	-0.093962	8.658188	4.395798
H	-1.731462	8.682989	3.666822
H	-1.238714	7.570362	0.896146
H	-2.451882	6.918195	2.054339
H	-1.264369	5.815602	1.310919
H	-0.605727	4.899207	-0.854350
H	1.842628	5.013182	-2.005921
H	5.645071	3.708401	-1.259784
H	6.864306	5.396501	-2.656254
H	5.904216	6.135527	-4.858240
H	3.721998	5.172096	-5.649153
H	2.514708	3.481928	-4.245029
H	5.803635	1.419499	-2.831837
H	4.777859	-4.442188	-0.171992
H	6.866763	-5.816586	0.001233
H	9.027120	-4.739072	0.699798
H	9.077499	-2.281655	1.225094
H	6.981205	-0.919525	1.053597
H	4.331455	-3.749227	2.476595
H	1.857430	-3.880773	3.572051
H	-2.554039	-2.768760	6.173614
H	-2.477194	-5.253725	5.913360
H	-1.288003	-6.258118	3.928395
H	-0.194640	-4.760898	2.250766
H	-2.928188	-1.710620	2.061091
H	-3.575100	0.718809	1.008666

O	-0.645525	1.303846	7.232761
O	1.557670	1.959867	7.201804
C	-0.004660	0.611481	8.343804
C	1.519889	0.704406	7.943350
C	-0.562553	-0.810522	8.410848
C	-0.340461	1.403194	9.615825
C	2.493279	0.792496	9.116930
C	1.947348	-0.391790	6.952463
H	0.067428	2.432324	9.550228
H	-1.442116	1.482921	9.709780
H	0.056512	0.913325	10.527929
H	-0.013309	-1.416450	9.161014
H	-1.631442	-0.782791	8.706610
H	-0.493249	-1.313232	7.427003
H	2.297062	1.681291	9.746133
H	2.423789	-0.115383	9.751330
H	3.531352	0.868581	8.735666
H	2.938662	-0.125616	6.535277
H	1.242037	-0.455958	6.098414
H	2.020573	-1.385713	7.438356
H	-0.677465	3.833880	3.299446
C	5.582864	-0.321258	-1.468281
H	6.476034	-0.930641	-1.647291

B^{para}

Zero-point correction= 1.301040 (Hartree/Particle)
Thermal correction to Energy= 1.419272
Thermal correction to Enthalpy= 1.420391
Thermal correction to Gibbs Free Energy= 1.138376

solvent: -5768.48467781

N	2.748751	-0.260858	-0.881334
C	3.175769	-0.073019	-2.178133
C	3.845187	-0.651191	-0.144696
C	4.594313	-0.390612	-2.276104
C	3.850596	-0.952864	1.246307
C	2.694291	-1.032281	2.068849
N	1.397347	-0.832336	1.647815
C	0.587948	-1.027756	2.745101
C	1.396408	-1.349821	3.911837
C	2.705509	-1.343347	3.495572
C	-0.823872	-0.926861	2.763710
C	-1.637916	-0.514479	1.677850
N	-1.186956	-0.140430	0.431737
C	-2.295207	0.197797	-0.313970
C	-3.091259	-0.432144	1.721343
C	-3.500225	0.015808	0.485966
C	-2.281783	0.636455	-1.665522
C	-1.127695	0.712713	-2.492540

N	0.150226	0.351322	-2.125324
C	0.963456	0.577318	-3.213730
C	-1.123276	1.204528	-3.864317
C	0.173016	1.115841	-4.313342
C	2.366394	0.350892	-3.266719
C	3.368399	0.030319	-6.942273
C	4.036540	1.592142	-4.706730
C	2.725973	-0.191027	-5.712908
C	4.347190	1.033686	-7.058348
C	4.678903	1.813832	-5.936031
C	3.050329	0.586469	-4.576652
C	-5.524188	2.553890	-2.309350
C	-4.156155	0.330282	-3.343315
C	-6.074886	1.832810	-3.384081
C	-4.293924	2.164028	-1.754802
C	-3.590676	1.046965	-2.263155
C	-5.386222	0.719679	-3.898878
C	-1.542022	-1.379236	4.002717
C	-3.077947	-2.320397	6.199932
C	-1.715999	-2.761173	4.231333
C	-2.128601	-0.466022	4.918033
C	-2.899872	-0.942853	6.000959
C	-2.471601	-3.231681	5.317740
C	6.155780	-0.159591	1.886600
C	6.735192	-2.593236	3.156156
C	5.179870	-1.183985	1.889614
C	7.397671	-0.344262	2.514568
C	7.690536	-1.560643	3.156740
C	5.493683	-2.407870	2.525962
C	-1.891779	0.986360	4.788684
C	-2.796470	2.027274	4.599722
H	-3.872428	2.025883	4.410952
N	-0.631343	1.520982	4.925539
N	-0.725401	2.811485	4.841322
N	-2.037304	3.167233	4.626180
C	-2.377798	4.532470	4.498757
C	-2.865393	7.218343	4.257339
C	-3.715219	4.957474	4.441898
C	-1.566836	6.698085	4.327296
C	-3.955924	6.331757	4.314283
N	-1.323077	5.375370	4.438874
Ir	0.679924	4.388869	4.893245
C	2.176949	3.182782	2.389526
C	-0.073013	2.748274	0.890420
C	2.170595	2.348954	1.266069
C	-0.143679	3.589919	2.004734
C	0.969774	3.747124	2.870285
N	1.050564	2.077824	0.560072
Zn	0.824136	0.099610	-0.167765
B	2.316014	3.291655	5.474231
B	0.071714	4.342960	6.981240

B	1.345532	5.895966	6.208823
O	0.647484	7.106464	6.383406
O	2.574064	5.934227	6.869604
C	2.613062	7.122762	7.697518
C	2.164398	6.715533	9.109861
C	4.048696	7.649862	7.726090
C	1.573249	8.067438	6.967817
C	0.792515	8.999497	7.893645
C	2.196986	8.852896	5.803056
O	-1.313097	4.347799	7.182389
O	0.736459	3.940182	8.125965
C	-1.583278	3.989164	8.566545
C	-0.233719	3.279387	8.987187
C	0.180653	3.488528	10.442873
C	-0.202550	1.786566	8.620171
C	-2.818053	3.086535	8.591302
C	-1.844908	5.298698	9.323573
H	-3.855197	2.730537	-0.918106
H	-6.053446	3.431564	-1.904482
H	-7.039184	2.138295	-3.820528
H	-5.813049	0.145653	-4.736937
H	-3.618589	-0.545390	-3.739504
H	-4.542727	4.238068	4.507280
H	-4.989810	6.706261	4.267061
H	-3.015254	8.303514	4.162797
H	-0.673644	7.338546	4.324701
H	-0.872655	1.189153	9.270932
H	0.833091	1.412862	8.731463
H	-0.477160	1.626299	7.559041
H	1.140472	2.967783	10.633238
H	0.325776	4.559510	10.678417
H	-0.582236	3.070646	11.131886
H	-2.118728	5.119594	10.382815
H	-2.675748	5.840799	8.829229
H	-0.950326	5.952158	9.289341
H	-2.699894	2.234689	7.893772
H	-3.712455	3.663914	8.279828
H	-3.000581	2.690389	9.611514
H	-0.959919	2.533904	0.270675
H	3.075562	1.802794	0.950032
H	3.110674	3.317255	2.952080
H	1.757266	5.543011	4.564844
H	2.799060	5.876757	9.456749
H	2.249749	7.554671	9.829348
H	1.121796	6.345728	9.097391
H	4.101754	8.621703	8.259450
H	4.698662	6.927059	8.259060
H	4.454333	7.776590	6.704928
H	1.481618	9.673004	8.443854
H	0.193102	8.427640	8.626858
H	0.096590	9.627812	7.301232

H	2.872540	9.656314	6.159711
H	1.386176	9.320434	5.208167
H	2.769023	8.178999	5.133909
H	-1.995603	1.584585	-4.407956
H	0.564139	1.411247	-5.293442
H	4.288745	2.206331	-3.828097
H	5.440249	2.605967	-6.019312
H	4.851117	1.207353	-8.022505
H	3.106744	-0.589756	-7.814692
H	1.964496	-0.980883	-5.617212
H	5.188162	-0.371513	-3.196934
H	4.749976	-3.219814	2.515063
H	6.961201	-3.554737	3.644487
H	8.663337	-1.705753	3.652945
H	8.137307	0.472197	2.511726
H	5.918402	0.797429	1.396762
H	3.600545	-1.521110	4.100862
H	1.009660	-1.520281	4.923086
H	-3.337015	-0.216292	6.704776
H	-3.676775	-2.681614	7.050620
H	-2.595548	-4.315592	5.469872
H	-1.258844	-3.469060	3.522629
H	-3.717801	-0.707519	2.578421
H	-4.525768	0.178871	0.135114
O	2.228862	2.161500	6.280806
O	3.640073	3.573503	5.135600
C	3.568758	1.873030	6.765044
C	4.467840	2.473517	5.613862
C	3.700348	0.364014	6.963937
C	3.738347	2.627679	8.094976
C	5.810393	3.041346	6.074506
C	4.664642	1.499576	4.443193
H	3.610141	3.717590	7.937659
H	2.943053	2.304618	8.794505
H	4.724613	2.425177	8.560216
H	4.747189	0.084863	7.204673
H	3.053259	0.038011	7.803797
H	3.379159	-0.182308	6.057757
H	5.669223	3.876077	6.786492
H	6.425925	2.253383	6.556642
H	6.371655	3.430535	5.200754
H	5.121200	2.042509	3.591809
H	3.696805	1.082493	4.100938
H	5.339534	0.659107	4.701977
H	-1.115632	4.058774	2.226216
C	5.010931	-0.742758	-1.013882
H	6.010479	-1.067944	-0.703798

TS$_{BC}$*^{para}*

Zero-point correction= 1.299812 (Hartree/Particle)

Thermal correction to Energy= 1.417296

Thermal correction to Enthalpy= 1.418414
Thermal correction to Gibbs Free Energy= 1.138198

solvent: -5768.43007890

N	2.648724000	-1.191510000	-1.075622000
C	3.105733000	-0.721018000	-2.286740000
C	3.449306000	-2.254553000	-0.724301000
C	4.207890000	-1.554565000	-2.750357000
C	3.365663000	-2.987217000	0.491017000
C	2.357365000	-2.818605000	1.479846000
N	1.227282000	-2.037144000	1.355415000
C	0.516997000	-2.165333000	2.532578000
C	1.248361000	-3.022577000	3.454982000
C	2.385033000	-3.435770000	2.798524000
C	-0.744107000	-1.564779000	2.800553000
C	-1.402503000	-0.664397000	1.919083000
N	-0.819530000	-0.018262000	0.852257000
C	-1.754962000	0.859886000	0.353261000
C	-2.792767000	-0.252703000	2.039070000
C	-3.009682000	0.709018000	1.079071000
C	-1.545038000	1.778672000	-0.709948000
C	-0.420239000	1.774072000	-1.578676000
N	0.583362000	0.828307000	-1.579197000
C	1.443646000	1.150846000	-2.606659000
C	-0.162959000	2.761206000	-2.617211000
C	0.992240000	2.372865000	-3.257410000
C	2.596300000	0.407280000	-2.984488000
C	3.382524000	1.276046000	-6.623131000
C	4.681678000	1.261090000	-4.134003000
C	2.691527000	0.862374000	-5.472504000
C	4.725429000	1.684579000	-6.533315000
C	5.372432000	1.675598000	-5.284473000
C	3.330327000	0.848397000	-4.210003000
C	-3.912934000	4.712379000	-0.104656000
C	-3.295559000	2.899373000	-2.155908000
C	-4.599488000	4.783645000	-1.329923000
C	-2.921634000	3.737863000	0.097086000
C	-2.596364000	2.816943000	-0.928252000
C	-4.287909000	3.872799000	-2.355353000
C	-1.440231000	-1.949265000	4.069811000
C	-2.776383000	-2.800203000	6.441957000
C	-1.724026000	-3.315460000	4.312841000
C	-1.852125000	-0.998758000	5.054512000
C	-2.517728000	-1.438952000	6.219917000
C	-2.377424000	-3.741737000	5.478762000
C	5.791071000	-3.619760000	0.842847000
C	5.099484000	-6.316229000	1.210158000
C	4.430450000	-4.000500000	0.762683000
C	6.789984000	-4.572118000	1.102731000

C	6.448023000	-5.923992000	1.287028000
C	4.100517000	-5.363679000	0.951117000
C	-1.679552000	0.463993000	4.868270000
C	-2.698243000	1.367850000	4.574908000
H	-3.766293000	1.223879000	4.404533000
N	-0.487144000	1.136093000	4.901099000
N	-0.719466000	2.396321000	4.611628000
N	-2.071904000	2.563654000	4.383525000
C	-2.554125000	3.816239000	3.996906000
C	-3.298419000	6.309722000	3.144504000
C	-3.926605000	4.116703000	3.948449000
C	-1.962066000	5.937796000	3.251623000
C	-4.307335000	5.388012000	3.511607000
N	-1.567864000	4.705583000	3.684818000
Ir	0.394802000	4.201684000	4.521506000
Zn	1.097715000	-0.387982000	0.043834000
B	2.493966345	3.898275550	4.855715878
B	-0.021375000	4.154811000	6.586324000
B	0.478702000	6.035074000	5.591112000
O	-0.620190000	6.899636000	5.648351000
O	1.602690000	6.619977000	6.168975000
C	1.188067000	7.830504000	6.850565000
C	0.934853000	7.462594000	8.320502000
C	2.316817000	8.856487000	6.741617000
C	-0.135453000	8.207951000	6.069522000
C	-1.214518000	8.879815000	6.916959000
C	0.134016000	9.013708000	4.788875000
O	-1.297803000	3.696792000	6.930042000
O	0.815745000	4.181758000	7.688328000
C	-1.316511000	3.410199000	8.357108000
C	0.227641000	3.299566000	8.686303000
C	0.629813000	3.792358000	10.074810000
C	0.799628000	1.899262000	8.408044000
C	-2.106439000	2.117896000	8.573146000
C	-2.005960000	4.595631000	9.045903000
H	-2.376922000	3.691012000	1.053809000
H	-4.141891000	5.422729000	0.703709000
H	-5.376796000	5.548730000	-1.485908000
H	-4.826014000	3.915766000	-3.315882000
H	-3.056888000	2.177535000	-2.952566000
H	-4.672254000	3.369812000	4.254302000
H	-5.371476000	5.659789000	3.453623000
H	-3.551583000	7.313494000	2.773379000
H	-1.147162000	6.630979000	3.003756000
H	0.480734000	1.161907000	9.172369000
H	1.905050000	1.964192000	8.405563000
H	0.497028000	1.538288000	7.403703000
H	1.724339000	3.678224000	10.208081000
H	0.381408000	4.861240000	10.215379000
H	0.121782000	3.199841000	10.863560000
H	-2.120257000	4.433051000	10.136637000

H	-3.011329000	4.735711000	8.600947000
H	-1.432520000	5.529549000	8.881881000
H	-1.721714000	1.308188000	7.924109000
H	-3.173886000	2.281766000	8.320786000
H	-2.046757000	1.788989000	9.631252000
H	1.091597000	5.579627000	4.062257000
H	1.846385000	6.986774000	8.731889000
H	0.684471000	8.352083000	8.932712000
H	0.118554000	6.719840000	8.401045000
H	2.000148000	9.837616000	7.152181000
H	3.193958000	8.506159000	7.322238000
H	2.640951000	8.992089000	5.692807000
H	-0.840063000	9.832417000	7.345463000
H	-1.543448000	8.221928000	7.743190000
H	-2.100788000	9.106368000	6.290331000
H	0.450853000	10.052641000	5.010476000
H	-0.797916000	9.055464000	4.189295000
H	0.919175000	8.527281000	4.175011000
H	-0.778029000	3.644637000	-2.822964000
H	1.500719000	2.877634000	-4.086679000
H	5.183576000	1.260271000	-3.153746000
H	6.422476000	1.999497000	-5.203931000
H	5.267440000	2.009079000	-7.435849000
H	2.870173000	1.272827000	-7.598572000
H	1.642968000	0.532968000	-5.541820000
H	4.734002000	-1.439302000	-3.704761000
H	3.044814000	-5.669595000	0.882248000
H	4.823241000	-7.374076000	1.346771000
H	7.231699000	-6.671030000	1.490558000
H	7.843061000	-4.254796000	1.168436000
H	6.055470000	-2.559256000	0.707961000
H	3.182710000	-4.081831000	3.182392000
H	0.944990000	-3.265233000	4.479596000
H	-2.823473000	-0.687620000	6.965293000
H	-3.290209000	-3.121906000	7.361330000
H	-2.584550000	-4.813189000	5.628804000
H	-1.430830000	-4.050269000	3.547443000
H	-3.513464000	-0.680131000	2.746225000
H	-3.940158000	1.242766000	0.853105000
O	3.063441345	3.090083550	5.857634878
O	3.394653345	4.912564550	4.466088878
C	4.279619345	3.730354550	6.315057878
C	4.678052345	4.626460550	5.068519878
C	5.296135345	2.641672550	6.668399878
C	3.934953345	4.565875550	7.558673878
C	5.350275345	5.953485550	5.425967878
C	5.501564345	3.858423550	4.020243878
H	3.187703345	5.341765550	7.307355878
H	3.475180345	3.902605550	8.316776878
H	4.840497345	5.033494550	7.997108878
H	6.280303345	3.087644550	6.922539878

H	4.939080345	2.072081550	7.550631878
H	5.432807345	1.925450550	5.836171878
H	4.664570345	6.590078550	6.015881878
H	6.281871345	5.784092550	6.004890878
H	5.613726345	6.502533550	4.498944878
H	5.559716345	4.467864550	3.095943878
H	5.009544345	2.898781550	3.762092878
H	6.533130345	3.649640550	4.369676878
C	4.420278000	-2.506527000	-1.781809000
H	5.153198000	-3.321200000	-1.791116000
N	2.020933000	1.085878000	1.280412000
C	2.208660000	0.704643000	2.562739000
C	1.891667000	2.403049000	1.011819000
C	2.113694000	1.595241000	3.629255000
H	2.374495000	-0.369678000	2.732271000
C	1.899078000	3.378838000	2.021140000
H	1.735494000	2.665375000	-0.048066000
C	1.914080655	2.975552450	3.381677122
H	2.177240000	1.222569000	4.659044000
H	1.841294000	4.443485000	1.749704000

TS_{BC}

Zero-point correction= 1.301358 (Hartree/Particle)
Thermal correction to Energy= 1.418406
Thermal correction to Enthalpy= 1.419524
Thermal correction to Gibbs Free Energy= 1.140635

solvent: -5768.46818674

N	2.321768	-0.375884	-0.529687
C	2.773667	-0.060539	-1.791969
C	3.333840	-1.064278	0.101149
C	4.109529	-0.608605	-1.988710
C	3.303990	-1.555327	1.437461
C	2.155366	-1.549660	2.279044
N	0.890460	-1.165060	1.892760
C	0.060195	-1.365162	2.975175
C	0.831833	-1.858311	4.106938
C	2.131588	-1.973980	3.675462
C	-1.338101	-1.147377	2.984507
C	-2.094047	-0.591748	1.922270
N	-1.575615	-0.008325	0.787170
C	-2.626698	0.585440	0.121334
C	-3.535898	-0.413461	1.954617
C	-3.866205	0.344106	0.851116
C	-2.515536	1.336919	-1.081291
C	-1.334235	1.449496	-1.862899
N	-0.113780	0.879635	-1.567838
C	0.714377	1.121767	-2.644913
C	-1.270317	2.100252	-3.165077

C	0.002457	1.903341	-3.646360
C	2.058813	0.679172	-2.771113
C	3.043892	0.818016	-6.464144
C	3.915848	1.864061	-4.009133
C	2.358484	0.497028	-5.280753
C	4.166854	1.664013	-6.424258
C	4.601323	2.185287	-5.192358
C	2.783342	1.017263	-4.036258
C	-4.866402	4.166246	-2.103328
C	-4.896699	1.359589	-1.976681
C	-6.023646	3.462825	-2.482664
C	-3.733821	3.466800	-1.655636
C	-3.732556	2.054340	-1.574208
C	-6.031734	2.057558	-2.422487
C	-2.048915	-1.377015	4.285021
C	-3.217108	-1.769469	6.833357
C	-2.429618	-2.665152	4.710076
C	-2.272324	-0.270637	5.151838
C	-2.840866	-0.479443	6.425524
C	-3.017313	-2.862242	5.971667
C	5.762098	-1.371134	1.994425
C	5.802643	-3.983586	3.015082
C	4.569361	-2.133772	1.980514
C	6.952116	-1.901286	2.515440
C	6.976680	-3.209335	3.031710
C	4.612110	-3.452480	2.492919
C	-1.820034	1.081807	4.748881
C	-2.237964	1.893284	3.697475
H	-3.017311	1.761755	2.942271
N	-0.789430	1.710803	5.405413
N	-0.547462	2.846852	4.815571
N	-1.424109	2.991417	3.755507
C	-1.323199	4.121343	2.921381
C	-1.019196	6.416951	1.451002
C	-2.102382	4.264490	1.757167
C	-0.265398	6.163134	2.601681
C	-1.944424	5.445682	1.022472
N	-0.411906	5.032768	3.321791
Ir	1.022992	4.275057	4.945151
Zn	0.465809	0.135749	0.314362
B	2.846455	3.155682	4.800764
B	1.144677	3.685773	6.930988
B	0.545926	5.961577	6.067574
O	-0.406351	6.865886	5.568058
O	1.195746	6.498807	7.179467
C	0.537948	7.745041	7.543809
C	-0.471509	7.435289	8.658309
C	1.598991	8.726456	8.044654
C	-0.161281	8.157787	6.188743
C	-1.492812	8.892974	6.341869
C	0.774679	8.933933	5.245735

O	2.195245	3.151335	7.665873
O	-0.058066	3.595042	7.645757
C	1.606471	2.303958	8.696405
C	0.222087	3.018587	8.948968
C	-0.928822	2.079521	9.315969
C	0.324577	4.173909	9.953127
C	2.547810	2.267266	9.899467
C	1.423181	0.899294	8.095460
H	-2.817119	4.007543	-1.373609
H	-4.845907	5.266743	-2.157429
H	-6.914311	4.007920	-2.833008
H	-6.927643	1.497800	-2.735251
H	-4.898145	0.258835	-1.947796
H	-2.790523	3.473340	1.424383
H	-2.544632	5.604233	0.114374
H	-0.877222	7.355271	0.895678
H	0.481475	6.869895	2.990249
H	1.140170	4.864323	9.660277
H	0.503373	3.809304	10.985123
H	-0.625981	4.742295	9.943841
H	-1.854343	2.668594	9.479935
H	-0.704418	1.520807	10.248535
H	-1.122148	1.356796	8.501144
H	1.039424	0.183646	8.850853
H	2.396107	0.530681	7.722529
H	0.732219	0.930370	7.229963
H	3.480995	1.733343	9.630160
H	2.823475	3.286888	10.229060
H	2.076470	1.730103	10.748754
H	-1.216666	6.689848	8.316256
H	-0.998688	8.348963	8.999840
H	0.067983	7.003921	9.522419
H	1.152687	9.724704	8.235815
H	2.032691	8.354326	8.994588
H	2.426491	8.836417	7.318861
H	-1.358094	9.840239	6.904321
H	-2.239593	8.268721	6.867163
H	-1.902364	9.141279	5.341465
H	0.983241	9.959064	5.612971
H	1.737105	8.397231	5.120594
H	0.291444	9.017560	4.250773
H	-2.099727	2.616493	-3.661592
H	0.418354	2.246950	-4.600077
H	4.252058	2.271605	-3.042607
H	5.478992	2.850138	-5.152325
H	4.704460	1.914928	-7.352449
H	2.702380	0.398415	-7.423929
H	1.484635	-0.172683	-5.307963
H	4.692025	-0.547333	-2.914916
H	3.697909	-4.065512	2.465302
H	5.815193	-5.013983	3.404813

H	7.909606	-3.625863	3.443519
H	7.864582	-1.283983	2.527619
H	5.736800	-0.340411	1.609933
H	3.000345	-2.297881	4.257474
H	0.430832	-2.074159	5.103859
H	-2.982486	0.383408	7.094729
H	-3.664579	-1.921533	7.828070
H	-3.310507	-3.876124	6.287074
H	-2.244224	-3.518556	4.039499
H	-4.203067	-0.795056	2.737235
H	-4.861644	0.695849	0.557914
O	4.095172	3.787510	4.618938
O	3.022523	1.813712	5.166419
C	5.089533	2.740543	4.491825
C	4.447650	1.572722	5.344612
C	5.220581	2.374270	3.000974
C	6.423995	3.274237	5.015297
C	4.750150	0.165799	4.833752
C	4.807316	1.689157	6.832264
H	6.312631	3.708895	6.026059
H	6.797055	4.072033	4.340909
H	7.185768	2.467839	5.050801
H	5.414894	3.297028	2.417874
H	4.293428	1.913177	2.607704
H	6.060265	1.669818	2.834419
H	4.315237	0.002960	3.832033
H	4.301905	-0.584366	5.515514
H	5.841517	-0.024430	4.780350
H	5.885296	1.486150	6.994590
H	4.540657	2.685343	7.230207
H	4.231970	0.938085	7.405944
C	4.456295	-1.233689	-0.815771
H	5.376288	-1.788565	-0.601411
N	1.201034	1.929248	1.222745
C	1.685863	2.858235	0.368999
C	1.324650	2.129046	2.554503
C	2.348110	4.006872	0.820470
H	1.543730	2.648321	-0.702981
C	1.911657	3.287149	3.120296
H	0.947137	1.314002	3.195660
C	2.471760	4.210362	2.201172
H	2.759324	4.725489	0.094611
H	3.005229	5.089111	2.595224
H	2.183272	5.370610	4.947532

C

Zero-point correction= 1.303284 (Hartree/Particle)
Thermal correction to Energy= 1.420844
Thermal correction to Enthalpy= 1.421962
Thermal correction to Gibbs Free Energy= 1.142298

solvent: -5768.49385909

N	2.383009	0.359815	-0.862326
C	2.793438	1.197192	-1.874846
C	3.518824	-0.164929	-0.290259
C	4.245280	1.140443	-2.004577
C	3.566217	-0.996198	0.859257
C	2.446088	-1.512762	1.561999
N	1.124028	-1.248834	1.274601
C	0.388756	-1.735339	2.338063
C	1.265920	-2.397066	3.290626
C	2.542307	-2.269960	2.804626
C	-0.999093	-1.532928	2.533826
C	-1.865252	-0.946896	1.577167
N	-1.471270	-0.191962	0.491979
C	-2.624529	0.284866	-0.099935
C	-3.312440	-0.968682	1.671985
C	-3.788399	-0.210569	0.622374
C	-2.679461	1.202503	-1.182578
C	-1.549641	1.749534	-1.843914
N	-0.229600	1.464089	-1.559957
C	0.536538	2.146200	-2.484126
C	-1.622427	2.675250	-2.965400
C	-0.327045	2.940639	-3.346634
C	1.947208	2.050609	-2.629176
C	3.049237	3.535039	-5.979143
C	3.400110	4.024699	-3.237559
C	2.434398	2.698878	-5.031840
C	3.841218	4.618413	-5.558370
C	4.016370	4.860273	-4.184036
C	2.599836	2.934552	-3.648492
C	-5.786720	3.325412	-1.888544
C	-4.880779	0.702118	-2.317124
C	-6.614648	2.399425	-2.547280
C	-4.510175	2.939448	-1.445970
C	-4.037712	1.623770	-1.654697
C	-6.157638	1.086201	-2.759259
C	-1.537896	-1.798041	3.912720
C	-2.240819	-2.322445	6.611155
C	-1.783358	-3.113237	4.357706
C	-1.680658	-0.718199	4.844694
C	-2.013276	-1.004318	6.187185
C	-2.138313	-3.379846	5.691019
C	5.546253	-0.090602	2.098036
C	6.779473	-2.611690	2.187579
C	4.909542	-1.198394	1.490447
C	6.786406	-0.242116	2.738436
C	7.405743	-1.503843	2.786810
C	5.540068	-2.460449	1.541648
C	-1.390500	0.682301	4.461759

C	-1.754479	1.382633	3.314305
H	-2.444264	1.148620	2.503387
N	-0.554580	1.490031	5.210323
N	-0.329226	2.592858	4.556943
N	-1.067233	2.559215	3.375379
C	-0.837781	3.509140	2.356085
C	-0.185335	5.368088	0.446500
C	-1.569170	3.508639	1.155479
C	0.483948	5.291895	1.671950
C	-1.212209	4.444052	0.177153
N	0.187830	4.352402	2.599741
Ir	1.278496	4.065180	4.513234
Zn	0.509521	0.382622	0.078678
B	2.801702	1.092370	4.996051
B	1.974728	4.115039	6.443809
B	0.321286	5.710148	5.227467
O	-0.657613	5.712224	6.224902
O	0.482698	6.985645	4.676888
C	-0.221135	7.923960	5.537847
C	0.802419	8.429274	6.567681
C	-0.755591	9.077638	4.689582
C	-1.329702	7.002915	6.180115
C	-1.756304	7.395983	7.594084
C	-2.560108	6.830526	5.273533
O	1.580692	3.272948	7.499714
O	2.789835	5.149712	6.946856
C	1.898085	3.940341	8.753363
C	3.111108	4.851731	8.329139
C	3.232983	6.160259	9.110539
C	4.451254	4.096019	8.320012
C	2.215413	2.906019	9.832728
C	0.653730	4.747880	9.157793
H	-3.869510	3.665066	-0.921409
H	-6.138192	4.354501	-1.712225
H	-7.615620	2.700631	-2.894560
H	-6.798654	0.355430	-3.277721
H	-4.518051	-0.323989	-2.485845
H	-2.362238	2.772005	0.969977
H	-1.716736	4.433172	-0.800510
H	0.112561	6.121891	-0.296365
H	1.305947	5.968578	1.947178
H	4.359303	3.158742	7.735912
H	4.808541	3.858991	9.343379
H	5.212295	4.730423	7.822483
H	4.094903	6.746293	8.731970
H	3.397036	5.960043	10.190131
H	2.325849	6.784219	9.001631
H	0.783263	5.248745	10.139071
H	-0.210365	4.057075	9.222811
H	0.408670	5.497103	8.382034
H	1.319007	2.288498	10.042804

H	3.030768	2.226048	9.527661
H	2.517708	3.410992	10.773722
H	1.202492	7.583012	7.161486
H	0.370776	9.190207	7.249171
H	1.656872	8.882698	6.027046
H	-1.392317	9.751451	5.299974
H	0.089649	9.672876	4.288574
H	-1.348448	8.709227	3.830849
H	-2.205986	8.410778	7.597901
H	-0.902161	7.385299	8.296335
H	-2.513094	6.679048	7.971624
H	-3.181616	7.748176	5.235102
H	-2.250573	6.565540	4.241975
H	-3.180334	6.001097	5.668458
H	-2.547021	3.057201	-3.413539
H	0.012970	3.589628	-4.161541
H	3.535604	4.209186	-2.159874
H	4.635415	5.706691	-3.846098
H	4.324083	5.272683	-6.301463
H	2.913537	3.334969	-7.054021
H	1.819470	1.845246	-5.357606
H	4.837111	1.673437	-2.757468
H	5.047277	-3.325007	1.069649
H	7.261844	-3.601745	2.219137
H	8.375925	-1.625144	3.294158
H	7.263251	0.628559	3.215683
H	5.042687	0.889438	2.075956
H	3.472756	-2.626540	3.259303
H	0.942458	-2.869750	4.222042
H	-2.088051	-0.167553	6.898624
H	-2.502094	-2.522996	7.662048
H	-2.323644	-4.416870	6.012776
H	-1.666114	-3.938595	3.638165
H	-3.884290	-1.501111	2.441688
H	-4.832351	0.000281	0.363449
O	3.877882	1.272664	5.841972
O	2.066320	-0.042262	5.261288
C	4.019758	0.005625	6.571401
C	2.546163	-0.565117	6.538647
C	5.008151	-0.847883	5.760669
C	4.563929	0.279027	7.967845
C	2.460961	-2.090831	6.530605
C	1.632507	0.015753	7.624362
H	3.922138	0.994481	8.510418
H	5.584219	0.707263	7.904601
H	4.615639	-0.663249	8.551130
H	5.952749	-0.283689	5.633084
H	4.614685	-1.063766	4.746871
H	5.238835	-1.805739	6.268365
H	2.980472	-2.535712	5.662954
H	1.397140	-2.401822	6.498249

H	2.907865	-2.499428	7.460452
H	1.898329	-0.371408	8.629337
H	1.666311	1.122740	7.623469
H	0.590346	-0.279977	7.392089
C	4.696400	0.305312	-1.010526
H	5.730178	0.014955	-0.790080
N	1.451077	1.759774	1.500329
C	2.246105	2.780692	1.052451
C	1.615380	1.351437	2.764214
C	3.151836	3.435934	1.875752
H	2.122261	3.047454	-0.008176
C	2.520901	1.924837	3.701103
H	1.008486	0.489191	3.077478
C	3.262383	3.062327	3.250552
H	3.761545	4.258337	1.473409
H	4.075029	3.466962	3.870840
H	2.359356	5.213342	4.333725

TS_{CD}

Zero-point correction= 1.656211 (Hartree/Particle)
Thermal correction to Energy= 1.803147
Thermal correction to Enthalpy= 1.804265
Thermal correction to Gibbs Free Energy= 1.461004

solvent: -6591.43186452

N	2.222522	0.029477	-1.018142
C	2.718368	0.693928	-2.117484
C	3.301803	-0.491765	-0.342538
C	4.161590	0.495695	-2.201016
C	3.258606	-1.153938	0.914694
C	2.075892	-1.553887	1.590587
N	0.789101	-1.317486	1.154066
C	-0.053493	-1.724212	2.166276
C	0.721541	-2.272120	3.270608
C	2.043061	-2.166613	2.912977
C	-1.459754	-1.568956	2.185623
C	-2.239190	-0.963069	1.168513
N	-1.729355	-0.183807	0.146112
C	-2.799236	0.493160	-0.410737
C	-3.682240	-0.828161	1.219167
C	-4.033581	0.066606	0.231341
C	-2.705725	1.511181	-1.397707
C	-1.510756	1.872208	-2.079814
N	-0.275371	1.284198	-1.896474
C	0.586274	1.858261	-2.810483
C	-1.415641	2.898565	-3.108243
C	-0.119280	2.879114	-3.571478
C	1.968470	1.556334	-2.960660
C	3.102342	2.757042	-6.408199

C	3.696137	3.245038	-3.707865
C	2.418413	2.055888	-5.400958
C	4.085185	3.703979	-6.068524
C	4.379846	3.946293	-4.714681
C	2.707495	2.288752	-4.037024
C	-5.760635	3.774105	-1.001841
C	-4.525834	2.242226	-3.006630
C	-6.314104	3.738322	-2.294127
C	-4.593676	3.047669	-0.714777
C	-3.954157	2.273976	-1.713293
C	-5.693798	2.967905	-3.294213
C	-2.122785	-1.792064	3.513885
C	-3.175831	-2.199021	6.105891
C	-2.666818	-3.036858	3.880773
C	-2.121473	-0.721165	4.460513
C	-2.639939	-0.950509	5.756029
C	-3.199730	-3.243633	5.165437
C	5.228019	-0.111325	2.054217
C	6.254848	-2.608447	2.817464
C	4.549116	-1.289229	1.659979
C	6.398999	-0.180396	2.824857
C	6.914150	-1.430205	3.213452
C	5.082600	-2.539658	2.045258
C	-1.592014	0.620437	4.139078
C	-1.576101	1.323370	2.932073
H	-1.822598	1.065800	1.903356
N	-1.079400	1.449573	5.119185
N	-0.742300	2.590578	4.576814
N	-1.053852	2.541136	3.237828
C	-0.745071	3.628529	2.399874
C	0.050575	5.736140	0.849494
C	-1.273530	3.728652	1.105059
C	0.533255	5.544540	2.150200
C	-0.864299	4.810711	0.316695
N	0.136946	4.509139	2.924322
Ir	0.540698	4.237022	5.139469
Zn	0.297771	0.205398	-0.195217
B	3.147826	1.762128	4.367721
B	0.368188	4.134481	7.145645
B	-2.509210	5.726331	5.057749
O	-3.360027	5.781503	6.138161
O	-2.859606	4.738599	4.146168
C	-4.187125	4.254711	4.535704
C	-5.196180	5.072159	3.712293
C	-4.318219	2.770769	4.209118
C	-4.212396	4.594220	6.075934
C	-5.590734	4.947862	6.631303
C	-3.525332	3.525372	6.939247
O	0.990558	3.210596	8.004644
O	-0.463409	4.987547	7.884830
C	0.429463	3.354423	9.331623

C	-0.120313	4.834603	9.287393
C	-1.364432	5.088178	10.137474
C	0.972015	5.873713	9.591839
C	1.537637	3.103185	10.356236
C	-0.682975	2.301479	9.467992
H	-4.165440	3.079912	0.299425
H	-6.236806	4.374405	-0.210233
H	-7.229561	4.307353	-2.520928
H	-6.127183	2.925105	-4.306197
H	-4.046246	1.629505	-3.785886
H	-1.950037	2.960857	0.707909
H	-1.245676	4.904043	-0.711823
H	0.407219	6.591868	0.258369
H	1.281939	6.199761	2.617635
H	1.866271	5.673975	8.967734
H	1.257071	5.881529	10.663990
H	0.588411	6.876589	9.317027
H	-1.689414	6.141247	10.017701
H	-1.150603	4.906894	11.211748
H	-2.206019	4.438789	9.830542
H	-1.123775	2.289132	10.485932
H	-0.255472	1.301266	9.254249
H	-1.485813	2.487863	8.727928
H	1.858641	2.042387	10.306892
H	2.423024	3.733160	10.148826
H	1.181546	3.309774	11.387102
H	-5.126216	6.151524	3.954790
H	-6.237656	4.733577	3.884535
H	-4.958024	4.949054	2.636235
H	-5.319592	2.400352	4.510151
H	-4.204977	2.603307	3.118591
H	-3.556921	2.164467	4.727129
H	-6.292486	4.098100	6.500888
H	-6.014792	5.840492	6.133451
H	-5.511888	5.167616	7.714826
H	-4.141302	2.607791	7.028749
H	-2.530505	3.260830	6.530288
H	-3.360299	3.948767	7.948395
H	-2.234450	3.546493	-3.441066
H	0.323129	3.509130	-4.351482
H	3.919031	3.434521	-2.645757
H	5.144636	4.690381	-4.440198
H	4.621336	4.253217	-6.858669
H	2.869052	2.558737	-7.466516
H	1.650317	1.311517	-5.663339
H	4.809750	0.862448	-3.004868
H	4.566085	-3.461365	1.734601
H	6.657815	-3.590882	3.110876
H	7.827571	-1.486563	3.826460
H	6.896107	0.750583	3.138252
H	4.809429	0.865020	1.767468

H	2.919034	-2.452158	3.502051
H	0.303884	-2.667941	4.203363
H	-2.615637	-0.125321	6.484265
H	-3.579409	-2.355599	7.118698
H	-3.622309	-4.224701	5.434113
H	-2.651457	-3.854975	3.143770
H	-4.337848	-1.351216	1.925492
H	-5.039158	0.406804	-0.041027
O	4.321433	2.207803	4.926695
O	2.497555	0.794248	5.087002
C	4.450964	1.577883	6.248066
C	3.362006	0.404853	6.199417
C	5.894727	1.087574	6.385189
C	4.156041	2.654507	7.295301
C	3.948726	-0.963428	5.821488
C	2.495075	0.299685	7.454144
H	3.137764	3.073152	7.207703
H	4.868883	3.490294	7.162179
H	4.277539	2.254033	8.321759
H	6.581646	1.957698	6.364178
H	6.178551	0.403548	5.563998
H	6.036034	0.562120	7.351665
H	4.520834	-0.913829	4.873138
H	3.112592	-1.676394	5.681781
H	4.610961	-1.357739	6.617758
H	3.120048	0.063581	8.340249
H	1.939778	1.239516	7.635968
H	1.757595	-0.517594	7.321771
C	4.525996	-0.218729	-1.084289
H	5.528801	-0.553157	-0.793850
N	1.205240	1.756052	1.058499
C	1.772555	2.785253	0.389887
C	1.639336	1.482931	2.306353
C	2.792420	3.570304	0.942623
H	1.385063	2.971271	-0.623528
C	2.656221	2.211603	2.953131
H	1.152409	0.640058	2.820491
C	3.244559	3.273115	2.236833
H	4.042614	3.865652	2.705847
H	1.617825	3.229266	5.608630
B	-1.387485	6.998431	4.769594
O	-0.897217	7.855971	5.736950
O	-1.332686	7.550249	3.497665
C	-0.760487	8.893591	3.597449
C	0.688859	8.831925	3.104843
C	-1.586063	9.822388	2.703780
C	-0.878508	9.192348	5.150336
C	0.281133	9.994361	5.734859
C	-2.217858	9.840872	5.543546
H	1.291489	8.131743	3.715278
H	1.163038	9.833912	3.127005

H	0.699358	8.478975	2.054238
H	-1.252885	10.874492	2.819642
H	-1.452010	9.532776	1.641948
H	-2.665830	9.763330	2.936172
H	0.322004	11.005827	5.280762
H	1.248983	9.491523	5.563808
H	0.145505	10.107373	6.828797
H	-2.287510	10.891425	5.196410
H	-3.072904	9.268678	5.130984
H	-2.307889	9.824890	6.647673
B	2.058834	5.555040	5.470819
O	2.865890	5.626833	6.623538
O	2.573041	6.459521	4.501618
C	3.879947	6.920052	4.943915
C	4.924493	5.980171	4.316622
C	4.100549	8.355235	4.465423
C	3.774475	6.746776	6.508554
C	5.093480	6.404508	7.203758
C	3.103553	7.940928	7.205681
H	4.780694	4.937573	4.668847
H	5.961607	6.297744	4.548165
H	4.798796	5.998491	3.214685
H	5.064386	8.751127	4.847699
H	4.130302	8.385146	3.356971
H	3.289312	9.028882	4.798374
H	5.824639	7.231980	7.089805
H	5.542030	5.477974	6.800305
H	4.912763	6.248186	8.286406
H	3.725275	8.858561	7.167197
H	2.116771	8.143614	6.748082
H	2.925907	7.684614	8.268102
H	3.215492	4.402901	0.361236

D

Zero-point correction= 1.657485 (Hartree/Particle)
Thermal correction to Energy= 1.804140
Thermal correction to Enthalpy= 1.805259
Thermal correction to Gibbs Free Energy= 1.468119

solvent: -6591.46753605

N	2.578926	-0.116604	-1.313833
C	2.882373	0.580157	-2.461521
C	3.773455	-0.487161	-0.739435
C	4.325430	0.558647	-2.680875
C	3.931340	-1.112859	0.525548
C	2.881609	-1.613073	1.338248
N	1.537949	-1.556456	1.034221
C	0.861942	-1.999598	2.152055
C	1.809268	-2.385974	3.188668

C	3.063964	-2.147988	2.682930
C	-0.543841	-2.002796	2.318371
C	-1.484005	-1.540022	1.363767
N	-1.175345	-0.745974	0.273556
C	-2.370814	-0.234819	-0.197916
C	-2.921743	-1.590442	1.545933
C	-3.475190	-0.788033	0.571583
C	-2.502913	0.752477	-1.212568
C	-1.437464	1.232442	-2.023867
N	-0.126929	0.805451	-1.957193
C	0.565709	1.459957	-2.955868
C	-1.570923	2.236330	-3.070612
C	-0.332303	2.366263	-3.657701
C	1.954208	1.325016	-3.237640
C	2.582877	2.547755	-6.803761
C	3.381768	3.185385	-4.188950
C	2.093757	1.801827	-5.718372
C	3.472921	3.614588	-6.585249
C	3.870666	3.931592	-5.273931
C	2.488194	2.108946	-4.395999
C	-5.697725	2.707844	-0.473074
C	-4.570118	1.258819	-2.600653
C	-6.396853	2.591533	-1.687898
C	-4.439822	2.102535	-0.324423
C	-3.853544	1.372928	-1.387071
C	-5.829923	1.862931	-2.749245
C	-1.039537	-2.278390	3.709641
C	-1.716556	-2.798221	6.407808
C	-1.284714	-3.592750	4.151691
C	-1.157313	-1.196901	4.638111
C	-1.480293	-1.481527	5.985786
C	-1.630707	-3.857838	5.487948
C	5.852118	0.140311	1.533219
C	7.291399	-2.226088	1.995314
C	5.301443	-1.098688	1.129325
C	7.106175	0.194067	2.162099
C	7.829132	-0.989453	2.395761
C	6.035313	-2.281404	1.366649
C	-0.910720	0.209577	4.259295
C	-1.069382	0.870707	3.040441
H	-1.370857	0.550702	2.045304
N	-0.444425	1.129938	5.175899
N	-0.280332	2.274058	4.585436
N	-0.661755	2.146407	3.269693
C	-0.570102	3.225968	2.365299
C	-0.233236	5.352709	0.679683
C	-1.089452	3.125943	1.064024
C	0.261618	5.350621	1.990749
C	-0.924909	4.221644	0.210121
N	0.078147	4.310435	2.832756
Ir	0.308861	4.315385	5.109321

Zn	0.739925	-0.148424	-0.312394
B	3.248539	1.688123	4.206491
B	0.111322	4.169537	7.141819
B	-1.759749	4.672410	5.212833
O	-2.551802	5.503703	6.002817
O	-2.550395	4.027436	4.244908
C	-3.794596	4.763358	4.130655
C	-3.559042	5.845141	3.061413
C	-4.899420	3.800704	3.695342
C	-3.944853	5.349973	5.588796
C	-4.631359	6.713571	5.658114
C	-4.602906	4.361748	6.561805
O	-0.426405	3.018069	7.740700
O	0.424095	5.121055	8.112056
C	-0.688210	3.315590	9.135375
C	0.314365	4.506510	9.414742
C	-0.191485	5.554703	10.408907
C	1.719498	4.026892	9.817467
C	-0.442432	2.054206	9.963792
C	-2.159376	3.751717	9.225812
H	-3.895366	2.195876	0.628302
H	-6.129998	3.276366	0.365884
H	-7.384237	3.065125	-1.806660
H	-6.376590	1.757561	-3.699932
H	-4.130179	0.679348	-3.427336
H	-1.566397	2.204660	0.707294
H	-1.309967	4.163423	-0.819998
H	-0.062656	6.227777	0.036039
H	0.814030	6.198554	2.414226
H	2.102658	3.286100	9.091335
H	1.733537	3.585995	10.835230
H	2.410257	4.892672	9.797620
H	0.575499	6.345405	10.537101
H	-0.389904	5.099188	11.401764
H	-1.116506	6.039600	10.044270
H	-2.476021	3.951077	10.270058
H	-2.795694	2.943378	8.813000
H	-2.325269	4.657900	8.609536
H	-1.191665	1.279971	9.699787
H	0.561345	1.633548	9.765411
H	-0.534152	2.268548	11.049200
H	-2.733867	6.519092	3.368297
H	-4.472583	6.439536	2.856139
H	-3.245863	5.345564	2.121484
H	-5.887124	4.306818	3.691641
H	-4.692747	3.442307	2.665760
H	-4.952876	2.915202	4.356175
H	-5.669734	6.647767	5.271625
H	-4.083401	7.472339	5.068983
H	-4.673738	7.063486	6.709092
H	-5.680508	4.223302	6.339493

H	-4.094984	3.377708	6.521716
H	-4.507534	4.750919	7.594259
H	-2.492587	2.769574	-3.329842
H	-0.049251	3.026566	-4.485323
H	3.686272	3.433483	-3.159748
H	4.563134	4.769378	-5.093755
H	3.856273	4.199080	-7.436613
H	2.269937	2.290743	-7.828345
H	1.398956	0.963960	-5.886125
H	4.844730	0.986733	-3.545671
H	5.608692	-3.247821	1.055131
H	7.855098	-3.155981	2.172428
H	8.811122	-0.949144	2.893217
H	7.512235	1.165794	2.484506
H	5.269666	1.060593	1.367956
H	4.027812	-2.297961	3.181692
H	1.542328	-2.764098	4.181623
H	-1.542641	-0.643349	6.696832
H	-1.972302	-2.996822	7.460555
H	-1.819652	-4.893454	5.812013
H	-1.179436	-4.416849	3.428851
H	-3.435819	-2.163794	2.326594
H	-4.536831	-0.587238	0.388060
O	4.373245	2.082967	4.892996
O	2.530733	0.690680	4.815546
C	4.559275	1.089182	5.966125
C	3.095290	0.510576	6.152274
C	5.553395	0.056037	5.418942
C	5.108159	1.796743	7.199815
C	3.020471	-0.976954	6.492664
C	2.246831	1.337150	7.121943
H	4.478571	2.665631	7.464108
H	6.138916	2.156232	7.005250
H	5.144797	1.097173	8.059957
H	6.487469	0.570513	5.118826
H	5.152070	-0.448660	4.516600
H	5.802939	-0.711160	6.179012
H	3.490736	-1.600780	5.709446
H	1.955257	-1.274403	6.573583
H	3.515264	-1.182238	7.464077
H	2.553756	1.153149	8.171137
H	2.337639	2.420078	6.912231
H	1.178908	1.076806	7.003630
C	4.879103	-0.081811	-1.597581
H	5.938595	-0.281617	-1.398705
N	1.539203	1.526351	0.821409
C	1.967622	2.639495	0.185833
C	1.964076	1.306375	2.078879
C	2.815842	3.571280	0.800186
H	1.602177	2.774678	-0.844661
C	2.828714	2.167123	2.780138

H	1.590360	0.395033	2.566116
C	3.254161	3.333101	2.114585
H	3.899989	4.057232	2.631675
H	1.992153	3.823490	4.895734
B	0.139702	6.373159	5.235488
O	0.512045	7.275493	6.227654
O	-0.486870	7.075066	4.177241
C	-0.301896	8.497936	4.388101
C	0.945123	8.903389	3.584401
C	-1.538893	9.238848	3.880941
C	-0.080449	8.567902	5.949851
C	0.877833	9.667330	6.411417
C	-1.393056	8.629176	6.746818
H	1.822207	8.315062	3.920183
H	1.170655	9.985589	3.670785
H	0.770200	8.676039	2.512503
H	-1.463153	10.325201	4.095002
H	-1.635419	9.107266	2.783901
H	-2.461156	8.850634	4.352138
H	0.484685	10.669040	6.139432
H	1.883041	9.550223	5.964615
H	0.991254	9.627070	7.513223
H	-1.903862	9.607803	6.637862
H	-2.069310	7.811254	6.433022
H	-1.157598	8.467809	7.817590
B	2.344818	5.026315	5.462251
O	3.084821	4.834405	6.632373
O	2.954419	5.986394	4.631087
C	4.227902	6.355243	5.234805
C	5.313618	5.534446	4.519081
C	4.458440	7.851336	5.026978
C	4.034806	5.927866	6.749409
C	5.309285	5.415988	7.422665
C	3.381415	7.013298	7.616709
H	5.151049	4.447557	4.671197
H	6.331531	5.798309	4.871447
H	5.265850	5.752488	3.432096
H	5.390138	8.178424	5.533164
H	4.554659	8.074506	3.944897
H	3.614612	8.442826	5.427068
H	6.065283	6.226373	7.482274
H	5.750042	4.559211	6.883313
H	5.075311	5.087860	8.455611
H	4.060223	7.875914	7.775864
H	2.426793	7.346555	7.169804
H	3.134464	6.572794	8.602299
H	3.117598	4.476575	0.253079

E

Zero-point correction= 1.471858 (Hartree/Particle)

Thermal correction to Energy= 1.603434
Thermal correction to Enthalpy= 1.604552
Thermal correction to Gibbs Free Energy= 1.297365

solvent: -6179.37966031

N	5.288168	1.141379	-0.547069
C	5.316411	2.164375	-1.467638
C	6.289522	1.392499	0.362057
C	6.434157	3.056602	-1.180103
C	6.550530	0.647333	1.541405
C	5.888815	-0.545285	1.935785
N	4.839633	-1.142448	1.269763
C	4.348627	-2.121609	2.111720
C	5.161080	-2.197769	3.315038
C	6.123709	-1.227122	3.203053
C	3.172315	-2.879273	1.893715
C	2.419057	-2.859255	0.692280
N	2.478307	-1.878640	-0.275441
C	1.513865	-2.185134	-1.215798
C	1.400507	-3.833940	0.351879
C	0.851305	-3.428768	-0.847744
C	1.176632	-1.386447	-2.341443
C	1.825038	-0.174871	-2.703289
N	2.916566	0.379819	-2.068165
C	3.242347	1.531391	-2.757030
C	1.413648	0.681832	-3.807443
C	2.284919	1.746133	-3.832444
C	4.356006	2.374161	-2.491123
C	4.967868	4.676690	-5.484927
C	4.318530	4.884513	-2.762050
C	4.835209	3.516190	-4.703924
C	4.775701	5.944428	-4.907021
C	4.451426	6.045098	-3.542173
C	4.507050	3.604332	-3.332102
C	-2.339438	-2.333145	-3.529465
C	0.270300	-2.199180	-4.555867
C	-2.097681	-2.683475	-4.869420
C	-1.279181	-1.917094	-2.706833
C	0.042172	-1.841220	-3.206063
C	-0.788410	-2.615862	-5.379121
C	2.618337	-3.614543	3.084886
C	1.828028	-4.816715	5.531321
C	3.182493	-4.834268	3.511550
C	1.604958	-3.004999	3.896098
C	1.240587	-3.610965	5.118292
C	2.791109	-5.438761	4.718497
C	7.083951	2.450972	3.197055
C	9.551924	1.278101	3.842286
C	7.483922	1.265411	2.536526
C	7.910644	3.044515	4.164132

C	9.146350	2.458315	4.491414
C	8.727885	0.686261	2.869299
C	0.988811	-1.714718	3.515206
C	0.534114	-1.306483	2.264968
H	0.386828	-1.850755	1.332300
N	0.886740	-0.639104	4.377334
N	0.474381	0.402477	3.712125
N	0.244840	0.017254	2.401242
C	0.004148	0.969003	1.386391
C	-0.354767	2.912078	-0.507312
C	-0.283236	0.569060	0.071800
C	-0.067518	3.211706	0.828643
C	-0.450149	1.563775	-0.896760
N	0.142439	2.254968	1.762118
Ir	0.563145	2.639384	3.921392
Zn	3.675890	-0.158213	-0.193736
B	3.568409	1.268506	5.050954
B	0.617904	2.966788	5.948097
B	-1.438582	2.866437	4.167285
O	-2.159570	3.619892	5.090012
O	-2.299519	2.254679	3.240473
C	-3.578829	2.938699	3.318844
C	-3.530635	4.076265	2.283470
C	-4.692270	1.947424	2.979912
C	-3.579219	3.462830	4.812952
C	-4.253054	4.821719	5.011029
C	-4.136721	2.430573	5.804753
O	0.510674	1.967378	6.937746
O	0.625796	4.228652	6.560616
C	0.131153	2.610292	8.187558
C	0.708386	4.065724	7.994652
C	-0.108291	5.171024	8.668206
C	2.195133	4.184253	8.365798
C	0.710586	1.834350	9.368822
C	-1.404602	2.592963	8.244249
H	-1.477739	-1.641392	-1.659430
H	-3.361457	-2.379164	-3.120875
H	-2.928078	-3.010039	-5.515199
H	-0.588180	-2.895569	-6.425737
H	1.296875	-2.151228	-4.951464
H	-0.337001	-0.492969	-0.195641
H	-0.639271	1.280285	-1.942348
H	-0.487495	3.727527	-1.232742
H	-0.000714	4.243602	1.199006
H	2.783550	3.412411	7.833422
H	2.367973	4.098778	9.458543
H	2.564529	5.173713	8.028230
H	0.372535	6.154442	8.492024
H	-0.166920	5.007400	9.764755
H	-1.134228	5.218105	8.257115
H	-1.791956	2.998108	9.201558

H	-1.752690	1.545735	8.137747
H	-1.817174	3.175266	7.398364
H	0.275051	0.815280	9.403332
H	1.807893	1.729374	9.295217
H	0.472470	2.347642	10.323673
H	-2.704195	4.775550	2.521318
H	-4.489040	4.631785	2.229104
H	-3.324122	3.635132	1.286682
H	-5.690289	2.411212	3.124087
H	-4.606440	1.640180	1.917710
H	-4.629446	1.035392	3.603074
H	-5.319941	4.783830	4.707085
H	-3.738771	5.607282	4.426283
H	-4.207804	5.109545	6.081006
H	-5.226559	2.277957	5.668536
H	-3.618677	1.457461	5.689653
H	-3.965399	2.785453	6.839634
H	0.562575	0.497862	-4.473163
H	2.285201	2.596369	-4.523722
H	4.064924	4.958857	-1.692499
H	4.297913	7.034225	-3.081830
H	4.880650	6.853863	-5.519672
H	5.229288	4.590153	-6.551693
H	4.991610	2.522293	-5.151820
H	6.722666	3.928064	-1.778746
H	9.044055	-0.235491	2.355965
H	10.521004	0.816788	4.091230
H	9.793232	2.919597	5.254404
H	7.578549	3.961895	4.675389
H	6.103872	2.891153	2.952550
H	6.908005	-0.973846	3.924335
H	4.994644	-2.890427	4.144609
H	0.480696	-3.116468	5.742881
H	1.527920	-5.273946	6.487230
H	3.249887	-6.390668	5.029423
H	3.965300	-5.294308	2.888396
H	1.159707	-4.724590	0.945082
H	0.078492	-3.934514	-1.437955
O	4.025797	1.981596	6.140965
O	3.662809	-0.096951	5.199313
C	4.728976	0.999438	6.981564
C	3.990408	-0.342829	6.602346
C	6.197332	1.022557	6.528652
C	4.616173	1.395094	8.448678
C	4.855408	-1.597135	6.709870
C	2.660824	-0.542202	7.340039
H	3.562436	1.497299	8.760799
H	5.123203	2.364683	8.624590
H	5.099813	0.627083	9.086675
H	6.586164	2.056045	6.612659
H	6.294028	0.720188	5.466561

H	6.830218	0.358806	7.150999
H	5.756197	-1.540004	6.072745
H	4.262597	-2.481281	6.398631
H	5.172591	-1.749625	7.762176
H	2.823230	-0.782065	8.410696
H	2.010876	0.350079	7.247417
H	2.123126	-1.383615	6.861900
C	7.029718	2.584494	-0.035016
H	7.902710	2.990947	0.488623
N	3.044543	1.326217	1.279043
C	3.058007	2.656676	0.980191
C	3.109752	0.970336	2.568516
C	3.109535	3.639352	1.965946
H	3.031577	2.904364	-0.092150
C	3.185470	1.878795	3.658693
H	3.131411	-0.110179	2.771857
C	3.157825	3.260868	3.333638
H	3.280164	4.025145	4.113912
B	0.536734	4.681678	3.785296
O	1.567325	5.587332	4.091072
O	-0.540603	5.396322	3.212518
C	-0.175672	6.790659	3.061510
C	0.256085	6.992481	1.599226
C	-1.401058	7.649576	3.379406
C	1.021471	6.929834	4.084100
C	2.119471	7.906461	3.659018
C	0.547642	7.239415	5.513265
H	1.147481	6.375168	1.365668
H	0.492840	8.052041	1.373950
H	-0.570537	6.672942	0.932098
H	-1.143161	8.729112	3.378658
H	-2.187773	7.481446	2.615395
H	-1.824315	7.383291	4.365854
H	1.712258	8.932012	3.538691
H	2.594500	7.599399	2.707312
H	2.909904	7.939527	4.435925
H	0.172753	8.278997	5.608336
H	-0.235049	6.522664	5.827556
H	1.402187	7.101327	6.204536
H	3.096698	4.704136	1.692745

CF_3-pyridine

Zero-point correction= 0.090703 (Hartree/Particle)
Thermal correction to Energy= 0.101491
Thermal correction to Enthalpy= 0.102610
Thermal correction to Gibbs Free Energy= 0.047976

solvent: -585.583574

C	14.366971	7.549807	-1.008307

C	12.168164	7.389685	-2.633970
C	14.349545	6.806504	-2.206259
C	12.081780	8.166607	-1.462902
C	13.206758	8.248281	-0.630435
N	13.275328	6.723181	-3.007060
H	11.296178	7.304149	-3.308015
H	15.255120	6.262359	-2.527697
H	11.150797	8.697962	-1.212399
H	13.195729	8.846898	0.293264
C	15.591745	7.554921	-0.121326
F	15.711810	8.725462	0.556797
F	16.730195	7.370889	-0.833828
F	15.532709	6.562334	0.805262

A$_{\mathbf{CF3, anticlockwise}}$

Zero-point correction= 1.308424 (Hartree/Particle)
Thermal correction to Energy= 1.431241
Thermal correction to Enthalpy= 1.432360
Thermal correction to Gibbs Free Energy= 1.141807

solvent: -6105.68923837

N	2.368302	-0.057345	-0.266310
C	2.690092	0.536456	-1.467609
C	3.555156	-0.432214	0.323039
C	4.133656	0.497273	-1.667210
C	3.690463	-0.997942	1.618366
C	2.618734	-1.278763	2.502529
N	1.287934	-0.991904	2.277713
C	0.598553	-1.406858	3.395486
C	1.520096	-1.981163	4.364537
C	2.771564	-1.917985	3.801718
C	-0.796584	-1.283028	3.588725
C	-1.721848	-0.773318	2.642526
N	-1.410572	-0.215851	1.422940
C	-2.597579	0.186943	0.847159
C	-3.160658	-0.733232	2.850018
C	-3.706449	-0.138885	1.733815
C	-2.733046	0.795558	-0.427822
C	-1.669143	1.018617	-1.343282
N	-0.352070	0.649682	-1.156704
C	0.361021	1.165437	-2.220049
C	-1.799404	1.773976	-2.581486
C	-0.538311	1.868503	-3.123094
C	1.773411	1.127328	-2.373022
C	2.690791	2.105974	-5.958889
C	3.117047	3.024150	-3.339177
C	2.147435	1.401585	-4.870963
C	3.448277	3.270761	-5.740391
C	3.660288	3.727495	-4.426765

C	2.353478	1.851791	-3.547923
C	-6.027859	2.732096	-0.462630
C	-4.762836	0.706577	-1.941531
C	-6.679824	2.167444	-1.573714
C	-4.748471	2.284450	-0.095095
C	-4.096101	1.264509	-0.826184
C	-6.042005	1.154027	-2.311749
C	-1.325063	-1.458613	4.981524
C	-2.388684	-1.576689	7.598761
C	-1.626094	-2.699416	5.571029
C	-1.550344	-0.264905	5.725753
C	-2.084079	-0.329011	7.025310
C	-2.154257	-2.758408	6.874314
C	5.574328	-0.434749	3.177611
C	7.240190	-2.373880	2.004614
C	5.079663	-1.232692	2.121346
C	6.893982	-0.597810	3.630111
C	7.730888	-1.565787	3.046458
C	5.923852	-2.206377	1.542193
C	-1.165830	1.006559	5.066537
C	-1.945892	1.953434	4.413440
H	-3.027855	2.041322	4.294185
N	0.158152	1.300347	4.836275
N	0.229212	2.358777	4.098049
N	-1.045425	2.786849	3.801419
C	-1.225880	3.941809	3.013958
C	-1.450755	6.257532	1.563704
C	-2.496796	4.303445	2.533567
C	-0.222840	5.809122	2.069800
C	-2.603466	5.484066	1.787053
N	-0.104934	4.659949	2.774693
Ir	1.813981	3.820918	3.725987
C	1.518916	4.287432	0.048534
C	2.227250	2.353451	1.935982
C	0.741438	3.121799	0.286277
C	2.703680	4.450835	0.739759
C	3.086517	3.499631	1.753946
N	1.099179	2.178910	1.152718
Zn	0.510984	0.139261	0.708677
B	3.088862	2.912571	5.052556
B	1.168918	4.624657	5.520369
B	2.932993	5.564667	3.753943
O	2.511250	6.671643	2.990038
O	4.105386	5.860744	4.425817
C	4.381257	7.279580	4.325036
C	3.926789	7.913293	5.649336
C	5.889146	7.465650	4.136206
C	3.497776	7.734112	3.080288
C	2.755260	9.059732	3.273255
C	4.269652	7.755986	1.751657
O	1.203933	5.954901	5.931037

O	0.424772	3.847914	6.417656
C	0.704092	6.011924	7.301322
C	-0.232466	4.741882	7.351097
C	-0.317022	4.048614	8.710146
C	-1.637925	5.007155	6.783335
C	-0.007801	7.348017	7.506785
C	1.922298	5.885787	8.226971
H	-4.225531	2.730390	0.764646
H	-6.518145	3.530604	0.117520
H	-7.683447	2.516564	-1.863997
H	-6.547876	0.702742	-3.180228
H	-4.264887	-0.091935	-2.513490
H	-3.371244	3.668748	2.730248
H	-3.580198	5.792452	1.384282
H	-1.494704	7.199485	0.997968
H	0.719205	6.370150	1.960026
H	-1.570901	5.515007	5.799091
H	-2.257538	5.626285	7.463290
H	-2.145375	4.032554	6.630613
H	-0.983197	3.164758	8.637891
H	-0.727367	4.732833	9.481483
H	0.677722	3.693234	9.038552
H	1.636271	5.932032	9.297173
H	2.622543	6.716458	8.015231
H	2.460935	4.941063	8.020791
H	0.727491	8.176172	7.451701
H	-0.776241	7.525110	6.730434
H	-0.491728	7.384150	8.504945
H	2.587465	1.450534	2.453892
H	-0.180206	2.935523	-0.286324
H	1.191834	5.012478	-0.710204
H	3.369737	5.299337	0.533964
H	2.845369	7.737340	5.803852
H	4.142881	9.000300	5.688045
H	4.468611	7.415326	6.478025
H	6.141926	8.534350	3.976122
H	6.419190	7.117201	5.045299
H	6.265935	6.873579	3.281567
H	3.467382	9.894777	3.436589
H	2.060976	9.008739	4.132509
H	2.159974	9.291248	2.365789
H	4.999797	8.589291	1.712806
H	4.812198	6.806102	1.592008
H	3.548440	7.891403	0.919206
H	-2.729979	2.200727	-2.972745
H	-0.241159	2.384943	-4.042742
H	3.274811	3.375757	-2.306759
H	4.251675	4.639819	-4.247646
H	3.875150	3.821361	-6.593778
H	2.526429	1.738791	-6.984633
H	1.558243	0.486049	-5.037467

H	4.664640	0.878601	-2.546501
H	5.536369	-2.833199	0.723476
H	7.886833	-3.140554	1.548391
H	8.765807	-1.691263	3.402903
H	7.273398	0.044557	4.441412
H	4.910834	0.316011	3.634332
H	3.720784	-2.275754	4.215176
H	1.237647	-2.387506	5.342651
H	-2.241312	0.602978	7.590708
H	-2.802504	-1.625917	8.618168
H	-2.388968	-3.735774	7.325087
H	-1.452108	-3.620456	4.993273
H	-3.683846	-1.133041	3.727162
H	-4.766626	0.028920	1.510901
O	3.635577	3.508288	6.196893
O	3.291579	1.520438	5.085898
C	4.349883	2.486565	6.942144
C	3.619565	1.177193	6.468131
C	5.819891	2.546430	6.493653
C	4.228155	2.780766	8.436865
C	4.479024	-0.082784	6.519668
C	2.289818	0.932919	7.202149
H	3.169506	2.877013	8.743643
H	4.744065	3.731974	8.679006
H	4.695090	1.970476	9.034646
H	6.189497	3.582774	6.625412
H	5.908499	2.291455	5.418978
H	6.462418	1.861128	7.083679
H	5.395701	0.003244	5.911366
H	3.897997	-0.945268	6.140989
H	4.774089	-0.296331	7.568297
H	2.456455	0.594751	8.245717
H	1.663161	1.847411	7.201661
H	1.727267	0.153513	6.652757
C	4.671043	-0.095466	-0.551657
H	5.728665	-0.269768	-0.327064
C	4.587640	3.347875	1.949750
F	5.223145	4.550958	1.998165
F	5.103625	2.686999	0.869862
F	4.949180	2.649889	3.041837

$A_{CF3, clockwise}$

Zero-point correction= 1.308636 (Hartree/Particle)
Thermal correction to Energy= 1.431630
Thermal correction to Enthalpy= 1.432748
Thermal correction to Gibbs Free Energy= 1.140423

solvent: -6105.70725837

N	2.197271	0.028125	-0.485141

C	2.479390	0.619191	-1.697232
C	3.399025	-0.376442	0.053308
C	3.909488	0.523920	-1.970737
C	3.582775	-0.942085	1.342791
C	2.545446	-1.259027	2.255893
N	1.200149	-1.043765	2.047886
C	0.544977	-1.480733	3.179412
C	1.509803	-1.981088	4.148193
C	2.750901	-1.857786	3.568200
C	-0.855830	-1.470943	3.366426
C	-1.808502	-1.004009	2.424862
N	-1.527559	-0.287696	1.281096
C	-2.738416	0.075935	0.725213
C	-3.245967	-1.126110	2.590800
C	-3.825778	-0.461326	1.531009
C	-2.902725	0.867031	-0.444377
C	-1.839492	1.282519	-1.292410
N	-0.519769	0.890469	-1.178422
C	0.158503	1.445217	-2.246250
C	-1.989177	2.166607	-2.439538
C	-0.750988	2.264583	-3.032306
C	1.544949	1.293429	-2.526007
C	2.067443	2.084787	-6.226756
C	2.994784	2.984168	-3.732714
C	1.600368	1.485644	-5.045267
C	2.998240	3.137379	-6.165086
C	3.459836	3.584183	-4.914087
C	2.057680	1.926610	-3.781639
C	-6.406038	2.419300	-0.344324
C	-4.826984	0.880547	-2.080916
C	-6.920583	2.029193	-1.593138
C	-5.106580	2.041562	0.032521
C	-4.290779	1.270137	-0.829404
C	-6.126008	1.255990	-2.458741
C	-1.361047	-1.756800	4.751162
C	-2.202419	-2.141885	7.421347
C	-1.637334	-3.050302	5.230496
C	-1.517007	-0.641591	5.625900
C	-1.926452	-0.843820	6.956803
C	-2.061607	-3.242309	6.558172
C	5.576555	-0.332117	2.765837
C	7.119333	-2.370615	1.585328
C	4.994565	-1.171395	1.787566
C	6.924104	-0.502138	3.129487
C	7.698583	-1.518066	2.541721
C	5.777308	-2.195272	1.208467
C	-1.170478	0.695339	5.079899
C	-1.855529	1.438571	4.123116
H	-2.837543	1.299078	3.663323
N	0.065717	1.270020	5.277648
N	0.170106	2.301903	4.490702

N	-0.988376	2.431262	3.763493
C	-1.074504	3.437614	2.776812
C	-1.102000	5.441788	0.907693
C	-2.068161	3.406350	1.788337
C	-0.132872	5.377966	1.920728
C	-2.072772	4.426935	0.828331
N	-0.100554	4.375745	2.828706
Ir	1.641740	3.854332	4.149739
C	2.260380	4.038298	0.364166
C	2.190351	2.054566	2.291337
C	1.250640	3.052444	0.402002
C	3.278669	4.000597	1.320334
C	3.241816	3.007842	2.331613
N	1.231729	2.072179	1.313635
Zn	0.381376	0.183719	0.581490
B	3.012498	3.105585	5.456257
B	0.963863	4.894023	5.760763
B	2.824097	5.521795	3.940714
O	2.319514	6.766872	3.535207
O	4.210178	5.604067	4.141098
C	4.572074	7.006977	4.251048
C	4.568795	7.342736	5.752221
C	5.970482	7.203307	3.662474
C	3.406970	7.726566	3.446538
C	2.930119	9.043121	4.065348
C	3.725082	7.921631	1.957166
O	1.533827	6.002996	6.381511
O	-0.264134	4.564115	6.350131
C	0.794042	6.265357	7.602937
C	-0.618366	5.640139	7.263764
C	-1.361354	5.047744	8.460733
C	-1.524265	6.605765	6.481920
C	0.788459	7.774403	7.851372
C	1.519406	5.528666	8.739906
H	-4.715058	2.352306	1.013281
H	-7.018415	3.025966	0.341670
H	-7.939431	2.324328	-1.889774
H	-6.523775	0.935542	-3.434834
H	-4.209399	0.266079	-2.754205
H	-2.774619	2.568669	1.737362
H	-2.816909	4.404544	0.017645
H	-1.061603	6.258488	0.173440
H	0.673364	6.120470	2.029419
H	-0.977602	7.039356	5.620095
H	-1.903464	7.430563	7.119058
H	-2.392559	6.040220	6.087460
H	-2.338220	4.637141	8.133112
H	-1.553777	5.825790	9.228684
H	-0.783604	4.225339	8.922753
H	1.054515	5.731496	9.726242
H	2.573148	5.871322	8.765452

H	1.539149	4.439077	8.546518
H	1.818166	8.118633	8.077790
H	0.434377	8.330110	6.962429
H	0.141955	8.030808	8.716372
H	2.190701	1.184557	2.962783
H	0.448996	3.040550	-0.349822
H	4.102883	4.727989	1.303602
H	3.567397	7.149265	6.184228
H	4.870267	8.392189	5.946582
H	5.286949	6.670355	6.262715
H	6.249564	8.277480	3.655357
H	6.713111	6.658910	4.280226
H	6.039510	6.813141	2.628987
H	3.758136	9.779886	4.124191
H	2.523295	8.874871	5.080124
H	2.123192	9.479600	3.441829
H	4.504828	8.693624	1.797361
H	4.066952	6.976315	1.492876
H	2.805492	8.236016	1.425775
H	-2.919008	2.656273	-2.750531
H	-0.473457	2.852998	-3.913746
H	3.352760	3.342316	-2.757193
H	4.182193	4.413725	-4.853525
H	3.363159	3.609101	-7.091296
H	1.704879	1.723783	-7.202508
H	0.873995	0.658990	-5.090402
H	4.409095	0.874911	-2.880317
H	5.320458	-2.852945	0.452183
H	7.715771	-3.175888	1.127422
H	8.753912	-1.645865	2.830735
H	7.373404	0.170237	3.876225
H	4.969735	0.457913	3.240015
H	3.723211	-2.148908	3.981850
H	1.261867	-2.381482	5.137915
H	-2.024747	0.024866	7.626128
H	-2.525826	-2.294208	8.463036
H	-2.276606	-4.259632	6.921932
H	-1.508096	-3.908503	4.552842
H	-3.742763	-1.665556	3.406261
H	-4.892956	-0.368904	1.298659
O	3.082074	3.398758	6.822126
O	3.930402	2.076010	5.137832
C	4.222455	2.705124	7.384300
C	4.370362	1.487103	6.398823
C	5.412508	3.677510	7.315154
C	3.909423	2.325415	8.831384
C	5.796424	0.960559	6.255436
C	3.395528	0.340604	6.711708
H	2.949417	1.779858	8.902331
H	3.827448	3.241509	9.450918
H	4.714745	1.691124	9.257315

H	5.137102	4.607482	7.852001
H	5.626778	3.950646	6.262209
H	6.326584	3.257424	7.782219
H	6.457875	1.713750	5.785397
H	5.805576	0.047199	5.629194
H	6.212294	0.692939	7.249205
H	3.693586	-0.221560	7.620460
H	2.361686	0.721786	6.832646
H	3.384646	-0.356255	5.850460
H	4.086298	2.869112	3.019072
C	4.482746	-0.072226	-0.874538
H	5.543532	-0.288304	-0.706015
C	2.228900	5.117932	-0.686708
F	1.348774	4.842570	-1.679697
F	3.453931	5.298562	-1.253073
F	1.867860	6.330080	-0.167350

$TS_{AB, CF3}$

Zero-point correction= 1.304143 (Hartree/Particle)
Thermal correction to Energy= 1.426559
Thermal correction to Enthalpy= 1.427678
Thermal correction to Gibbs Free Energy= 1.137404

solvent: -6105.65749522

N	3.036359	-0.501423	-0.713703
C	3.465868	0.049153	-1.902568
C	4.130621	-1.080014	-0.110179
C	4.872749	-0.275357	-2.104525
C	4.148441	-1.683781	1.176680
C	3.008705	-1.945847	1.982982
N	1.710397	-1.616143	1.660368
C	0.910270	-2.049367	2.693396
C	1.733193	-2.683355	3.715176
C	3.036858	-2.605395	3.281576
C	-0.494094	-1.851099	2.786342
C	-1.262952	-1.067168	1.885989
N	-0.740499	-0.275896	0.885848
C	-1.748783	0.552244	0.454931
C	-2.691020	-0.815002	2.005068
C	-2.984548	0.222880	1.148640
C	-1.616880	1.597902	-0.500487
C	-0.541958	1.726536	-1.419830
N	0.556229	0.894684	-1.464543
C	1.379695	1.365834	-2.459761
C	-0.418957	2.759250	-2.441252
C	0.759213	2.516811	-3.108141
C	2.705944	0.923463	-2.726906
C	3.621836	1.914075	-6.302383
C	4.481444	2.449216	-3.681699

C	2.978437	1.302159	-5.213296
C	4.697210	2.793910	-6.084536
C	5.123073	3.061653	-4.770722
C	3.403855	1.556564	-3.889919
C	-3.730564	4.393811	0.964040
C	-3.506949	3.019015	-1.479759
C	-4.579186	4.727839	-0.107772
C	-2.775574	3.376621	0.814221
C	-2.648915	2.673476	-0.411930
C	-4.463848	4.037901	-1.328192
C	-1.196304	-2.415624	3.980368
C	-2.567456	-3.565438	6.192272
C	-1.230979	-3.814724	4.176925
C	-1.857911	-1.582985	4.934669
C	-2.539313	-2.173391	6.023099
C	-1.904697	-4.389162	5.265920
C	6.374865	-0.959128	2.090405
C	7.156261	-3.618394	2.548243
C	5.490717	-2.010762	1.751425
C	7.631429	-1.235101	2.654233
C	8.025611	-2.565474	2.885832
C	5.898229	-3.343168	1.985643
C	-1.872468	-0.112680	4.802316
C	-2.947830	0.757149	4.972105
H	-3.994190	0.575361	5.227057
N	-0.770219	0.624773	4.433188
N	-1.106067	1.877383	4.374224
N	-2.448329	2.001507	4.681437
C	-3.035308	3.272416	4.645118
C	-4.049235	5.826238	4.574966
C	-4.426622	3.445766	4.759386
C	-2.681201	5.568187	4.453810
C	-4.939290	4.745770	4.725631
N	-2.172502	4.311786	4.481657
Ir	-0.041929	3.785705	4.424982
C	3.352830	3.415767	1.550602
C	0.993658	2.146419	2.074506
C	3.003965	2.221539	0.902146
C	1.264860	3.322924	2.802363
C	2.484170	3.975038	2.499529
N	1.839539	1.614108	1.173499
Zn	1.227916	-0.169672	0.225420
B	1.601940	2.810306	5.351263
B	-0.248482	3.915837	6.558901
B	0.760453	5.646167	4.803442
O	-0.023699	6.723751	5.268178
O	2.078677	6.057161	4.639436
C	2.250519	7.359711	5.262321
C	2.751118	7.099331	6.691492
C	3.280861	8.154533	4.460239
C	0.780058	7.937343	5.216911

C	0.401967	8.825304	6.401725
C	0.449973	8.635643	3.888187
O	-1.477528	3.574329	7.133142
O	0.576544	4.538843	7.490705
C	-1.611728	4.359537	8.353563
C	-0.105493	4.539039	8.783077
C	0.204056	5.855151	9.495603
C	0.427415	3.347262	9.586582
C	-2.485382	3.593234	9.344103
C	-2.273341	5.686794	7.943579
H	-2.098293	3.126265	1.648027
H	-3.806609	4.931514	1.922134
H	-5.329883	5.526060	0.007257
H	-5.130816	4.289111	-2.168518
H	-3.429237	2.465742	-2.428757
H	-5.088405	2.575452	4.861171
H	-6.023567	4.912915	4.810819
H	-4.406785	6.865426	4.549999
H	-1.928516	6.366731	4.377544
H	-0.015156	3.308695	10.602439
H	1.525498	3.442849	9.688010
H	0.215333	2.397125	9.058202
H	1.280869	5.900595	9.754528
H	-0.028399	6.725050	8.853444
H	-0.381226	5.939331	10.434726
H	-2.482392	6.335306	8.818348
H	-3.234048	5.462243	7.437048
H	-1.631022	6.236841	7.224457
H	-2.112621	2.563262	9.498774
H	-3.522134	3.526952	8.955735
H	-2.518240	4.113107	10.323967
H	0.070046	1.571845	2.226477
H	3.656062	1.736304	0.160291
H	2.766145	4.884952	3.046386
H	3.686791	6.508768	6.632431
H	2.961015	8.042336	7.236113
H	2.013113	6.495203	7.253997
H	3.368367	9.190378	4.849007
H	4.273096	7.667927	4.544988
H	3.019338	8.196282	3.386033
H	1.059724	9.717528	6.451078
H	0.484071	8.273141	7.356551
H	-0.645659	9.174123	6.295943
H	0.976462	9.606010	3.786551
H	-0.641434	8.828413	3.841584
H	0.721865	7.991666	3.027682
H	-1.132074	3.572389	-2.618553
H	1.194444	3.091519	-3.933849
H	4.797843	2.671902	-2.650379
H	5.956405	3.759611	-4.590876
H	5.200976	3.274337	-6.938455

H	3.282409	1.700023	-7.328561
H	2.135425	0.612878	-5.378650
H	5.466220	-0.008357	-2.986238
H	5.216379	-4.164600	1.714888
H	7.461799	-4.663124	2.719400
H	9.011427	-2.781951	3.327471
H	8.305195	-0.404221	2.917921
H	6.059700	0.081789	1.915500
H	3.936515	-2.952480	3.801970
H	1.367545	-3.105776	4.657928
H	-3.025868	-1.518830	6.764029
H	-3.096400	-4.005109	7.052297
H	-1.917710	-5.483875	5.386947
H	-0.725191	-4.455242	3.437946
H	-3.378664	-1.355788	2.665540
H	-3.956405	0.696496	0.965876
O	1.394228	1.695397	6.159724
O	2.948977	2.995671	5.083995
C	2.694826	1.123555	6.500865
C	3.626760	1.737156	5.382841
C	2.566267	-0.398122	6.463233
C	3.056922	1.612558	7.907900
C	5.057886	2.056119	5.812393
C	3.635407	0.893679	4.096497
H	3.103586	2.719420	7.935535
H	2.275984	1.281745	8.619528
H	4.028954	1.198235	8.243573
H	3.556379	-0.881244	6.597479
H	1.898472	-0.739350	7.280223
H	2.133709	-0.738467	5.504681
H	5.081745	2.789273	6.640691
H	5.587503	1.134082	6.129449
H	5.605032	2.492721	4.952775
H	4.126666	1.471861	3.292156
H	2.609121	0.640382	3.764264
H	4.195068	-0.053323	4.227889
H	-0.127684	4.324145	2.854129
C	5.289274	-0.956928	-0.983600
H	6.288011	-1.355193	-0.769909
C	4.732200	3.980719	1.333874
F	4.826960	5.284394	1.684753
F	5.134119	3.872137	0.037566
F	5.658749	3.298107	2.080121

OMe-pyridine

Zero-point correction= 0.117561 (Hartree/Particle)
Thermal correction to Energy= 0.126930
Thermal correction to Enthalpy= 0.128049
Thermal correction to Gibbs Free Energy= 0.079113

solvent: -362.961502

C	-3.360998	0.229119	-0.009910
C	-1.946561	0.140006	0.003381
C	-1.205839	1.343004	0.008943
C	-1.898459	2.557453	0.001053
C	-3.308666	2.537208	-0.012177
N	-4.021276	1.400636	-0.017484
H	-0.106232	1.290257	0.019337
H	-3.986575	-0.680195	-0.014719
H	-1.352851	3.514407	0.005089
H	-3.882325	3.481686	-0.018745
O	-1.231489	-1.018103	0.011286
C	-1.938636	-2.252245	0.006542
H	-2.569253	-2.366450	-0.904909
H	-1.171569	-3.049686	0.014825
H	-2.586145	-2.363846	0.906370

$A_{OMe, anticlockwise}$

Zero-point correction= 1.335491 (Hartree/Particle)
Thermal correction to Energy= 1.457231
Thermal correction to Enthalpy= 1.458349
Thermal correction to Gibbs Free Energy= 1.168959

solvent: -5883.08501057

N	2.387279	-0.012444	-0.204719
C	2.705005	0.648713	-1.372628
C	3.577059	-0.343780	0.403843
C	4.152063	0.707927	-1.528217
C	3.718655	-0.941527	1.686231
C	2.649314	-1.262538	2.558961
N	1.312723	-1.020315	2.315916
C	0.617667	-1.492727	3.407658
C	1.542797	-2.048179	4.383285
C	2.802679	-1.920850	3.849827
C	-0.786919	-1.456193	3.564001
C	-1.713840	-0.979059	2.603200
N	-1.403424	-0.361466	1.412642
C	-2.596807	-0.012389	0.816039
C	-3.158002	-1.039717	2.765572
C	-3.707706	-0.437255	1.656386
C	-2.737565	0.629283	-0.441641
C	-1.669761	0.919493	-1.332328
N	-0.341888	0.603007	-1.128207
C	0.366383	1.182821	-2.160474
C	-1.811040	1.704032	-2.551036
C	-0.545764	1.869958	-3.063286
C	1.780427	1.214248	-2.286504
C	2.713996	2.246110	-5.850761

C	3.044111	3.184981	-3.224723
C	2.187399	1.515290	-4.772183
C	3.407299	3.447862	-5.619680
C	3.570959	3.914882	-4.302896
C	2.345917	1.975259	-3.445715
C	-6.089879	2.457581	-0.432409
C	-4.751640	0.531464	-1.979140
C	-6.715807	1.916472	-1.569966
C	-4.799871	2.036038	-0.071396
C	-4.111460	1.066292	-0.837524
C	-6.041802	0.952870	-2.341792
C	-1.358453	-1.740876	4.921454
C	-2.551969	-2.091628	7.459018
C	-1.603188	-3.033504	5.419070
C	-1.704970	-0.611834	5.718459
C	-2.303090	-0.793317	6.978334
C	-2.195702	-3.208489	6.683441
C	5.650797	-0.486779	3.250204
C	7.260710	-2.353287	1.888287
C	5.113957	-1.205029	2.156346
C	6.985495	-0.691469	3.642042
C	7.793773	-1.621382	2.964405
C	5.931653	-2.143710	1.485749
C	-1.356076	0.719424	5.161750
C	-2.125353	1.620957	4.434818
H	-3.186958	1.619215	4.177457
N	-0.039795	1.122995	5.108124
N	0.034355	2.204795	4.391634
N	-1.227811	2.538821	3.951168
C	-1.396320	3.728453	3.212734
C	-1.581293	6.128076	1.890055
C	-2.658876	4.137051	2.750421
C	-0.361175	5.634451	2.369675
C	-2.745785	5.358940	2.069361
N	-0.263901	4.444695	3.008531
Ir	1.568152	3.675651	4.058254
C	1.046278	4.255415	-0.045485
C	2.252551	2.426759	1.649855
C	0.391912	3.088284	0.392209
C	2.362696	4.480643	0.343759
C	3.002333	3.546964	1.200667
N	0.991937	2.192288	1.189378
Zn	0.511657	0.092708	0.749916
B	2.882895	2.904636	5.401256
B	0.933463	4.782860	5.609981
B	2.907046	5.207845	3.822419
O	2.599146	6.414612	3.159702
O	4.227014	5.255670	4.294071
C	4.710408	6.619203	4.233775
C	4.515707	7.218831	5.636919
C	6.196819	6.591476	3.868279

C	3.767986	7.276045	3.134569
C	3.316132	8.703147	3.456895
C	4.357679	7.209431	1.717370
O	1.425764	5.994530	6.089998
O	-0.279077	4.449074	6.239047
C	0.657134	6.349001	7.270779
C	-0.713268	5.615041	6.988656
C	-1.464805	5.154711	8.236493
C	-1.637906	6.417310	6.055572
C	0.567265	7.872661	7.354672
C	1.404191	5.773251	8.483922
H	-4.297407	2.464206	0.810196
H	-6.609027	3.217362	0.173915
H	-7.727850	2.245289	-1.854659
H	-6.527599	0.520249	-3.230972
H	-4.224994	-0.228563	-2.577270
H	-3.548606	3.516800	2.924960
H	-3.718240	5.707754	1.690124
H	-1.611892	7.100454	1.377326
H	0.592652	6.180187	2.274722
H	-1.082309	6.764800	5.160639
H	-2.088210	7.295554	6.561426
H	-2.455799	5.753096	5.709075
H	-2.410707	4.654193	7.944607
H	-1.716947	6.018644	8.886229
H	-0.863693	4.432244	8.819843
H	0.920908	6.055269	9.441506
H	2.439927	6.168036	8.478115
H	1.474639	4.671165	8.404692
H	1.572190	8.296391	7.555918
H	0.200471	8.309279	6.406216
H	-0.108595	8.183329	8.178489
H	2.700906	1.625031	2.257570
H	-0.631215	2.848713	0.066112
H	0.523530	4.957301	-0.711247
H	2.932217	5.355642	0.003319
H	3.447779	7.172412	5.924825
H	4.884497	8.262889	5.701077
H	5.081308	6.601636	6.362929
H	6.596218	7.618824	3.738249
H	6.766865	6.101419	4.683623
H	6.375755	6.018990	2.938410
H	4.185732	9.387352	3.543949
H	2.739810	8.733333	4.400612
H	2.662066	9.081627	2.644295
H	5.223370	7.891944	1.596312
H	4.674851	6.176299	1.476747
H	3.576283	7.515158	0.990989
H	-2.752436	2.100938	-2.947652
H	-0.253443	2.423899	-3.962453
H	3.161194	3.546570	-2.190986

H	4.110282	4.856915	-4.113579
H	3.820885	4.019632	-6.465639
H	2.586658	1.871100	-6.878918
H	1.647833	0.571444	-4.948639
H	4.683199	1.154692	-2.376020
H	5.510654	-2.708281	0.638921
H	7.883267	-3.091795	1.358219
H	8.838860	-1.776856	3.276379
H	7.399263	-0.114102	4.482995
H	5.015262	0.241902	3.781501
H	3.755788	-2.245424	4.282304
H	1.256443	-2.481219	5.348561
H	-2.557689	0.088701	7.586581
H	-3.018232	-2.230695	8.447063
H	-2.385978	-4.225343	7.062071
H	-1.335368	-3.903233	4.799115
H	-3.679661	-1.503230	3.611477
H	-4.769227	-0.325106	1.406814
O	2.891387	3.324634	6.744667
O	3.786722	1.828628	5.239165
C	3.995670	2.684448	7.422908
C	4.160128	1.370009	6.577061
C	5.203462	3.630134	7.302234
C	3.621817	2.459852	8.888282
C	5.582416	0.813415	6.563357
C	3.152843	0.277713	6.967391
H	2.646917	1.944147	8.977015
H	3.540895	3.435426	9.409446
H	4.395660	1.856059	9.407063
H	4.925561	4.607492	7.745337
H	5.446163	3.808899	6.235515
H	6.096796	3.243687	7.834048
H	6.273439	1.494991	6.030556
H	5.608112	-0.174955	6.064730
H	5.951490	0.675139	7.601120
H	3.401622	-0.184609	7.944781
H	2.122222	0.684039	6.999398
H	3.165684	-0.507048	6.186703
C	4.694242	0.097506	-0.422578
H	5.755157	-0.036535	-0.183833
C	5.005907	2.846704	2.303900
H	6.035282	3.238868	2.384112
H	4.554049	2.796434	3.315009
H	5.022933	1.841703	1.830247
O	4.297595	3.777731	1.470164

$\mathbf{A_{OMe,\ clockwise}}$

Zero-point correction= 1.335569 (Hartree/Particle)
Thermal correction to Energy= 1.457259
Thermal correction to Enthalpy= 1.458377

Thermal correction to Gibbs Free Energy= 1.169089

solvent: -5883.08598209

N	2.113197	0.015481	-0.536135
C	2.390449	0.684597	-1.708045
C	3.325854	-0.318959	0.026994
C	3.832857	0.742194	-1.918547
C	3.519595	-0.908438	1.304493
C	2.489687	-1.247389	2.218223
N	1.143040	-1.029403	2.024861
C	0.500119	-1.459347	3.166185
C	1.473798	-1.965329	4.123050
C	2.708014	-1.849552	3.527286
C	-0.897465	-1.432845	3.373684
C	-1.858777	-0.965822	2.441458
N	-1.589750	-0.263040	1.286700
C	-2.807474	0.092873	0.740874
C	-3.294456	-1.079483	2.629454
C	-3.886146	-0.425475	1.570046
C	-2.991525	0.843375	-0.450996
C	-1.946310	1.235956	-1.328796
N	-0.607956	0.930934	-1.174346
C	0.048776	1.435004	-2.279878
C	-2.137570	1.993146	-2.557573
C	-0.902426	2.103582	-3.153905
C	1.442505	1.338333	-2.536715
C	3.012202	1.997649	-5.961510
C	1.941167	3.457627	-3.812805
C	2.509061	1.323746	-4.835562
C	2.980965	3.402623	-6.017337
C	2.444326	4.129755	-4.939115
C	1.966632	2.045014	-3.748525
C	-6.476675	2.427802	-0.362398
C	-4.984094	0.707049	-2.003585
C	-7.048770	1.917057	-1.540733
C	-5.162433	2.079562	-0.008356
C	-4.392347	1.217531	-0.823869
C	-6.297761	1.053864	-2.358896
C	-1.394678	-1.699664	4.764669
C	-2.247981	-2.044667	7.436161
C	-1.661525	-2.986966	5.265633
C	-1.564020	-0.570094	5.618321
C	-1.979558	-0.752875	6.950210
C	-2.091488	-3.159249	6.594143
C	5.499824	-0.295138	2.749703
C	7.098103	-2.232826	1.474141
C	4.937162	-1.112276	1.741451
C	6.855399	-0.436673	3.095508
C	7.657214	-1.402400	2.461508
C	5.748509	-2.084319	1.113696

C	-1.206655	0.759232	5.059889
C	-1.865955	1.502705	4.084994
H	-2.841801	1.372700	3.609549
N	0.031511	1.322396	5.277085
N	0.162703	2.347272	4.485368
N	-0.980715	2.484032	3.734531
C	-1.023844	3.473204	2.727510
C	-0.956007	5.431213	0.810373
C	-1.986365	3.434608	1.707984
C	-0.015332	5.370659	1.849692
C	-1.944918	4.433856	0.726859
N	-0.034685	4.396052	2.788265
Ir	1.660189	3.870812	4.168196
C	2.321383	3.971285	0.253175
C	2.204872	2.059306	2.264418
C	1.276399	3.018885	0.353718
C	3.352354	3.934725	1.197990
C	3.273765	2.985111	2.250475
N	1.237303	2.074065	1.296106
Zn	0.315718	0.213138	0.573197
B	3.010898	3.097316	5.472514
B	0.978527	4.929065	5.754429
B	2.901642	5.486618	3.962859
O	2.450934	6.748247	3.528154
O	4.281672	5.543178	4.226384
C	4.669629	6.933900	4.362281
C	4.610602	7.266169	5.863194
C	6.098391	7.102337	3.838929
C	3.556562	7.685439	3.512843
C	3.074401	9.004403	4.122521
C	3.957842	7.889706	2.044202
O	1.551314	6.041823	6.372417
O	-0.252559	4.610918	6.348902
C	0.819955	6.304773	7.597538
C	-0.596738	5.685818	7.264786
C	-1.335656	5.095139	8.465498
C	-1.503508	6.657533	6.491065
C	0.821911	7.813390	7.849983
C	1.548347	5.562964	8.729651
H	-4.722096	2.483516	0.916525
H	-7.055491	3.105168	0.285544
H	-8.079196	2.188675	-1.819443
H	-6.741074	0.640485	-3.278854
H	-4.398888	0.023207	-2.637957
H	-2.703106	2.605535	1.650637
H	-2.664576	4.406477	-0.105508
H	-0.882933	6.229399	0.058194
H	0.804579	6.098652	1.965144
H	-0.959914	7.091157	5.627338
H	-1.876055	7.482243	7.132426
H	-2.376021	6.096256	6.099761

H	-2.316183	4.689267	8.142807
H	-1.520162	5.872541	9.236150
H	-0.758789	4.269162	8.922252
H	1.088299	5.763601	9.718788
H	2.603109	5.902995	8.751935
H	1.565585	4.474033	8.532049
H	1.854483	8.153083	8.070562
H	0.463950	8.373006	6.964983
H	0.182200	8.070270	8.719943
H	2.185549	1.219865	2.972662
H	0.472028	3.021009	-0.395714
H	4.202084	4.629761	1.168270
H	3.588111	7.093151	6.251312
H	4.927330	8.308155	6.073443
H	5.291505	6.576541	6.400536
H	6.402082	8.169884	3.849628
H	6.798522	6.539378	4.488739
H	6.209453	6.711869	2.809123
H	3.911503	9.724768	4.233608
H	2.613650	8.829337	5.112701
H	2.307023	9.463890	3.466099
H	4.750040	8.656462	1.925730
H	4.323629	6.938076	1.610652
H	3.066941	8.213472	1.467868
H	-3.094825	2.378317	-2.926542
H	-0.658185	2.583119	-4.108442
H	1.541296	4.018779	-2.954250
H	2.421117	5.231131	-4.974045
H	3.375107	3.930241	-6.900456
H	3.427850	1.420785	-6.803136
H	2.529990	0.223545	-4.788875
H	4.332383	1.221562	-2.767983
H	5.307219	-2.721937	0.331427
H	7.716294	-2.998550	0.978693
H	8.718412	-1.507694	2.738044
H	7.289575	0.219721	3.864819
H	4.875029	0.459431	3.257269
H	3.683751	-2.144860	3.929703
H	1.235738	-2.361132	5.117065
H	-2.086839	0.126479	7.604027
H	-2.576617	-2.181026	8.478453
H	-2.299641	-4.171815	6.974819
H	-1.522567	-3.855654	4.603409
H	-3.781767	-1.606697	3.458402
H	-4.955815	-0.327645	1.351603
O	3.083121	3.378955	6.842067
O	3.914258	2.055139	5.147711
C	4.204958	2.656663	7.402648
C	4.334891	1.446168	6.405041
C	5.415551	3.604758	7.352304
C	3.875060	2.267552	8.843730

C	5.751681	0.893563	6.266863
C	3.336952	0.314972	6.701376
H	2.903939	1.740473	8.902174
H	3.806494	3.178636	9.472403
H	4.664785	1.613083	9.268533
H	5.156604	4.532080	7.901894
H	5.638492	3.888555	6.303999
H	6.318987	3.159762	7.817214
H	6.430879	1.639631	5.810928
H	5.748989	-0.012766	5.630273
H	6.154413	0.607091	7.260863
H	3.618044	-0.261189	7.606864
H	2.309475	0.714225	6.818471
H	3.319287	-0.373403	5.833464
H	4.102791	2.870211	2.961510
C	4.413723	0.122577	-0.839734
H	5.482053	-0.004084	-0.632072
O	2.208994	4.841808	-0.788598
C	3.342330	5.639199	-1.120949
H	3.084407	6.169669	-2.056825
H	3.565133	6.384138	-0.326803
H	4.241780	5.008085	-1.298631

$TS_{AB,\ OMe}$

Zero-point correction= 1.330837 (Hartree/Particle)
Thermal correction to Energy= 1.451902
Thermal correction to Enthalpy= 1.453021
Thermal correction to Gibbs Free Energy= 1.166945

solvent: -5883.03517859

N	3.214973	-0.685399	-0.392579
C	3.687476	-0.204006	-1.596789
C	4.184582	-1.513302	0.128166
C	4.985605	-0.802200	-1.879188
C	4.117893	-2.199980	1.373346
C	2.965005	-2.295819	2.199620
N	1.726438	-1.772130	1.900817
C	0.868130	-2.148881	2.910333
C	1.602667	-2.905606	3.915511
C	2.903246	-2.996362	3.475384
C	-0.523014	-1.858066	2.954218
C	-1.205347	-1.037166	2.017148
N	-0.592741	-0.249053	1.069858
C	-1.542477	0.619742	0.591239
C	-2.631721	-0.748040	2.041796
C	-2.835812	0.308982	1.184693
C	-1.308434	1.691976	-0.314876
C	-0.162318	1.808561	-1.147149
N	0.864291	0.888143	-1.204103

C	1.767990	1.350575	-2.129460
C	0.109585	2.906747	-2.064334
C	1.294860	2.608676	-2.697265
C	3.047950	0.781928	-2.399236
C	3.956963	1.916214	-5.931653
C	5.082003	1.946896	-3.361045
C	3.258901	1.349559	-4.852949
C	5.219972	2.501528	-5.729378
C	5.779922	2.514769	-4.439084
C	3.809697	1.353616	-3.548859
C	-3.579990	4.428443	1.029118
C	-3.006384	3.228514	-1.449384
C	-4.248161	4.863363	-0.130391
C	-2.630049	3.399155	0.948168
C	-2.324129	2.784065	-0.293260
C	-3.958592	4.259083	-1.367640
C	-1.317373	-2.406102	4.096756
C	-2.843396	-3.549380	6.212317
C	-1.396079	-3.806727	4.278216
C	-2.028546	-1.567853	5.011981
C	-2.780964	-2.157743	6.054179
C	-2.144910	-4.378358	5.317742
C	6.531936	-2.138070	2.106355
C	6.580640	-4.924109	2.447755
C	5.362576	-2.887417	1.836034
C	7.706899	-2.771405	2.543371
C	7.735207	-4.166991	2.716467
C	5.405484	-4.290356	2.011226
C	-2.042974	-0.096580	4.887767
C	-3.121160	0.766511	5.071586
H	-4.163719	0.578236	5.336998
N	-0.951818	0.653589	4.510532
N	-1.293975	1.904043	4.455375
N	-2.632563	2.014358	4.780091
C	-3.242658	3.277398	4.773259
C	-4.307033	5.810067	4.765514
C	-4.629669	3.423007	4.959615
C	-2.942625	5.576198	4.574545
C	-5.168354	4.713117	4.955163
N	-2.409463	4.329704	4.568041
Ir	-0.263715	3.876202	4.406509
C	2.746340	3.835966	1.055322
C	1.069657	2.078536	2.355401
C	2.763901	2.455889	0.784407
C	1.003233	3.448437	2.725982
C	1.864211	4.329118	2.040337
N	1.923723	1.619572	1.432582
Zn	1.423962	-0.223349	0.534869
B	1.570360	3.134012	5.107630
B	-0.327197	3.948074	6.542964
B	0.432973	5.778086	4.701740

O	-0.294640	6.725240	5.463191
O	1.616196	6.356060	4.248119
C	1.868878	7.544307	5.043043
C	2.670784	7.086815	6.273685
C	2.668878	8.541037	4.205875
C	0.402990	8.000796	5.404853
C	0.264010	8.699310	6.756720
C	-0.259404	8.829751	4.293040
O	-1.512290	3.567524	7.188078
O	0.578878	4.478673	7.455116
C	-1.532703	4.242151	8.479583
C	0.007827	4.375739	8.792333
C	0.395164	5.625986	9.581373
C	0.599524	3.117913	9.443615
C	-2.326159	3.395621	9.472522
C	-2.212341	5.602211	8.241247
H	-2.092217	3.070339	1.851212
H	-3.792489	4.896194	2.003624
H	-4.994929	5.671284	-0.070874
H	-4.486723	4.586450	-2.277613
H	-2.797521	2.741372	-2.414613
H	-5.270049	2.541811	5.097743
H	-6.249734	4.859531	5.097428
H	-4.684113	6.842687	4.762200
H	-2.214981	6.391481	4.460823
H	0.255821	2.993912	10.490528
H	1.704196	3.205109	9.449300
H	0.332135	2.211962	8.864363
H	1.492773	5.648575	9.735189
H	0.109443	6.546251	9.038907
H	-0.095682	5.631570	10.576736
H	-2.335092	6.178417	9.180776
H	-3.217197	5.422883	7.806422
H	-1.631085	6.202432	7.510778
H	-1.950419	2.355601	9.509366
H	-3.392709	3.363291	9.169770
H	-2.271796	3.831403	10.491765
H	0.439624	1.315614	2.830249
H	3.416750	1.990177	0.034774
H	1.875873	5.401680	2.274909
H	3.569799	6.545177	5.920880
H	2.985863	7.943448	6.904367
H	2.081050	6.369102	6.878933
H	2.787157	9.503093	4.746750
H	3.679111	8.131015	4.005569
H	2.184350	8.736727	3.230257
H	0.865516	9.631578	6.782222
H	0.599417	8.040012	7.578651
H	-0.796788	8.966619	6.941037
H	0.175059	9.847136	4.216741
H	-1.341523	8.934222	4.515105

H	-0.153296	8.322974	3.312567
H	-0.512205	3.799076	-2.198327
H	1.820630	3.208254	-3.449496
H	5.515497	1.966441	-2.349066
H	6.765464	2.977211	-4.269012
H	5.766754	2.947035	-6.575591
H	3.513091	1.896262	-6.939890
H	2.271593	0.887879	-5.010069
H	5.572729	-0.647207	-2.791471
H	4.503449	-4.881973	1.789755
H	6.596966	-6.018819	2.571964
H	8.656699	-4.664423	3.058655
H	8.605688	-2.170146	2.754970
H	6.503471	-1.044912	1.975187
H	3.748301	-3.476644	3.981057
H	1.186182	-3.299652	4.849397
H	-3.300945	-1.501906	6.770886
H	-3.429386	-3.984414	7.036994
H	-2.187727	-5.473760	5.424735
H	-0.862612	-4.451793	3.563282
H	-3.378351	-1.280343	2.642194
H	-3.780549	0.807964	0.939116
O	1.690236	1.766924	5.295820
O	2.769143	3.795739	5.294578
C	3.054677	1.449139	5.673686
C	3.833294	2.807448	5.360325
C	3.495084	0.242760	4.838217
C	3.025002	1.093498	7.166105
C	4.821296	3.247818	6.443445
C	4.520178	2.799082	3.987575
H	2.662768	1.952883	7.761938
H	2.319260	0.252310	7.318311
H	4.022693	0.788592	7.541653
H	4.563687	-0.001720	5.008617
H	2.888985	-0.642377	5.113554
H	3.334074	0.415342	3.756912
H	4.309812	3.423182	7.408516
H	5.616097	2.487778	6.590059
H	5.303521	4.198734	6.138244
H	4.835941	3.830792	3.734648
H	3.825129	2.461106	3.198271
H	5.409562	2.137192	3.977947
H	-0.441817	4.167589	2.763375
C	5.298638	-1.603575	-0.805348
H	6.191983	-2.223782	-0.668505
O	3.536111	4.746692	0.419274
C	4.319347	4.283257	-0.675415
H	4.812214	5.174125	-1.108674
H	5.104137	3.562132	-0.347837
H	3.692218	3.794146	-1.455669

Me-pyridine

Zero-point correction= 0.112468 (Hartree/Particle)
Thermal correction to Energy= 0.120899
Thermal correction to Enthalpy= 0.122018
Thermal correction to Gibbs Free Energy= 0.074876

solvent: -287.723305

C	-1.574350	-1.039801	3.229682
C	-1.660148	0.181064	3.932168
C	-1.754097	0.095601	5.339994
N	-1.766686	-1.052774	6.037828
C	-1.683908	-2.200884	5.344590
C	-1.586065	-2.248399	3.941081
H	-1.498661	-1.039314	2.129121
H	-1.823467	1.027674	5.933916
H	-1.695681	-3.135693	5.935141
H	-1.520478	-3.215887	3.418534
C	-1.652920	1.510480	3.216894
H	-0.724324	1.642588	2.621384
H	-2.505341	1.591285	2.508795
H	-1.722599	2.357799	3.928252

$A_{Me, anticlockwise}$

Zero-point correction= 1.330707 (Hartree/Particle)
Thermal correction to Energy= 1.451125
Thermal correction to Enthalpy= 1.452244
Thermal correction to Gibbs Free Energy= 1.165935

solvent: -5807.84325106

N	2.219973	-0.028926	-0.330829
C	2.531728	0.590365	-1.521198
C	3.409523	-0.431707	0.233728
C	3.971254	0.530608	-1.747793
C	3.565079	-1.005306	1.524262
C	2.510300	-1.260225	2.438175
N	1.176336	-0.986348	2.219332
C	0.495075	-1.401400	3.342899
C	1.429259	-1.938095	4.321227
C	2.678652	-1.865961	3.753687
C	-0.905713	-1.348592	3.518925
C	-1.841470	-0.871516	2.566571
N	-1.539490	-0.211242	1.395692
C	-2.739653	0.154515	0.818603
C	-3.282102	-0.944409	2.736760
C	-3.842365	-0.312853	1.647524
C	-2.884518	0.865298	-0.403151
C	-1.809494	1.199505	-1.272877
N	-0.494737	0.806379	-1.119593

C	0.211024	1.345294	-2.176597
C	-1.936641	2.035425	-2.458665
C	-0.683184	2.124916	-3.019304
C	1.613607	1.244505	-2.381624
C	2.414324	2.229902	-5.991123
C	3.008842	3.090627	-3.385038
C	1.888862	1.538722	-4.886248
C	3.237980	3.353256	-5.796498
C	3.533888	3.781426	-4.489604
C	2.179395	1.959923	-3.569197
C	-6.365095	2.473237	-0.427842
C	-4.796245	0.808046	-2.053073
C	-6.876167	2.015189	-1.654921
C	-5.074104	2.100713	-0.018427
C	-4.262729	1.265871	-0.823559
C	-6.086406	1.178959	-2.464477
C	-1.428755	-1.578330	4.907046
C	-2.347899	-1.815912	7.567078
C	-1.749512	-2.841168	5.436272
C	-1.573905	-0.420949	5.725763
C	-2.026223	-0.547490	7.051218
C	-2.210774	-2.959107	6.760648
C	5.546881	-0.595757	3.031409
C	7.034997	-2.591680	1.720795
C	4.958650	-1.318031	1.968211
C	6.867712	-0.866670	3.426757
C	7.616171	-1.861693	2.773148
C	5.715909	-2.321157	1.321001
C	-1.178458	0.872467	5.110843
C	-1.891418	1.655122	4.208500
H	-2.921756	1.591851	3.848838
N	0.121089	1.324846	5.152443
N	0.237089	2.326621	4.331638
N	-0.977148	2.550548	3.726243
C	-1.095079	3.590343	2.781292
C	-1.247004	5.715500	1.060615
C	-2.169047	3.634682	1.880419
C	-0.177998	5.556901	1.956719
C	-2.242547	4.723285	1.000875
N	-0.091092	4.499043	2.796275
Ir	1.701714	3.897400	4.030387
C	1.879934	4.192458	0.111563
C	2.307105	2.215045	1.998020
C	0.968222	3.140193	0.320140
C	3.057670	4.209658	0.853846
C	3.314361	3.202258	1.827427
N	1.181223	2.170332	1.221161
Zn	0.387096	0.203089	0.701333
B	3.041607	3.102341	5.336669
B	0.990421	4.812150	5.689281
B	2.884821	5.560422	3.880041

O	2.457604	6.758045	3.264767
O	4.204472	5.717319	4.334730
C	4.528984	7.129492	4.350201
C	4.207862	7.645432	5.763108
C	6.019619	7.298443	4.047974
C	3.554153	7.708958	3.243647
C	2.997364	9.101518	3.546718
C	4.166447	7.673620	1.834178
O	1.282534	6.094938	6.153838
O	-0.008200	4.214748	6.472403
C	0.665088	6.250303	7.460767
C	-0.534758	5.223928	7.372613
C	-0.906636	4.557999	8.697390
C	-1.780385	5.817538	6.692801
C	0.252203	7.712189	7.634780
C	1.717493	5.852145	8.506967
H	-4.685222	2.463113	0.945339
H	-6.973861	3.128697	0.215252
H	-7.888342	2.306000	-1.977592
H	-6.481509	0.804660	-3.422357
H	-4.183476	0.143695	-2.681940
H	-2.890603	2.808057	1.840890
H	-3.062832	4.777050	0.268824
H	-1.274120	6.592295	0.397286
H	0.660290	6.269835	2.036002
H	-1.504626	6.307452	5.736892
H	-2.299286	6.555910	7.337485
H	-2.486409	4.993524	6.464030
H	-1.755197	3.860494	8.543202
H	-1.213342	5.315716	9.448319
H	-0.057493	3.976246	9.102430
H	1.348072	5.996974	9.542613
H	2.617771	6.481557	8.360103
H	2.028439	4.800769	8.357863
H	1.156895	8.351702	7.685789
H	-0.363776	8.065130	6.786002
H	-0.320054	7.850444	8.575752
H	2.468387	1.357858	2.669787
H	0.036244	3.070536	-0.259880
H	1.659533	4.964289	-0.640029
H	3.812646	4.992002	0.685746
H	3.133718	7.498866	5.987092
H	4.482238	8.712724	5.890010
H	4.782846	7.041596	6.493761
H	6.287814	8.372063	3.961263
H	6.615791	6.859794	4.873555
H	6.311298	6.783063	3.112898
H	3.816717	9.843068	3.651230
H	2.399493	9.093975	4.477139
H	2.336843	9.433054	2.719045
H	4.945067	8.451324	1.697524

H	4.623587	6.684617	1.632522
H	3.364708	7.843127	1.086725
H	-2.860928	2.507672	-2.810294
H	-0.388461	2.681533	-3.915980
H	3.231702	3.421448	-2.358086
H	4.176085	4.662295	-4.329028
H	3.650138	3.894163	-6.663157
H	2.183041	1.885141	-7.011687
H	1.246719	0.656278	-5.034506
H	4.493712	0.918861	-2.629231
H	5.255315	-2.890458	0.498258
H	7.610824	-3.379812	1.209704
H	8.651777	-2.070059	3.085815
H	7.316394	-0.292720	4.252157
H	4.956998	0.181412	3.543492
H	3.631829	-2.198891	4.179297
H	1.154539	-2.330126	5.307182
H	-2.117750	0.353958	7.676778
H	-2.702846	-1.911682	8.605266
H	-2.460903	-3.952424	7.166039
H	-1.630712	-3.732615	4.800945
H	-3.794248	-1.434817	3.573386
H	-4.906462	-0.206218	1.407319
O	3.302629	3.597316	6.623720
O	3.702313	1.861334	5.164610
C	4.322295	2.776159	7.239676
C	4.131326	1.408647	6.483337
C	5.673586	3.446191	6.936519
C	4.072285	2.711948	8.746660
C	5.406553	0.578325	6.357605
C	2.984754	0.559119	7.052886
H	3.032211	2.404735	8.966231
H	4.236509	3.710634	9.199888
H	4.766319	1.994443	9.232422
H	5.637219	4.491705	7.302640
H	5.848473	3.486355	5.842800
H	6.521279	2.926469	7.428373
H	6.170386	1.106055	5.755044
H	5.189742	-0.392466	5.869616
H	5.832135	0.367797	7.360973
H	3.254628	0.097356	8.025002
H	2.066383	1.167288	7.170240
H	2.747458	-0.241961	6.324895
C	4.517558	-0.093395	-0.652974
H	5.575558	-0.298295	-0.455638
C	4.692703	3.063634	2.422491
H	5.050297	4.036013	2.815030
H	4.697565	2.346011	3.260902
H	5.391298	2.704051	1.635894

$A_{Me, clockwise}$

Zero-point correction= 1.330678 (Hartree/Particle)
Thermal correction to Energy= 1.451134
Thermal correction to Enthalpy= 1.452253
Thermal correction to Gibbs Free Energy= 1.165558

solvent: -5807.84722767

N	2.177034	-0.006147	-0.493150
C	2.465798	0.591292	-1.699525
C	3.373953	-0.428658	0.041689
C	3.893943	0.474721	-1.978101
C	3.554036	-0.988870	1.333999
C	2.514420	-1.289257	2.249864
N	1.171350	-1.066909	2.038440
C	0.512280	-1.496943	3.169930
C	1.472611	-1.996234	4.144290
C	2.715280	-1.880937	3.566594
C	-0.889174	-1.486968	3.350959
C	-1.836497	-1.019638	2.404384
N	-1.548862	-0.298859	1.265014
C	-2.756775	0.068445	0.705425
C	-3.274864	-1.140966	2.563180
C	-3.848887	-0.470679	1.503831
C	-2.915457	0.867031	-0.459786
C	-1.848637	1.283655	-1.302940
N	-0.531162	0.886205	-1.188558
C	0.154244	1.451061	-2.245294
C	-1.991944	2.179437	-2.441369
C	-0.750004	2.282362	-3.026582
C	1.542271	1.294950	-2.516534
C	2.131944	2.292836	-6.161569
C	3.033402	3.020592	-3.605113
C	1.637440	1.628150	-5.026995
C	3.076983	3.325152	-6.021745
C	3.527268	3.685935	-4.739023
C	2.079186	1.983725	-3.731574
C	-6.404353	2.448185	-0.345005
C	-4.841704	0.907167	-2.094524
C	-6.924301	2.070988	-1.595602
C	-5.107721	2.056507	0.027317
C	-4.300401	1.283421	-0.841186
C	-6.137956	1.296702	-2.467777
C	-1.397413	-1.777955	4.733712
C	-2.221498	-2.181467	7.406407
C	-1.687094	-3.072936	5.201111
C	-1.533358	-0.670008	5.620966
C	-1.932961	-0.882127	6.953508
C	-2.103490	-3.274066	6.529905
C	5.548212	-0.386750	2.758707
C	7.080628	-2.440583	1.591604

C	4.963573	-1.225751	1.781619
C	6.892957	-0.565155	3.128315
C	7.662345	-1.588848	2.547233
C	5.741181	-2.257254	1.209212
C	-1.179511	0.669081	5.086566
C	-1.848587	1.406748	4.114879
H	-2.820670	1.261858	3.636314
N	0.046146	1.255173	5.315606
N	0.161130	2.290032	4.532498
N	-0.982431	2.407758	3.776410
C	-1.056993	3.410095	2.786610
C	-1.082759	5.420133	0.925629
C	-2.039296	3.373351	1.786265
C	-0.117066	5.354874	1.941038
C	-2.045331	4.397846	0.831682
N	-0.087833	4.352759	2.850235
Ir	1.626056	3.851222	4.208133
C	2.297300	4.029553	0.352332
C	2.177591	2.015302	2.283072
C	1.277735	3.050002	0.398348
C	3.305788	3.938524	1.320129
C	3.242363	2.944437	2.326878
N	1.229296	2.057650	1.302200
Zn	0.365892	0.188561	0.581092
B	3.005355	3.106093	5.504367
B	0.969594	4.914136	5.801834
B	2.803249	5.503753	3.940645
O	2.278032	6.746242	3.531135
O	4.196697	5.601747	4.082392
C	4.552225	7.007301	4.143414
C	4.587222	7.384212	5.634744
C	5.930285	7.198965	3.507460
C	3.358873	7.696482	3.357267
C	2.914931	9.043563	3.932993
C	3.612863	7.818786	1.845986
O	1.570710	6.012573	6.417736
O	-0.264960	4.620289	6.399861
C	0.844822	6.296157	7.641354
C	-0.585455	5.707444	7.311115
C	-1.338281	5.139504	8.514013
C	-1.469371	6.695372	6.531499
C	0.879441	7.805412	7.887672
C	1.555538	5.543469	8.777447
H	-4.711074	2.357381	1.009128
H	-7.010324	3.055568	0.346047
H	-7.941077	2.376888	-1.888492
H	-6.540240	0.986257	-3.445280
H	-4.230515	0.291657	-2.772723
H	-2.738767	2.530548	1.725479
H	-2.784493	4.374872	0.016503
H	-1.055964	6.248119	0.202472

H	0.682809	6.103523	2.067222
H	-0.915122	7.112121	5.666196
H	-1.823716	7.531449	7.168408
H	-2.353983	6.151983	6.142139
H	-2.327583	4.754400	8.192669
H	-1.505583	5.924836	9.280526
H	-0.780735	4.303056	8.975691
H	1.099739	5.759875	9.765243
H	2.617812	5.858981	8.798086
H	1.547472	4.453339	8.586003
H	1.918573	8.123082	8.110067
H	0.536797	8.368607	6.998849
H	0.242772	8.080287	8.754369
H	2.143571	1.156909	2.968432
H	0.471353	3.053653	-0.351434
H	4.148024	4.647270	1.326830
H	3.596625	7.202641	6.096911
H	4.894305	8.437984	5.794601
H	5.316874	6.723275	6.144298
H	6.198197	8.275124	3.459843
H	6.697063	6.680165	4.117618
H	5.972165	6.777656	2.484526
H	3.749096	9.775843	3.925271
H	2.549092	8.924449	4.970006
H	2.085152	9.458688	3.324780
H	4.388326	8.575060	1.608641
H	3.924982	6.845919	1.418274
H	2.669370	8.121080	1.346528
H	-2.920000	2.671963	-2.753333
H	-0.471987	2.874929	-3.905353
H	3.382766	3.298679	-2.599215
H	4.266290	4.494337	-4.619518
H	3.463943	3.846198	-6.911805
H	1.780734	1.997947	-7.163333
H	0.901702	0.815800	-5.134706
H	4.397423	0.821874	-2.887131
H	5.282193	-2.915122	0.454366
H	7.672912	-3.252016	1.139147
H	8.715517	-1.723471	2.841051
H	7.343865	0.106767	3.874533
H	4.945442	0.409907	3.226580
H	3.685253	-2.174096	3.984214
H	1.220647	-2.391046	5.135231
H	-2.014656	-0.019479	7.632784
H	-2.537385	-2.341020	8.449341
H	-2.328749	-4.292474	6.884339
H	-1.572422	-3.925512	4.513782
H	-3.775676	-1.682892	3.374539
H	-4.914857	-0.374925	1.267234
O	3.095302	3.394730	6.871107
O	3.918340	2.074411	5.172367

C	4.239735	2.695021	7.415527
C	4.369915	1.479387	6.424357
C	5.433347	3.662558	7.332477
C	3.944900	2.312925	8.865834
C	5.791503	0.945960	6.260719
C	3.393698	0.335568	6.745134
H	2.984664	1.769266	8.947799
H	3.872275	3.228038	9.488097
H	4.754271	1.676068	9.280263
H	5.167858	4.594587	7.870928
H	5.637217	3.932954	6.276707
H	6.351187	3.239219	7.789382
H	6.451110	1.698091	5.786358
H	5.787171	0.036429	5.628683
H	6.218625	0.669773	7.247408
H	3.700134	-0.232089	7.647784
H	2.363345	0.721295	6.880704
H	3.368542	-0.357522	5.880729
H	4.063375	2.805215	3.042340
C	4.460207	-0.138446	-0.887960
H	5.517692	-0.373096	-0.723605
C	2.256934	5.130629	-0.676772
H	3.272727	5.386777	-1.040994
H	1.632991	4.851178	-1.549276
H	1.830223	6.058247	-0.236801

$TS_{AB, Me}$

Zero-point correction= 1.326153 (Hartree/Particle)
Thermal correction to Energy= 1.446041
Thermal correction to Enthalpy= 1.447159
Thermal correction to Gibbs Free Energy= 1.162455

solvent: -5807.79356297

N	3.195856	-0.440537	-0.651303
C	3.592270	0.043839	-1.880583
C	4.245740	-1.167492	-0.137023
C	4.928073	-0.452459	-2.186205
C	4.268502	-1.818681	1.128020
C	3.149281	-1.984300	1.988438
N	1.865358	-1.566848	1.714883
C	1.069291	-1.981753	2.759488
C	1.887592	-2.660723	3.755731
C	3.179310	-2.655722	3.281217
C	-0.334260	-1.775227	2.856259
C	-1.101311	-0.999909	1.946174
N	-0.574301	-0.201961	0.955126
C	-1.585161	0.616715	0.513689
C	-2.534832	-0.767572	2.043935
C	-2.828343	0.268565	1.185885

C	-1.448669	1.673383	-0.428961
C	-0.361331	1.819443	-1.330702
N	0.727834	0.977107	-1.387329
C	1.550705	1.447002	-2.381150
C	-0.223169	2.871059	-2.330365
C	0.950393	2.622962	-3.003917
C	2.851800	0.952592	-2.686925
C	3.531325	1.872599	-6.330759
C	4.730809	2.223788	-3.817075
C	2.922862	1.341752	-5.181659
C	4.742429	2.580339	-6.227451
C	5.339164	2.755307	-4.965706
C	3.514755	1.505128	-3.906606
C	-3.655395	4.412087	1.006310
C	-3.325926	3.079291	-1.448120
C	-4.473408	4.748040	-0.088410
C	-2.677941	3.413876	0.874605
C	-2.498468	2.731845	-0.356643
C	-4.305026	4.079209	-1.314442
C	-1.043584	-2.353821	4.039378
C	-2.421429	-3.542414	6.229130
C	-1.050051	-3.754422	4.233509
C	-1.744233	-1.540399	4.983238
C	-2.426165	-2.150225	6.060815
C	-1.725805	-4.347715	5.310728
C	6.678319	-1.518056	1.808956
C	6.991473	-4.278195	2.214816
C	5.579442	-2.379046	1.579018
C	7.914296	-2.028688	2.238394
C	8.074944	-3.410865	2.443801
C	5.755251	-3.767094	1.786042
C	-1.804971	-0.072022	4.849150
C	-2.906813	0.764710	5.016956
H	-3.946344	0.552916	5.276596
N	-0.728383	0.697039	4.472097
N	-1.103140	1.938434	4.405688
N	-2.446987	2.021963	4.715678
C	-3.071330	3.274609	4.677193
C	-4.160609	5.796741	4.608494
C	-4.467660	3.406045	4.786863
C	-2.785295	5.579640	4.489811
C	-5.018963	4.690111	4.753997
N	-2.238904	4.339063	4.517007
Ir	-0.092190	3.874601	4.460059
C	3.285085	3.714199	1.491777
C	1.055904	2.232913	2.163376
C	3.006882	2.466220	0.909098
C	1.248670	3.454975	2.839790
C	2.386898	4.200164	2.464422
N	1.916596	1.753526	1.249697
Zn	1.411157	-0.060550	0.328366

B	1.546132	2.907329	5.403515
B	-0.307866	3.998449	6.593282
B	0.656128	5.753404	4.852379
O	-0.173948	6.810563	5.289306
O	1.968187	6.204192	4.737042
C	2.077402	7.515716	5.352928
C	2.546668	7.287430	6.798105
C	3.103335	8.340772	4.574905
C	0.591378	8.046804	5.260119
C	0.148251	8.924910	6.430048
C	0.280820	8.732513	3.919720
O	-1.526448	3.610571	7.162677
O	0.487561	4.652089	7.530908
C	-1.692371	4.383022	8.386262
C	-0.195146	4.611093	8.821529
C	0.065103	5.926636	9.554507
C	0.379208	3.426813	9.607549
C	-2.542526	3.581440	9.369341
C	-2.396641	5.690172	7.982508
H	-2.022807	3.163453	1.725548
H	-3.773360	4.933988	1.968651
H	-5.241723	5.531224	0.013301
H	-4.947825	4.331621	-2.173093
H	-3.206780	2.541560	-2.401753
H	-5.103209	2.515873	4.884615
H	-6.108012	4.824767	4.836057
H	-4.548639	6.824999	4.584762
H	-2.055952	6.399746	4.415819
H	-0.056144	3.360531	10.625163
H	1.474524	3.556042	9.703277
H	0.194721	2.478151	9.066484
H	1.138727	6.006994	9.818211
H	-0.196787	6.797701	8.925239
H	-0.525794	5.975265	10.492683
H	-2.631387	6.325286	8.860652
H	-3.346717	5.437405	7.469293
H	-1.769515	6.266174	7.270295
H	-2.135207	2.563814	9.518541
H	-3.575215	3.482195	8.977106
H	-2.596093	4.093608	10.352399
H	0.196180	1.582817	2.369110
H	3.661335	2.008390	0.148503
H	2.595171	5.152318	2.973595
H	3.505604	6.732702	6.772328
H	2.704762	8.242319	7.339638
H	1.816703	6.659818	7.344207
H	3.142110	9.382273	4.956618
H	4.109733	7.891975	4.695920
H	2.872461	8.366284	3.493029
H	0.774977	9.838032	6.498403
H	0.216703	8.376855	7.388347

H	-0.906123	9.239710	6.289925
H	0.779365	9.718837	3.830608
H	-0.814164	8.890428	3.839275
H	0.597475	8.094461	3.070135
H	-0.923883	3.698421	-2.490689
H	1.393349	3.209168	-3.817345
H	5.188329	2.373061	-2.826520
H	6.282167	3.317817	-4.873867
H	5.219517	2.997235	-7.128713
H	3.058315	1.728254	-7.315378
H	1.976676	0.783701	-5.260093
H	5.475700	-0.273449	-3.118419
H	4.908211	-4.444766	1.596196
H	7.111691	-5.363222	2.364435
H	9.044295	-3.812194	2.779960
H	8.756763	-1.341720	2.418478
H	6.545386	-0.435989	1.652653
H	4.072293	-3.060979	3.770172
H	1.528677	-3.072130	4.705802
H	-2.941221	-1.509193	6.794257
H	-2.951797	-3.996266	7.080886
H	-1.714879	-5.442835	5.428382
H	-0.520283	-4.381771	3.500185
H	-3.225755	-1.319943	2.691142
H	-3.803558	0.729294	0.988632
O	1.310298	1.818952	6.244844
O	2.896362	3.038236	5.120884
C	2.584899	1.182415	6.556135
C	3.523998	1.754967	5.420219
C	2.375796	-0.331328	6.510452
C	3.000768	1.639616	7.959247
C	4.971864	2.011324	5.835823
C	3.476199	0.906294	4.138860
H	3.103594	2.742410	7.995826
H	2.220453	1.341383	8.685822
H	3.957840	1.174648	8.270913
H	3.341390	-0.869699	6.606215
H	1.716723	-0.645107	7.345387
H	1.893297	-0.635530	5.563506
H	5.036611	2.756808	6.650880
H	5.457425	1.070262	6.167435
H	5.539775	2.404387	4.968431
H	3.934079	1.477554	3.308195
H	2.435202	0.666413	3.846997
H	4.025338	-0.049184	4.253197
H	-0.209635	4.394552	2.887507
C	5.338711	-1.189915	-1.099256
H	6.286320	-1.725643	-0.970275
C	4.520357	4.497700	1.115826
H	5.102750	3.990436	0.320360
H	4.256717	5.513182	0.751524

H 5.185258 4.632622 1.995697

tBu-pyridine

Zero-point correction= 0.194981 (Hartree/Particle)
Thermal correction to Energy= 0.208587
Thermal correction to Enthalpy= 0.209705
Thermal correction to Gibbs Free Energy= 0.151726

solvent: -405.714849

C	-2.005941	-1.036042	3.245769
C	-1.634041	0.162504	3.887397
C	-1.224549	0.040264	5.239632
N	-1.169524	-1.109100	5.929326
C	-1.531057	-2.236232	5.288936
C	-1.955220	-2.250795	3.951051
H	-2.336839	-1.035968	2.196877
H	-0.919420	0.942677	5.801875
H	-1.479565	-3.173740	5.872797
H	-2.242232	-3.197003	3.465394
C	-1.655003	1.542514	3.203576
C	-2.620296	2.478275	3.976291
H	-3.650537	2.065609	3.983704
H	-2.305229	2.619375	5.030162
H	-2.652831	3.480339	3.498121
C	-0.224425	2.140551	3.220740
H	0.156705	2.273483	4.253649
H	0.486598	1.481851	2.680085
H	-0.215701	3.136265	2.728493
C	-2.129494	1.454606	1.737977
H	-1.461460	0.814691	1.124389
H	-3.162018	1.054426	1.660564
H	-2.130828	2.465981	1.281453

$\mathbf{A_{tBu,\ anticlockwise}}$

Zero-point correction= 1.412597 (Hartree/Particle)
Thermal correction to Energy= 1.538566
Thermal correction to Enthalpy= 1.539684
Thermal correction to Gibbs Free Energy= 1.242279

solvent: -5925.82645436

N	2.414349	-0.217926	-0.312520
C	2.731733	0.260176	-1.565326
C	3.572282	-0.736744	0.222875
C	4.133231	-0.009649	-1.856675
C	3.716047	-1.226151	1.549287
C	2.658923	-1.372408	2.482734
N	1.341946	-1.023106	2.272642
C	0.647918	-1.389448	3.403867

C	1.555622	-1.979992	4.376186
C	2.801930	-1.983294	3.798630
C	-0.742696	-1.229879	3.598632
C	-1.655594	-0.703057	2.649490
N	-1.330479	-0.146738	1.433183
C	-2.509070	0.280853	0.857451
C	-3.094168	-0.638130	2.853900
C	-3.626063	-0.026838	1.740833
C	-2.632681	0.892063	-0.417250
C	-1.566783	1.074353	-1.339151
N	-0.267399	0.645559	-1.162367
C	0.453079	1.105236	-2.245152
C	-1.675296	1.827771	-2.580795
C	-0.418642	1.851932	-3.140113
C	1.846959	0.928758	-2.449175
C	2.697664	1.648729	-6.110827
C	3.357516	2.612428	-3.557689
C	2.124517	1.062794	-4.969452
C	3.602151	2.717821	-5.979045
C	3.930285	3.198431	-4.698351
C	2.447669	1.536260	-3.677841
C	-5.883983	2.900882	-0.459597
C	-4.661934	0.844461	-1.931807
C	-6.547612	2.346741	-1.569002
C	-4.614706	2.426993	-0.089914
C	-3.984175	1.391256	-0.817416
C	-5.931155	1.317803	-2.303825
C	-1.274300	-1.394605	4.991178
C	-2.334665	-1.493235	7.609734
C	-1.577992	-2.631172	5.588457
C	-1.494893	-0.195274	5.727296
C	-2.027458	-0.250162	7.027780
C	-2.104854	-2.680442	6.892646
C	5.672851	-0.766106	3.048458
C	7.149763	-2.879592	1.936421
C	5.095449	-1.553088	2.025624
C	6.974915	-1.027914	3.503530
C	7.719345	-2.083425	2.946950
C	5.847165	-2.617491	1.479014
C	-1.115814	1.070229	5.055880
C	-1.909258	2.019654	4.425010
H	-2.993682	2.117283	4.342422
N	0.202867	1.353051	4.784869
N	0.263428	2.414954	4.045824
N	-1.022291	2.845554	3.784665
C	-1.221607	4.018639	3.033346
C	-1.494840	6.381942	1.670363
C	-2.499607	4.374139	2.567368
C	-0.258337	5.938962	2.157703
C	-2.633012	5.579111	1.865888
N	-0.112434	4.767305	2.821775

Ir	1.776877	3.991580	3.823308
C	1.349562	4.360845	0.089557
C	2.418811	2.408842	1.728585
C	0.694508	3.138745	0.321110
C	2.611678	4.560385	0.648492
C	3.225465	3.554629	1.452425
N	1.215078	2.191548	1.114388
Zn	0.605222	0.144766	0.711386
B	3.051533	3.088002	5.138605
B	1.076715	4.681873	5.597495
B	2.865559	5.724262	3.838139
O	2.300598	6.932030	3.372450
O	4.170184	5.967787	4.284325
C	4.351841	7.395415	4.465669
C	4.067664	7.683683	5.948919
C	5.796304	7.762783	4.119099
C	3.256566	8.013358	3.494050
C	2.526255	9.235958	4.058915
C	3.782597	8.321035	2.082892
O	1.126020	5.982782	6.099654
O	0.283015	3.865282	6.420173
C	0.546661	5.974644	7.435702
C	-0.409849	4.718005	7.363075
C	-0.580281	3.954501	8.675868
C	-1.778287	5.041213	6.737202
C	-0.158607	7.310734	7.667123
C	1.702309	5.782995	8.427695
H	-4.081600	2.865526	0.766700
H	-6.356831	3.711935	0.117624
H	-7.543482	2.716233	-1.860795
H	-6.446179	0.874799	-3.171197
H	-4.180852	0.033866	-2.501197
H	-3.360083	3.715046	2.744938
H	-3.616670	5.883720	1.477416
H	-1.557513	7.343963	1.141220
H	0.666676	6.529861	2.073099
H	-1.648902	5.596830	5.785528
H	-2.423021	5.637150	7.414595
H	-2.293504	4.086044	6.507462
H	-1.257526	3.089819	8.519907
H	-1.019379	4.603522	9.461679
H	0.389288	3.561265	9.035221
H	1.350861	5.777068	9.479307
H	2.425382	6.613258	8.305816
H	2.240461	4.843017	8.203668
H	0.590749	8.127788	7.693560
H	-0.877167	7.538269	6.856993
H	-0.698981	7.307702	8.636629
H	2.808975	1.555302	2.306904
H	-0.270852	2.905487	-0.152910
H	0.876677	5.125258	-0.543453

H	3.152708	5.493055	0.439872
H	3.027117	7.397582	6.194159
H	4.237749	8.748278	6.209503
H	4.740329	7.053253	6.563904
H	5.950693	8.859672	4.188457
H	6.484732	7.271450	4.835955
H	6.077292	7.430840	3.101770
H	3.236335	10.061181	4.274619
H	1.981322	8.976204	4.985405
H	1.784937	9.604755	3.320362
H	4.474478	9.187577	2.075091
H	4.309574	7.446944	1.654923
H	2.922572	8.557000	1.422946
H	-2.588748	2.297085	-2.963623
H	-0.107506	2.341825	-4.069714
H	3.602689	2.988977	-2.551769
H	4.634776	4.038209	-4.586703
H	4.051898	3.175785	-6.874379
H	2.440489	1.263375	-7.110492
H	1.421070	0.221115	-5.068070
H	4.646729	0.239824	-2.792082
H	5.395789	-3.244544	0.694060
H	7.722241	-3.716136	1.504564
H	8.741442	-2.289061	3.302890
H	7.410810	-0.400300	4.297361
H	5.081843	0.053072	3.485901
H	3.735439	-2.384151	4.208912
H	1.266931	-2.358619	5.363410
H	-2.180699	0.686265	7.586872
H	-2.747104	-1.534741	8.630082
H	-2.342025	-3.654234	7.349859
H	-1.407407	-3.556454	5.016399
H	-3.625582	-1.031527	3.728967
H	-4.682107	0.163498	1.516349
O	3.521201	3.632755	6.347685
O	3.359402	1.713880	5.085321
C	4.272925	2.614059	7.057717
C	3.693910	1.286433	6.435491
C	5.758205	2.838436	6.730611
C	4.033078	2.766143	8.560582
C	4.686954	0.128198	6.374404
C	2.382881	0.835044	7.100895
H	2.953403	2.752620	8.800825
H	4.452559	3.729071	8.916253
H	4.527724	1.945106	9.120356
H	6.020148	3.888029	6.971687
H	5.950678	2.682471	5.651432
H	6.418344	2.163950	7.313595
H	5.588178	0.390957	5.790627
H	4.209219	-0.744813	5.889264
H	5.004133	-0.168157	7.395978

H	2.557056	0.415080	8.113034
H	1.666402	1.679125	7.164859
H	1.914624	0.063417	6.459608
C	4.656742	-0.625161	-0.745621
H	5.689732	-0.952435	-0.585839
C	4.766521	3.508131	1.598189
C	5.230021	2.490423	0.519962
H	6.338751	2.461108	0.471444
H	4.874170	1.469985	0.758049
H	4.844497	2.748063	-0.488742
C	5.419939	4.874719	1.314153
H	5.055939	5.629706	2.036421
H	6.517628	4.788305	1.446447
H	5.244479	5.232585	0.278357
C	5.255167	3.022968	2.972521
H	6.352084	2.855521	2.936471
H	5.032733	3.778326	3.749842
H	4.777615	2.074386	3.272737

A$_{tBu, clockwise}$

Zero-point correction= 1.413724 (Hartree/Particle)
Thermal correction to Energy= 1.539295
Thermal correction to Enthalpy= 1.540413
Thermal correction to Gibbs Free Energy= 1.245439

solvent: -5925.83859472

N	2.168545	0.127354	-0.636962
C	2.398343	0.603450	-1.908524
C	3.395907	-0.185914	-0.094054
C	3.826070	0.551215	-2.203274
C	3.619238	-0.707733	1.209401
C	2.604203	-1.076163	2.131234
N	1.249951	-0.920825	1.936597
C	0.623606	-1.377174	3.076342
C	1.617151	-1.843395	4.033005
C	2.846563	-1.668840	3.440982
C	-0.774268	-1.394635	3.286190
C	-1.750596	-0.926631	2.369634
N	-1.501010	-0.185819	1.234521
C	-2.726434	0.184476	0.718924
C	-3.183353	-1.058780	2.569171
C	-3.792102	-0.367808	1.543133
C	-2.926421	0.968465	-0.450002
C	-1.895560	1.333612	-1.356727
N	-0.570387	0.950105	-1.260490
C	0.046425	1.345840	-2.430377
C	-2.100721	2.079611	-2.590066
C	-0.902023	2.068202	-3.265387
C	1.408579	1.130128	-2.780062

C	1.534042	1.017703	-6.589232
C	2.723899	2.478168	-4.509145
C	1.205057	0.736461	-5.253466
C	2.460841	2.031657	-6.891044
C	3.053648	2.760427	-5.845404
C	1.795411	1.461492	-4.190248
C	-6.402009	2.563816	-0.230493
C	-4.946339	0.913160	-1.972527
C	-6.996790	2.109794	-1.420664
C	-5.083717	2.194659	0.084807
C	-4.329586	1.369086	-0.782548
C	-6.264169	1.280454	-2.288927
C	-1.261693	-1.693420	4.674052
C	-2.105007	-2.097062	7.340353
C	-1.491639	-2.994112	5.158208
C	-1.462671	-0.580047	5.542099
C	-1.872395	-0.792090	6.871521
C	-1.916991	-3.195772	6.484099
C	5.561247	-0.024206	2.671202
C	7.240597	-1.913764	1.431268
C	5.041202	-0.858677	1.653713
C	6.913767	-0.122222	3.042084
C	7.757068	-1.062473	2.424137
C	5.893299	-1.809932	1.047534
C	-1.132819	0.766291	5.008197
C	-1.789085	1.511089	4.031991
H	-2.752642	1.369424	3.535317
N	0.084352	1.357582	5.265342
N	0.206316	2.400460	4.496576
N	-0.921504	2.521374	3.720298
C	-0.972841	3.540608	2.745012
C	-0.963264	5.600575	0.938298
C	-1.930676	3.526161	1.721146
C	-0.014473	5.507211	1.967377
C	-1.920303	4.578323	0.796865
N	-0.005017	4.482471	2.849835
Ir	1.662601	3.969928	4.253727
C	2.399359	4.074356	0.266359
C	2.213668	2.148375	2.292377
C	1.382425	3.095868	0.329489
C	3.338578	4.062896	1.310950
C	3.254343	3.106394	2.348302
N	1.296647	2.152133	1.284393
Zn	0.392345	0.311840	0.502083
B	3.004433	3.182982	5.562411
B	0.915251	4.949386	5.859918
B	2.878295	5.621415	4.149485
O	2.418085	6.897730	3.781976
O	4.246838	5.681351	4.464702
C	4.605334	7.064312	4.715645
C	4.518221	7.276483	6.236779

C	6.040808	7.295505	4.235095
C	3.493240	7.864982	3.905886
C	2.938753	9.092824	4.634497
C	3.938844	8.241984	2.487042
O	1.445466	6.042625	6.545222
O	-0.327007	4.580384	6.399874
C	0.673664	6.232154	7.759102
C	-0.720005	5.604122	7.354311
C	-1.482625	4.943007	8.502246
C	-1.621990	6.594700	6.599295
C	0.638836	7.726465	8.083276
C	1.383658	5.449583	8.875337
H	-4.628860	2.553634	1.020878
H	-6.966381	3.212807	0.457940
H	-8.030547	2.397793	-1.668852
H	-6.725711	0.908563	-3.217583
H	-4.378019	0.252204	-2.645178
H	-2.623868	2.681797	1.621650
H	-2.644609	4.579658	-0.031855
H	-0.930487	6.452577	0.243899
H	0.783653	6.251291	2.125289
H	-1.061264	7.078549	5.774022
H	-2.029404	7.381269	7.266811
H	-2.471492	6.037064	6.155622
H	-2.444400	4.534337	8.130371
H	-1.706282	5.679107	9.302464
H	-0.902940	4.107469	8.937554
H	0.890949	5.592808	9.858700
H	2.430293	5.807398	8.945649
H	1.429175	4.371971	8.626463
H	1.658599	8.074091	8.346701
H	0.292592	8.321440	7.216965
H	-0.027762	7.929044	8.947333
H	2.174848	1.308018	2.999505
H	0.609211	3.029392	-0.449131
H	4.168543	4.785819	1.334893
H	3.497374	7.043138	6.595631
H	4.798673	8.308636	6.530123
H	5.215510	6.570809	6.729620
H	6.329989	8.361399	4.344824
H	6.736085	6.686082	4.847203
H	6.175694	6.998435	3.177570
H	3.741318	9.827772	4.852524
H	2.451712	8.794812	5.581745
H	2.176475	9.591067	4.000821
H	4.707615	9.041020	2.488854
H	4.353294	7.358813	1.962020
H	3.060926	8.600449	1.912555
H	-3.041957	2.542479	-2.907380
H	-0.673932	2.521932	-4.236461
H	3.181512	3.060936	-3.699056

H	3.775963	3.561630	-6.069416
H	2.719536	2.253289	-7.938602
H	1.066062	0.437021	-7.400175
H	0.482212	-0.059770	-5.016079
H	4.294191	0.831807	-3.153192
H	5.484686	-2.471242	0.267222
H	7.889799	-2.663819	0.951794
H	8.815911	-1.134262	2.719636
H	7.310857	0.546945	3.820357
H	4.904302	0.704766	3.174269
H	3.833103	-1.920632	3.846348
H	1.395296	-2.253183	5.025125
H	-2.003083	0.075222	7.537041
H	-2.429787	-2.256078	8.380635
H	-2.096551	-4.218683	6.851411
H	-1.328007	-3.849910	4.484936
H	-3.657548	-1.617965	3.384622
H	-4.864990	-0.267804	1.342012
O	3.070090	3.425631	6.938981
O	3.911459	2.152680	5.211926
C	4.188164	2.685480	7.486243
C	4.331373	1.507897	6.450456
C	5.398014	3.634934	7.482340
C	3.842764	2.249059	8.910200
C	5.751854	0.969925	6.293517
C	3.341912	0.360350	6.708555
H	2.873543	1.716248	8.940778
H	3.762286	3.139617	9.566264
H	4.630484	1.584910	9.323490
H	5.135915	4.538636	8.068262
H	5.627864	3.961448	6.448120
H	6.298044	3.170819	7.934959
H	6.430412	1.742068	5.882405
H	5.756627	0.100880	5.606323
H	6.150240	0.631401	7.272762
H	3.623944	-0.239490	7.598169
H	2.310629	0.747363	6.831880
H	3.334153	-0.301918	5.820895
H	4.054192	3.008499	3.094508
C	4.448001	0.100459	-1.063711
H	5.522001	-0.039238	-0.898967
C	2.552346	5.012601	-0.943260
C	3.893609	4.646289	-1.633347
H	4.762466	4.880802	-0.984598
H	3.935082	3.560117	-1.859086
H	4.015648	5.215105	-2.579548
C	1.399395	4.846320	-1.955073
H	1.340376	3.819446	-2.364686
H	0.416991	5.080982	-1.496423
H	1.546976	5.537236	-2.810225
C	2.598086	6.487635	-0.486287

H	2.784072	7.154635	-1.354135
H	1.640892	6.793898	-0.019091
H	3.399959	6.666972	0.254046

$TS_{AB,\ tBu}$

Zero-point correction= 1.408657 (Hartree/Particle)
Thermal correction to Energy= 1.533820
Thermal correction to Enthalpy= 1.534938
Thermal correction to Gibbs Free Energy= 1.241172

solvent: -5925.79053822

N	3.131439	-0.471284	-0.644108
C	3.572226	0.027317	-1.851997
C	4.198735	-1.096391	-0.040849
C	4.959177	-0.372515	-2.059247
C	4.189371	-1.710855	1.241930
C	3.035916	-1.947633	2.036715
N	1.748135	-1.598392	1.694854
C	0.924037	-2.032040	2.708793
C	1.721991	-2.676816	3.743418
C	3.034119	-2.612196	3.333162
C	-0.482612	-1.834870	2.770517
C	-1.230099	-1.046901	1.855769
N	-0.681576	-0.260533	0.866868
C	-1.668641	0.585888	0.426608
C	-2.657669	-0.778477	1.950747
C	-2.922155	0.267566	1.095035
C	-1.500315	1.646826	-0.507233
C	-0.406491	1.773271	-1.404970
N	0.671037	0.914154	-1.454639
C	1.516890	1.381215	-2.430583
C	-0.240704	2.826919	-2.398024
C	0.937324	2.565107	-3.058748
C	2.833581	0.901991	-2.697279
C	3.593242	1.813650	-6.325993
C	4.706776	2.226243	-3.782033
C	2.970769	1.270812	-5.189970
C	4.775378	2.564372	-6.194449
C	5.328592	2.770112	-4.917676
C	3.519825	1.464288	-3.900164
C	-3.598915	4.431019	0.998521
C	-3.332902	3.142546	-1.488162
C	-4.409884	4.821895	-0.083278
C	-2.660191	3.400696	0.837137
C	-2.512581	2.740019	-0.410524
C	-4.273211	4.175072	-1.325036
C	-1.212634	-2.418936	3.938610
C	-2.632273	-3.619652	6.092917
C	-1.247347	-3.822139	4.105870

C	-1.899905	-1.608374	4.893723
C	-2.604554	-2.224513	5.953148
C	-1.944749	-4.421831	5.165789
C	6.462643	-1.116014	2.139845
C	7.091717	-3.815074	2.588902
C	5.513910	-2.112087	1.809247
C	7.707101	-1.464265	2.690258
C	8.025373	-2.815532	2.917232
C	5.846228	-3.466357	2.039692
C	-1.919467	-0.135520	4.795267
C	-3.001038	0.724066	4.975593
H	-4.049178	0.530913	5.214327
N	-0.817201	0.616845	4.458230
N	-1.158171	1.869465	4.426624
N	-2.504363	1.978308	4.723512
C	-3.102549	3.244090	4.705160
C	-4.141106	5.788347	4.665288
C	-4.496273	3.402482	4.816462
C	-2.770321	5.544919	4.547475
C	-5.021741	4.697484	4.796573
N	-2.249353	4.293111	4.558641
Ir	-0.113563	3.790122	4.468261
C	3.178469	3.635350	1.414269
C	0.969909	2.130109	2.146594
C	2.880704	2.393232	0.829374
C	1.176862	3.349923	2.817630
C	2.302332	4.098435	2.416362
N	1.807101	1.671476	1.202516
Zn	1.307889	-0.130245	0.269399
B	1.578637	2.847819	5.332697
B	-0.274015	3.913880	6.606744
B	0.670575	5.659085	4.834998
O	-0.119150	6.727675	5.317059
O	1.981499	6.093882	4.653725
C	2.142970	7.396172	5.274227
C	2.671354	7.143937	6.694905
C	3.148837	8.205786	4.455172
C	0.664051	7.952333	5.252314
C	0.291584	8.836177	6.442026
C	0.303019	8.643642	3.927650
O	-1.494276	3.570646	7.200210
O	0.565513	4.532856	7.529746
C	-1.610351	4.351579	8.424586
C	-0.097846	4.531700	8.831620
C	0.221083	5.847729	9.540522
C	0.447251	3.340749	9.628564
C	-2.467901	3.581524	9.426479
C	-2.280577	5.679181	8.029347
H	-2.011135	3.106663	1.678701
H	-3.691643	4.933955	1.974077
H	-5.148222	5.630310	0.040622

H	-4.911307	4.470489	-2.173422
H	-3.240715	2.622270	-2.454383
H	-5.150192	2.524809	4.905230
H	-6.108008	4.852901	4.878975
H	-4.508393	6.824406	4.652852
H	-2.024595	6.351161	4.483975
H	0.019024	3.303096	10.650637
H	1.546655	3.436479	9.714628
H	0.227619	2.389546	9.105163
H	1.301830	5.894514	9.782409
H	-0.022845	6.717945	8.903050
H	-0.349487	5.930342	10.488830
H	-2.475846	6.325321	8.909126
H	-3.249439	5.454486	7.538567
H	-1.651790	6.231503	7.300193
H	-2.091589	2.551690	9.573313
H	-3.510101	3.514337	9.053147
H	-2.487217	4.099101	10.407975
H	0.126121	1.467229	2.377062
H	3.498314	1.932926	0.045440
H	2.514168	5.037313	2.943288
H	3.616095	6.569653	6.620536
H	2.872600	8.089464	7.238511
H	1.953476	6.524976	7.267168
H	3.239760	9.239184	4.849555
H	4.145944	7.724746	4.513159
H	2.861146	8.256283	3.387808
H	0.936295	9.738471	6.480371
H	0.396884	8.286220	7.395893
H	-0.762850	9.168717	6.352777
H	0.812137	9.622309	3.816419
H	-0.791873	8.818746	3.897248
H	0.571341	8.001575	3.064494
H	-0.926288	3.666050	-2.562409
H	1.398454	3.148928	-3.863774
H	5.129240	2.401722	-2.781014
H	6.247547	3.367096	-4.802923
H	5.263046	2.991272	-7.085315
H	3.153580	1.645739	-7.322267
H	2.045924	0.681319	-5.290253
H	5.553848	-0.170012	-2.957022
H	5.115221	-4.246435	1.775203
H	7.337220	-4.876083	2.756519
H	9.001429	-3.089851	3.348242
H	8.431381	-0.674842	2.947735
H	6.202926	-0.059548	1.967625
H	3.920398	-2.969308	3.869488
H	1.334824	-3.096962	4.678601
H	-3.110367	-1.587325	6.696331
H	-3.179939	-4.078451	6.930976
H	-1.956415	-5.518905	5.263449

H	-0.721270	-4.445373	3.366285
H	-3.363851	-1.313296	2.596242
H	-3.884376	0.755290	0.898772
O	1.414598	1.704242	6.112897
O	2.919920	3.088235	5.068701
C	2.735638	1.184964	6.452137
C	3.639818	1.848600	5.341725
C	2.678231	-0.340528	6.404157
C	3.072353	1.679740	7.864059
C	5.060812	2.200773	5.779053
C	3.667896	1.026368	4.041455
H	3.073643	2.787422	7.897713
H	2.299875	1.314514	8.568101
H	4.057559	1.301900	8.204631
H	3.690315	-0.777154	6.534850
H	2.028301	-0.718755	7.219296
H	2.261972	-0.696244	5.444196
H	5.061464	2.932809	6.608764
H	5.609187	1.290701	6.099059
H	5.608041	2.651203	4.926675
H	4.086912	1.650672	3.229312
H	2.651614	0.714035	3.731748
H	4.290242	0.114584	4.140582
H	-0.273549	4.318677	2.904847
C	5.353964	-1.047100	-0.925882
H	6.331942	-1.496199	-0.716530
C	4.449405	4.427682	1.066508
C	4.115823	5.931674	0.910968
H	3.722926	6.366427	1.852357
H	5.028488	6.501692	0.636127
H	3.356309	6.089269	0.117032
C	5.092158	3.927970	-0.245924
H	4.381024	3.999816	-1.095216
H	5.983462	4.542022	-0.491915
H	5.429012	2.872941	-0.171885
C	5.451928	4.242358	2.235438
H	4.999633	4.553304	3.199794
H	5.746757	3.176272	2.332987
H	6.373103	4.840064	2.064215

Ir with L* as ligand

Zero-point correction= 0.909703 (Hartree/Particle)
Thermal correction to Energy= 0.986136
Thermal correction to Enthalpy= 0.987255
Thermal correction to Gibbs Free Energy= 0.792202

solvent: -2371.740676

C	-0.848488	-2.309886	4.903477
C	-3.214709	-3.824639	4.787526

C	-0.793275	-3.709216	4.982661
C	-2.093251	-1.649791	4.765074
C	-3.274986	-2.425478	4.707273
C	-1.973476	-4.472774	4.925570
C	-2.134499	-0.182494	4.685773
C	-3.222346	0.688528	4.607779
H	-4.300711	0.517691	4.577650
N	-0.980372	0.571433	4.677226
N	-1.299316	1.830053	4.602161
N	-2.669165	1.941978	4.546863
C	-3.247632	3.229721	4.553292
C	-4.205284	5.788908	4.716369
C	-4.622807	3.410981	4.782713
C	-2.854510	5.511770	4.485530
C	-5.112142	4.720509	4.850819
N	-2.371626	4.248353	4.376009
Ir	-0.186892	3.672593	4.567003
B	1.586447	2.931579	5.233087
B	-0.578471	3.946373	6.559540
B	0.695310	5.515595	4.802277
O	-0.041920	6.718689	4.882520
O	2.063747	5.804161	4.914566
C	2.224344	7.165217	5.393208
C	2.373442	7.090876	6.922160
C	3.484421	7.761564	4.762442
C	0.868244	7.847079	4.938131
C	0.304014	8.870598	5.926221
C	0.938817	8.449592	3.525963
O	-1.512990	3.114553	7.205430
O	-0.132130	4.939996	7.431861
C	-1.849094	3.723476	8.480281
C	-0.566886	4.597312	8.774125
C	-0.831647	5.890014	9.546896
C	0.571757	3.794138	9.423038
C	-2.123311	2.620475	9.501672
C	-3.108562	4.574379	8.241735
H	-5.287740	2.548737	4.929236
H	-6.182058	4.902450	5.032752
H	-4.537948	6.834348	4.793018
H	-2.091231	6.303543	4.414277
H	0.348035	3.532162	10.477308
H	1.492975	4.410541	9.399798
H	0.778570	2.877187	8.837572
H	0.122065	6.433534	9.705151
H	-1.514358	6.561114	8.991629
H	-1.272813	5.672420	10.541954
H	-3.494428	5.028549	9.177118
H	-3.899692	3.926926	7.811792
H	-2.900339	5.380948	7.509626
H	-1.285972	1.898882	9.543248
H	-3.039413	2.062690	9.218585

H	-2.278589	3.049038	10.513861
H	3.251897	6.460162	7.162041
H	2.529504	8.091912	7.373477
H	1.483508	6.605773	7.367952
H	3.595890	8.831598	5.035906
H	4.376566	7.217076	5.132738
H	3.471069	7.674684	3.659408
H	1.018551	9.704339	6.088032
H	0.078007	8.396545	6.899767
H	-0.638438	9.298834	5.526718
H	1.560924	9.367011	3.495649
H	-0.085692	8.710351	3.191588
H	1.353345	7.716468	2.806464
H	-4.254089	-1.933123	4.595410
H	-4.143848	-4.414414	4.740673
H	-1.927245	-5.571446	4.988350
H	0.182212	-4.209172	5.091335
O	1.883087	2.628412	6.571124
O	2.653973	2.536565	4.405404
C	3.288645	2.280093	6.653664
C	3.565850	1.734270	5.200452
C	3.483074	1.261462	7.776607
C	4.051460	3.581497	6.952356
C	4.991389	1.948826	4.692617
C	3.136749	0.266508	5.026292
H	3.891042	4.312384	6.134238
H	3.638669	4.022951	7.881688
H	5.137860	3.408490	7.094465
H	4.527690	0.886105	7.793339
H	3.272586	1.738278	8.755682
H	2.795392	0.401834	7.663932
H	5.241945	3.025446	4.646862
H	5.725281	1.437192	5.350066
H	5.094561	1.532025	3.669989
H	3.151016	0.017959	3.945784
H	2.100119	0.121402	5.393772
H	3.814679	-0.433526	5.556267
C	-2.143255	5.162527	1.387197
C	-2.419996	3.949829	0.864521
C	-1.449542	2.911110	0.351593
C	-0.789319	2.047902	1.447326
C	0.315796	2.658506	2.287705
C	0.866930	3.932292	2.274613
C	0.506565	5.134425	1.408095
C	-0.833529	5.855352	1.641556
H	-3.015146	5.761095	1.711833
H	-3.486814	3.659604	0.832261
H	0.936315	1.898345	2.793309
H	1.870707	3.993677	2.725916
H	-1.999609	2.225566	-0.327529
H	-0.658555	3.373613	-0.272650

H	-1.578602	1.615920	2.101969
H	-0.328824	1.160340	0.954923
H	1.297922	5.892702	1.583228
H	0.609330	4.855352	0.336504
H	-0.835731	6.201653	2.697442
H	-0.825462	6.798351	1.043872
H	0.070045	-1.706094	4.950172

ZnTPP

Zero-point correction= 0.579885 (Hartree/Particle)
Thermal correction to Energy= 0.633026
Thermal correction to Enthalpy= 0.634145
Thermal correction to Gibbs Free Energy= 0.485462

solvent: -3692.88346457

Zn	-0.203664	6.445246	3.907549
N	-0.268903	8.507298	3.826014
N	0.953674	6.414547	2.198672
N	-0.119653	4.384997	4.001984
N	-1.382075	6.474703	5.602286
C	0.266522	9.307539	2.833536
C	1.050680	8.862851	1.739168
C	1.366817	7.509129	1.461779
C	2.185379	7.067312	0.344415
H	2.658414	7.725257	-0.393007
C	2.239139	5.695231	0.406464
H	2.751974	5.010924	-0.278588
C	1.472364	5.294918	1.574723
C	1.277464	3.951862	1.982954
C	0.526325	3.544917	3.113688
C	0.343184	2.162534	3.524018
H	0.756154	1.289151	3.007074
C	-0.430234	2.182278	4.660155
H	-0.786139	1.326986	5.245182
C	-0.703807	3.578096	4.960934
C	-1.458545	4.026313	6.074001
C	-1.744497	5.383122	6.369954
C	-2.443429	5.832284	7.562612
H	-2.818554	5.180064	8.359160
C	-2.505161	7.203900	7.496731
H	-2.939245	7.892651	8.230029
C	-1.844613	7.597860	6.263207
C	-1.686308	8.939474	5.831986
C	-0.969954	9.342409	4.676419
C	-0.889041	10.711933	4.195844
H	-1.377321	11.575642	4.660742
C	-0.122985	10.690350	3.054720
H	0.138991	11.532925	2.405072
C	1.573191	9.904548	0.798443

C	2.532554	10.848736	1.230686
H	2.896077	10.802282	2.269297
C	3.021385	11.828543	0.350633
H	3.772609	12.553374	0.702884
C	2.558667	11.880894	-0.976411
H	2.941843	12.649239	-1.666539
C	1.603559	10.947510	-1.417502
H	1.231446	10.985422	-2.453788
C	1.114687	9.967755	-0.537438
H	0.361821	9.239645	-0.878058
C	1.915083	2.878290	1.154209
C	1.123674	2.002247	0.377165
H	0.028931	2.122768	0.385757
C	1.721219	0.995213	-0.399180
H	1.089793	0.323266	-1.002170
C	3.119892	0.848918	-0.412016
H	3.588430	0.059674	-1.021131
C	3.917267	1.715474	0.356823
H	5.013411	1.604663	0.355691
C	3.319589	2.721647	1.134228
H	3.940697	3.398884	1.741382
C	-1.985047	2.990099	7.018559
C	-1.103276	2.212835	7.804137
H	-0.018739	2.382495	7.714844
C	-1.599256	1.243666	8.691923
H	-0.898027	0.650432	9.300310
C	-2.985251	1.035837	8.809456
H	-3.374066	0.275932	9.505747
C	-3.872170	1.803829	8.033936
H	-4.959121	1.644338	8.116997
C	-3.376188	2.772997	7.146112
H	-4.067832	3.373183	6.534232
C	-2.323982	10.011571	6.659433
C	-3.730084	10.080084	6.792995
H	-4.352027	9.334999	6.272463
C	-4.328682	11.085955	7.569533
H	-5.426073	11.127566	7.657690
C	-3.531532	12.040474	8.226389
H	-4.000861	12.829181	8.835573
C	-2.131887	11.982641	8.101318
H	-1.500003	12.723135	8.617244
C	-1.533254	10.977102	7.324367
H	-0.437231	10.925594	7.229773

ZS

Zero-point correction= 0.719259 (Hartree/Particle)
Thermal correction to Energy= 0.778631
Thermal correction to Enthalpy= 0.779749
Thermal correction to Gibbs Free Energy= 0.619092

solvent: -3439.775554

Zn	2.737969	5.846667	1.664297
O	2.659446	4.111270	2.501718
N	3.228961	7.723834	0.991997
N	2.809574	6.802882	3.480177
C	3.385765	8.002712	-0.290704
H	3.702386	9.028958	-0.578460
C	3.368156	8.657894	2.031716
C	3.706625	10.020463	1.856046
H	3.890991	10.412053	0.844583
C	3.807855	10.887645	2.949739
C	3.570267	10.408554	4.253700
H	3.649228	11.087596	5.116854
C	3.233959	9.065200	4.455502
H	3.055654	8.707019	5.480350
C	3.125319	8.166920	3.367711
C	2.586570	6.178030	4.623617
H	2.593331	6.769883	5.564827
C	2.329678	4.779559	4.806604
C	2.040124	4.353648	6.139754
H	2.032826	5.125493	6.928552
C	1.766531	3.028014	6.457526
C	1.783718	2.092974	5.380164
H	1.555260	1.045452	5.609724
C	2.068850	2.417915	4.051024
C	2.367893	3.796972	3.729391
C	3.188622	7.112847	-1.397224
C	2.682748	5.751064	-1.266597
C	3.498384	7.628784	-2.693535
C	2.549373	4.953228	-2.466736
C	3.358505	6.872179	-3.851608
H	3.866927	8.667471	-2.751189
C	2.887777	5.534947	-3.691711
H	2.785698	4.918528	-4.592703
O	2.348743	5.232399	-0.121897
H	4.072083	11.944045	2.786735
C	1.454849	2.617411	7.913987
C	2.659701	2.981680	8.818494
C	0.193782	3.375249	8.401856
C	1.190557	1.102614	8.050157
H	3.577657	2.454859	8.484345
H	2.872373	4.070480	8.802626
H	2.459825	2.694737	9.873258
H	-0.682399	3.134808	7.764396
H	-0.052273	3.095802	9.448872
H	0.338137	4.474953	8.373714
H	0.975803	0.852001	9.109766
H	0.315709	0.778389	7.449043
H	2.067962	0.499156	7.737264
C	3.710595	7.473812	-5.230418

C	2.829538	8.723456	-5.484747
C	5.205569	7.883066	-5.244635
C	3.472732	6.474012	-6.382242
H	1.752432	8.455973	-5.473080
H	2.990241	9.504837	-4.713658
H	3.063946	9.174740	-6.472787
H	5.857090	7.004141	-5.057906
H	5.484613	8.318644	-6.228139
H	5.432005	8.640560	-4.466242
H	3.732466	6.948113	-7.351497
H	4.101082	5.564960	-6.279146
H	2.411118	6.155715	-6.441952
C	2.055321	3.492536	-2.359733
C	3.003388	2.680353	-1.434980
C	0.613223	3.469264	-1.785850
C	2.027528	2.793524	-3.736247
H	4.036265	2.672424	-1.842880
H	3.028505	3.102121	-0.413443
H	2.655379	1.627721	-1.364016
H	-0.083928	4.015249	-2.456008
H	0.255113	2.421335	-1.696448
H	0.576726	3.934239	-0.783482
H	1.672354	1.749545	-3.612424
H	1.337231	3.294046	-4.447828
H	3.034526	2.747242	-4.202326
C	2.059457	1.357872	2.926160
C	1.026898	1.754361	1.835140
C	3.474572	1.250544	2.297260
C	1.670935	-0.039110	3.456872
H	0.005542	1.816444	2.266706
H	1.278448	2.728637	1.377318
H	1.012168	0.989395	1.029667
H	4.218288	0.932310	3.058081
H	3.473060	0.496940	1.480890
H	3.795540	2.219721	1.873091
H	1.672175	-0.765566	2.618041
H	2.387715	-0.413650	4.218009
H	0.653509	-0.049388	3.902005

$\mathbf{TS_{AB,\ ZnTPP}}$

Zero-point correction= 1.398577 (Hartree/Particle)
Thermal correction to Energy= 1.524985
Thermal correction to Enthalpy= 1.526103
Thermal correction to Gibbs Free Energy= 1.224172

solvent: -6000.82819426

N	3.628650	-0.734675	0.117308
C	3.845121	-0.116083	-1.095107
C	4.816681	-1.317885	0.494255

C	5.212820	-0.365909	-1.532341
C	5.033690	-2.053741	1.692533
C	4.010345	-2.466131	2.588569
N	2.667543	-2.173801	2.458889
C	2.004036	-2.875461	3.440750
C	2.966027	-3.609712	4.251316
C	4.210601	-3.336209	3.737513
C	0.598805	-2.872825	3.668500
C	-0.323519	-1.996019	3.032091
N	0.007543	-1.031449	2.102326
C	-1.150844	-0.347551	1.793444
C	-1.753346	-1.942517	3.295867
C	-2.256165	-0.889435	2.564225
C	-1.252424	0.699720	0.835882
C	-0.269605	1.007673	-0.141421
N	0.957713	0.399020	-0.266496
C	1.575662	0.956650	-1.360846
C	-0.448681	1.982750	-1.211275
C	0.693020	1.943446	-1.975199
C	2.903239	0.686615	-1.793028
C	3.166593	1.674308	-5.474338
C	4.396479	2.313321	-3.032387
C	2.747883	1.044316	-4.290726
C	4.201963	2.625656	-5.441092
C	4.814878	2.943743	-4.215741
C	3.358432	1.352897	-3.052943
C	-3.367500	3.816613	1.332539
C	-3.757110	1.141530	0.563403
C	-4.661345	3.358877	1.028743
C	-2.275686	2.936877	1.266596
C	-2.454769	1.587505	0.882141
C	-4.852730	2.018688	0.643473
C	0.075259	-3.884315	4.639723
C	-0.869851	-5.853943	6.462664
C	0.293319	-5.263884	4.402833
C	-0.629150	-3.520720	5.811181
C	-1.096724	-4.489692	6.714020
C	-0.172819	-6.235960	5.302055
C	7.412997	-1.415863	2.203880
C	8.180254	-4.101229	2.459157
C	6.444374	-2.429703	2.011022
C	8.740402	-1.737063	2.529952
C	9.127707	-3.082434	2.665937
C	6.853086	-3.778360	2.130830
C	2.503683	0.380526	5.838518
C	1.200446	-0.095874	5.686377
H	0.808369	-1.105990	5.542341
N	2.466275	1.756648	5.830416
N	1.230927	2.136075	5.679476
N	0.428226	1.027210	5.584367
C	-0.950508	1.196586	5.318538

C	-3.577564	1.674650	4.734220
C	-1.898503	0.250515	5.727992
C	-2.563015	2.581498	4.401590
C	-3.241560	0.493102	5.415072
N	-1.259702	2.344367	4.674655
Ir	0.366920	3.970602	4.885165
C	3.939750	3.105236	2.369636
C	1.445545	2.028868	2.730943
C	3.648080	1.782727	2.015490
C	1.664897	3.334458	3.220973
C	2.941352	3.886795	2.973753
N	2.413679	1.262610	2.182380
Zn	1.907048	-0.610407	1.310328
B	1.700329	5.344028	5.606237
B	-0.570049	4.254605	6.772218
B	-0.814812	5.642259	4.529416
O	-2.058723	5.586171	3.868024
O	-0.540725	6.943770	4.925053
C	-1.584397	7.825997	4.443531
C	-1.868072	8.869796	5.525175
C	-1.052194	8.490705	3.164493
C	-2.774336	6.820637	4.172469
C	-3.620769	6.539109	5.424673
C	-3.671067	7.197158	2.993198
O	-0.757625	3.116802	7.571541
O	-1.119231	5.376698	7.390496
C	-1.672231	3.464788	8.647533
C	-1.448071	5.019624	8.765375
C	-2.675894	5.817627	9.204078
C	-0.230134	5.381399	9.628879
C	-1.301842	2.657618	9.890639
C	-3.085055	3.102704	8.159979
H	-1.267595	3.289116	1.538067
H	-3.195010	4.850554	1.660896
H	-5.520144	4.046940	1.086046
H	-5.861450	1.655795	0.388162
H	-3.902395	0.100488	0.234944
H	-1.586617	-0.639527	6.289249
H	-4.014500	-0.233692	5.706501
H	-4.616696	1.908524	4.462317
H	-2.768670	3.553549	3.932176
H	-0.401255	5.152361	10.700102
H	-0.036800	6.467911	9.533153
H	0.674093	4.852315	9.268921
H	-2.431989	6.899227	9.217726
H	-3.531560	5.672634	8.518155
H	-2.988773	5.521709	10.227049
H	-3.851976	3.260257	8.945331
H	-3.097223	2.035395	7.862245
H	-3.355788	3.702282	7.266895
H	-0.228805	2.769746	10.134948

H	-1.499846	1.580804	9.712830
H	-1.905235	2.979108	10.765016
H	0.461955	1.540298	2.820098
H	4.396029	1.108807	1.569170
H	4.948795	3.510990	2.195136
H	3.149982	4.914062	3.307231
H	-0.993681	9.542725	5.635072
H	-2.749919	9.489317	5.259817
H	-2.044229	8.383473	6.502917
H	-1.753581	9.251533	2.765483
H	-0.089119	8.986460	3.398649
H	-0.857701	7.732625	2.379359
H	-4.252679	7.406466	5.703291
H	-2.964651	6.272716	6.276053
H	-4.286119	5.676067	5.216361
H	-4.138565	8.188808	3.165150
H	-4.486385	6.453324	2.882493
H	-3.107497	7.236152	2.041848
H	-1.339068	2.603365	-1.363507
H	0.926160	2.539323	-2.864888
H	4.865678	2.568363	-2.069254
H	5.620633	3.694366	-4.179527
H	4.529818	3.120222	-6.369267
H	2.683426	1.416862	-6.430481
H	1.939942	0.296211	-4.313841
H	5.646625	-0.038196	-2.483877
H	6.116543	-4.576740	1.951320
H	8.479266	-5.158238	2.544503
H	10.168175	-3.336706	2.923352
H	9.476158	-0.931393	2.683629
H	7.103674	-0.363664	2.103740
H	5.177020	-3.674392	4.122825
H	2.726696	-4.221881	5.127746
H	-1.635003	-4.175894	7.622719
H	-1.234603	-6.616669	7.168625
H	0.005463	-7.302581	5.091566
H	0.834284	-5.564520	3.492244
H	-2.306775	-2.616784	3.959049
H	-3.289130	-0.527747	2.540129
O	1.990690	5.502635	6.965476
O	2.464507	6.233084	4.836749
C	2.748465	6.735591	7.116466
C	3.448984	6.854347	5.707942
C	3.698240	6.587611	8.304148
C	1.731646	7.862980	7.360341
C	3.694238	8.284532	5.227645
C	4.736014	6.017518	5.611006
H	1.029218	7.937084	6.506871
H	1.134372	7.612571	8.257793
H	2.229582	8.839317	7.530086
H	4.368655	7.468697	8.384659

H	3.111953	6.514070	9.242666
H	4.315810	5.673513	8.218301
H	2.742459	8.838329	5.123166
H	4.352744	8.830890	5.934780
H	4.189914	8.269279	4.235583
H	5.066909	5.982454	4.552979
H	4.552184	4.976826	5.946665
H	5.559047	6.451749	6.214334
H	0.490027	4.552461	3.366175
C	5.820613	-1.097959	-0.538719
H	6.846008	-1.484790	-0.521168
H	-0.808611	-2.455849	6.018417
C	3.763455	-0.374313	5.934799
C	4.900568	0.067213	5.220208
C	3.851027	-1.540821	6.726179
C	6.102796	-0.650594	5.301504
H	4.819320	0.967303	4.591251
C	5.060202	-2.247815	6.813337
H	2.965792	-1.884077	7.284620
C	6.187474	-1.806463	6.099114
H	6.981953	-0.316133	4.731625
H	5.118954	-3.155071	7.435038
H	7.133399	-2.368170	6.150622

TS$_{AB, ZS}$

Zero-point correction= 1.539715 (Hartree/Particle)
Thermal correction to Energy= 1.671358
Thermal correction to Enthalpy= 1.672477
Thermal correction to Gibbs Free Energy= 1.367664

solvent: =-5747.72383304

Zn	3.153878	5.959659	0.482717
O	3.151765	4.816283	2.128976
N	3.493273	7.767950	-0.582846
N	2.704382	7.562095	1.905812
N	1.174041	5.346547	-0.034882
C	3.353333	7.740760	-1.891174
H	3.151674	8.695096	-2.424892
C	3.529840	8.951576	0.151539
C	4.018996	10.192006	-0.314533
H	4.408072	10.271125	-1.340627
C	4.018984	11.316072	0.525833
C	3.540893	11.216253	1.848498
H	3.568400	12.094929	2.513437
C	3.079705	9.984439	2.341309
H	2.759726	9.890202	3.390583
C	3.071811	8.841284	1.509077
C	1.797329	7.331546	2.827710
H	1.208954	8.187585	3.234179

C	1.431181	6.038640	3.316284
C	0.283930	5.991525	4.160568
H	-0.216924	6.948127	4.375245
C	-0.200284	4.798099	4.674970
C	0.565517	3.625439	4.394112
H	0.214714	2.681120	4.833149
C	1.726582	3.593369	3.618734
C	2.156946	4.822328	2.967212
C	0.734460	4.080827	0.140979
H	1.447056	3.283479	-0.105320
C	-0.544179	3.819146	0.648511
H	-0.878161	2.778323	0.776773
C	-1.375370	4.886861	1.021823
H	-2.376781	4.713096	1.439543
C	-0.927374	6.217254	0.843168
C	0.353343	6.369236	0.268232
H	0.773900	7.361862	0.074117
C	3.391366	6.563979	-2.707675
C	3.822200	5.258465	-2.215926
C	3.001350	6.734882	-4.066235
C	3.897156	4.183351	-3.188044
C	3.006722	5.686800	-4.980049
H	2.675210	7.742884	-4.372193
C	3.478838	4.430645	-4.501270
H	3.515302	3.595234	-5.211095
O	4.151246	5.052766	-0.972097
H	4.414871	12.272976	0.151103
C	-1.493172	4.707388	5.504551
C	-2.008818	6.108669	5.892402
C	-2.577513	3.997453	4.653350
C	-1.245387	3.906511	6.807136
H	-1.255043	6.673349	6.480260
H	-2.267723	6.706594	4.994382
H	-2.926276	6.023323	6.510202
H	-2.233424	2.998061	4.315127
H	-3.509187	3.852440	5.243182
H	-2.826563	4.594516	3.751849
H	-2.170392	3.868498	7.421348
H	-0.944673	2.858922	6.602114
H	-0.443643	4.375582	7.414835
C	2.511122	5.913591	-6.424282
C	1.023271	6.348593	-6.384315
C	3.353104	7.029497	-7.093450
C	2.621166	4.639326	-7.287434
H	0.397200	5.570494	-5.900724
H	0.885925	7.286141	-5.806618
H	0.631984	6.522237	-7.410210
H	4.425820	6.747100	-7.129037
H	3.007647	7.215052	-8.133509
H	3.278596	7.987963	-6.538752
H	2.255526	4.844547	-8.315294

H	3.670328	4.286754	-7.370424
H	2.010284	3.808847	-6.876352
C	4.419378	2.795573	-2.756263
C	5.875167	2.934772	-2.236675
C	3.516782	2.221052	-1.634241
C	4.426676	1.783908	-3.922414
H	6.539684	3.321276	-3.038056
H	5.921274	3.627540	-1.375471
H	6.264609	1.944665	-1.915070
H	2.473082	2.100973	-1.994354
H	3.885046	1.225038	-1.307826
H	3.517491	2.896221	-0.759141
H	4.817024	0.809091	-3.562716
H	3.408180	1.602892	-4.326705
H	5.078873	2.113626	-4.758590
C	2.569148	2.306256	3.476562
C	2.655552	1.857101	1.997269
C	3.997988	2.592728	4.012883
C	1.981779	1.134719	4.292850
H	1.647387	1.615552	1.600489
H	3.102699	2.653047	1.375475
H	3.284628	0.945822	1.906958
H	3.961884	2.895140	5.080754
H	4.626792	1.679753	3.934651
H	4.481660	3.401628	3.433078
H	2.633160	0.243203	4.180014
H	1.921746	1.367716	5.376916
H	0.967639	0.847858	3.942240
C	1.247760	10.242805	-3.267339
C	2.800165	12.581558	-3.432829
C	1.883418	10.580945	-4.471370
C	1.393385	11.069672	-2.125707
C	2.168239	12.247070	-2.225594
C	2.666122	11.747152	-4.558624
C	0.752608	10.662830	-0.868791
C	0.608285	11.309181	0.360187
H	0.944905	12.280946	0.724341
N	0.135429	9.437647	-0.783653
N	-0.390519	9.304527	0.392889
N	-0.102914	10.429436	1.138703
C	-0.581011	10.495549	2.453610
C	-1.671970	10.542844	4.974058
C	-0.162924	11.500884	3.343278
C	-2.016501	9.566405	4.036330
C	-0.717965	11.520253	4.626834
N	-1.466154	9.520533	2.797928
Ir	-1.954281	8.044667	1.258716
B	-2.612044	7.326938	-0.605337
B	-3.381441	9.517359	0.610510
B	-3.660933	7.243909	2.081964
O	-4.551612	8.016416	2.855990

O	-4.151328	5.944791	1.955217
C	-5.581051	5.991319	2.218395
C	-6.261996	6.314798	0.876985
C	-6.034134	4.632370	2.748803
C	-5.678027	7.178741	3.255204
C	-6.958999	8.006544	3.158036
C	-5.431984	6.731466	4.700853
O	-3.150121	10.854614	0.949358
O	-4.615296	9.373313	-0.017037
C	-4.428301	11.551838	0.882458
C	-5.196469	10.702916	-0.203131
C	-6.706199	10.606093	0.009613
C	-4.882498	11.150620	-1.637266
C	-4.176223	13.011353	0.508474
C	-5.051808	11.440883	2.284013
H	0.592617	12.234358	3.031155
H	-0.410030	12.290726	5.349682
H	-2.144876	10.529708	5.966384
H	-2.777768	8.798824	4.235817
H	-5.310623	12.149924	-1.854589
H	-5.320628	10.426598	-2.350031
H	-3.788251	11.179328	-1.810167
H	-7.156612	9.967969	-0.777139
H	-6.947520	10.158223	0.991170
H	-7.175543	11.610079	-0.047127
H	-6.012050	11.990506	2.358098
H	-4.343206	11.872992	3.019984
H	-5.207984	10.377036	2.554482
H	-3.559984	13.095687	-0.406500
H	-3.635561	13.519973	1.332485
H	-5.134387	13.546948	0.345160
H	-5.935493	5.565685	0.130094
H	-7.367856	6.290406	0.960184
H	-5.937757	7.306717	0.501270
H	-7.100274	4.666770	3.054904
H	-5.928951	3.868998	1.951851
H	-5.425105	4.307174	3.612963
H	-7.850341	7.374548	3.351670
H	-7.060458	8.463411	2.156609
H	-6.943964	8.822032	3.909652
H	-6.294905	6.168960	5.111128
H	-5.265013	7.625556	5.335717
H	-4.532472	6.092750	4.761451
H	2.296814	12.893646	-1.344210
H	3.408466	13.497686	-3.494958
H	3.168562	12.008052	-5.503253
H	1.766499	9.926569	-5.350128
O	-2.109349	7.903958	-1.765754
O	-3.418239	6.233459	-0.888131
C	-2.726224	7.253126	-2.908901
C	-3.211036	5.881777	-2.286404

C	-1.673966	7.130711	-4.012731
C	-3.892306	8.143478	-3.357190
C	-4.527724	5.343985	-2.847203
C	-2.123444	4.797901	-2.332013
H	-4.632376	8.240874	-2.538521
H	-3.502118	9.155219	-3.585044
H	-4.392794	7.746828	-4.263626
H	-2.056051	6.536486	-4.868288
H	-1.409958	8.140107	-4.389472
H	-0.746052	6.659488	-3.637851
H	-5.361864	6.046638	-2.662086
H	-4.447470	5.160594	-3.938746
H	-4.777433	4.382921	-2.353474
H	-2.430365	3.950307	-1.688643
H	-1.159699	5.179536	-1.941041
H	-1.964316	4.421899	-3.362716
H	0.637149	9.330458	-3.185969
H	-1.188814	6.988137	2.282532

L + pyridine

Zero-point correction= 0.773231 (Hartree/Particle)
Thermal correction to Energy= 0.825326
Thermal correction to Enthalpy= 0.826270
Thermal correction to Gibbs Free Energy= 0.685560

solvent: -4429.62097423

N	2.313150	-0.060328	-0.634851
C	2.588903	0.523972	-1.850969
C	3.499067	-0.569204	-0.151877
C	3.997760	0.335021	-2.178360
C	3.693223	-1.160832	1.124954
C	2.681073	-1.367688	2.099927
N	1.341739	-1.100006	1.929574
C	0.711646	-1.420584	3.113232
C	1.690508	-1.899950	4.079356
C	2.908788	-1.895471	3.440537
C	-0.683044	-1.335278	3.344369
C	-1.646086	-0.968654	2.371627
N	-1.376783	-0.337186	1.175627
C	-2.592801	0.006289	0.620220
C	-3.081434	-1.085397	2.557100
C	-3.672255	-0.485460	1.465146
C	-2.768076	0.789909	-0.552697
C	-1.718823	1.199005	-1.419669
N	-0.392723	0.835782	-1.302458
C	0.276165	1.393028	-2.373678
C	-1.888372	2.063249	-2.579706
C	-0.650999	2.183097	-3.171438
C	1.661290	1.236333	-2.657797

C	2.236810	2.118880	-6.333733
C	3.131674	2.943725	-3.803799
C	1.746249	1.491823	-5.176374
C	3.176038	3.160671	-6.230033
C	3.622425	3.570849	-4.960923
C	2.186215	1.895568	-3.894526
C	-6.142825	2.554591	-0.274313
C	-4.799565	0.858290	-2.066133
C	-6.773633	2.155950	-1.466434
C	-4.845040	2.106129	0.020998
C	-4.153755	1.252471	-0.871139
C	-6.098018	1.305825	-2.360642
C	-1.149793	-1.537341	4.756447
C	-1.761087	-1.903866	7.495441
C	-1.290814	-2.831054	5.298091
C	-1.347348	-0.403039	5.602114
C	-1.630342	-0.608949	6.971343
C	-1.604581	-3.019609	6.654711
C	5.817465	-0.850072	2.464264
C	7.015767	-3.049702	1.194926
C	5.089530	-1.566763	1.487499
C	7.126789	-1.229843	2.803885
C	7.730170	-2.330693	2.169859
C	5.705412	-2.671466	0.857077
C	-1.217413	0.982460	5.099564
C	-1.743433	1.570820	3.953395
H	-2.425384	1.200555	3.186545
N	-0.505409	1.960874	5.775083
N	-0.548318	3.084965	5.115239
N	-1.307586	2.865921	3.989399
C	-1.438784	3.825863	2.940195
C	-1.710807	5.678839	0.945762
C	-1.664577	3.361522	1.625088
C	-1.447430	6.016677	2.286745
C	-1.820090	4.317702	0.613879
N	-1.325136	5.114509	3.274940
C	1.747015	4.283702	1.123786
C	1.437604	2.030493	2.679049
C	1.309405	3.061202	0.597188
C	2.028300	4.363353	2.497892
C	1.882450	3.212574	3.288842
N	1.147624	1.961981	1.363150
Zn	0.528275	0.114925	0.440057
H	-4.344866	2.426523	0.948270
H	-6.662240	3.224292	0.429522
H	-7.791860	2.506554	-1.698187
H	-6.588656	0.982814	-3.292698
H	-4.271316	0.186048	-2.760283
H	-1.664374	2.289685	1.385729
H	-1.994971	3.983823	-0.421548
H	-1.817671	6.465091	0.183036

H	-1.337464	7.075515	2.583855
H	1.281130	1.106068	3.257894
H	1.055819	2.944468	-0.468769
H	1.844320	5.159914	0.465968
H	2.348671	5.313745	2.951913
H	-2.831665	2.519126	-2.901913
H	-0.389949	2.758225	-4.067003
H	3.476606	3.264338	-2.807956
H	4.355674	4.388221	-4.870648
H	3.560990	3.651654	-7.137882
H	1.887515	1.786920	-7.324498
H	1.016430	0.670808	-5.254956
H	4.487273	0.670816	-3.099503
H	5.140893	-3.235434	0.097911
H	7.479921	-3.915773	0.696597
H	8.757010	-2.628181	2.435492
H	7.681291	-0.658554	3.565485
H	5.343632	0.015521	2.953588
H	3.876208	-2.222107	3.838594
H	1.465312	-2.222140	5.102524
H	-1.747529	0.272935	7.619735
H	-1.989346	-2.042045	8.564093
H	-1.713251	-4.039211	7.057110
H	-1.129253	-3.697295	4.637496
H	-3.567198	-1.565680	3.415073
H	-4.741623	-0.393269	1.243330
H	2.066986	3.230673	4.371199
C	4.564005	-0.334055	-1.120392
H	5.610019	-0.637935	-1.000207

L + pyridine deprotonated in *para* position

Zero-point correction= 0.757532 (Hartree/Particle)
Thermal correction to Energy= 0.809878
Thermal correction to Enthalpy= 0.810822
Thermal correction to Gibbs Free Energy= 0.669346

solvent: -4429.03548030

N	2.337344	-0.144521	-0.669954
C	2.605747	0.378333	-1.915785
C	3.525395	-0.623682	-0.167391
C	4.006628	0.167841	-2.245195
C	3.722705	-1.135524	1.149893
C	2.711995	-1.299153	2.131556
N	1.372575	-1.038916	1.953384
C	0.730785	-1.388489	3.123649
C	1.708134	-1.854933	4.100264
C	2.933065	-1.823330	3.477989
C	-0.665441	-1.353017	3.329628
C	-1.625753	-0.986486	2.345064

N	-1.348385	-0.314562	1.171995
C	-2.561700	0.034938	0.614959
C	-3.056426	-1.125467	2.509172
C	-3.643496	-0.484375	1.430861
C	-2.728570	0.834259	-0.557140
C	-1.683991	1.184878	-1.449712
N	-0.379045	0.742962	-1.366292
C	0.299031	1.278284	-2.440537
C	-1.829813	2.066649	-2.603057
C	-0.602089	2.119064	-3.220973
C	1.676227	1.082948	-2.733182
C	2.235851	1.951254	-6.418239
C	3.179705	2.747170	-3.896684
C	1.743077	1.334913	-5.255441
C	3.204137	2.967147	-6.324447
C	3.674081	3.362660	-5.058402
C	2.206530	1.722589	-3.976471
C	-6.035313	2.695144	-0.136945
C	-4.778480	1.055110	-2.037049
C	-6.697530	2.387583	-1.340072
C	-4.751161	2.186001	0.113679
C	-4.098145	1.358954	-0.833199
C	-6.063157	1.565158	-2.288776
C	-1.156019	-1.614859	4.723306
C	-1.817323	-2.118691	7.432972
C	-1.250967	-2.933469	5.215520
C	-1.430105	-0.524715	5.606791
C	-1.736999	-0.800327	6.959770
C	-1.586525	-3.192567	6.555415
C	5.815548	-0.759641	2.519959
C	7.091796	-2.943638	1.302407
C	5.120400	-1.504013	1.538109
C	7.125854	-1.104935	2.891505
C	7.770040	-2.197755	2.283692
C	5.779655	-2.600632	0.935356
C	-1.349839	0.885535	5.173227
C	-1.788842	1.493781	4.001318
H	-2.349121	1.121654	3.144073
N	-0.789208	1.875378	5.965052
N	-0.837486	3.024596	5.348778
N	-1.455171	2.809518	4.140776
C	-1.566594	3.808994	3.123765
C	-1.745431	5.730193	1.191726
C	-1.613816	3.391461	1.775110
C	-1.682378	6.018698	2.566417
C	-1.713438	4.383042	0.793272
N	-1.614703	5.080883	3.525599
C	1.727961	4.365437	0.823316
C	1.307513	2.246271	2.504990
C	1.361990	3.096924	0.346169
C	1.871427	4.625219	2.206149

C	1.652048	3.501522	3.037943
N	1.144764	2.048855	1.175163
Zn	0.571916	0.175930	0.435547
H	-4.224050	2.440809	1.046134
H	-6.518131	3.345769	0.610170
H	-7.705260	2.787829	-1.537443
H	-6.576432	1.311509	-3.230856
H	-4.284341	0.401800	-2.773167
H	-1.506615	2.335973	1.490970
H	-1.733478	4.088771	-0.268045
H	-1.797442	6.544459	0.452905
H	-1.692138	7.067268	2.916188
H	1.130004	1.359679	3.140911
H	1.221358	2.891039	-0.731158
H	1.883089	5.159620	0.064419
H	-2.748922	2.587559	-2.895963
H	-0.325893	2.692035	-4.113758
H	3.535880	3.060050	-2.902568
H	4.426486	4.163556	-4.973389
H	3.590997	3.450978	-7.236038
H	1.864388	1.631690	-7.405724
H	0.988048	0.536028	-5.324608
H	4.492250	0.456459	-3.184539
H	5.242692	-3.186654	0.172815
H	7.586039	-3.804737	0.823298
H	8.798693	-2.467262	2.573481
H	7.650218	-0.509825	3.656841
H	5.311512	0.101861	2.985467
H	3.900355	-2.136902	3.887457
H	1.475736	-2.187442	5.118807
H	-1.907211	0.051665	7.636020
H	-2.063555	-2.307914	8.490484
H	-1.653980	-4.232027	6.915564
H	-1.031125	-3.762883	4.524862
H	-3.547261	-1.633115	3.348532
H	-4.711670	-0.387229	1.204085
H	1.708686	3.576697	4.140749
C	4.582060	-0.444394	-1.152087
H	5.631315	-0.733443	-1.022528

L + pyridine deprotonated in ortho position

Zero-point correction= 0.757043 (Hartree/Particle)
Thermal correction to Energy= 0.809516
Thermal correction to Enthalpy= 0.810460
Thermal correction to Gibbs Free Energy= 0.668051

solvent: -4429.03146175

N	2.295744	-0.117390	-0.681791
C	2.563290	0.452182	-1.902170

C	3.485182	-0.609458	-0.192369
C	3.973415	0.271304	-2.230001
C	3.683308	-1.172546	1.098167
C	2.676862	-1.375259	2.079208
N	1.333217	-1.139605	1.900307
C	0.710339	-1.437738	3.092781
C	1.697471	-1.876787	4.072775
C	2.915776	-1.864590	3.433660
C	-0.683804	-1.355319	3.325383
C	-1.649242	-0.987473	2.351263
N	-1.377898	-0.337755	1.164509
C	-2.592051	0.029051	0.623381
C	-3.082604	-1.096261	2.541291
C	-3.672121	-0.462070	1.463301
C	-2.764077	0.822918	-0.548640
C	-1.723946	1.193612	-1.438671
N	-0.407482	0.803025	-1.336125
C	0.254590	1.318842	-2.427487
C	-1.894106	2.048301	-2.610998
C	-0.667239	2.120912	-3.226137
C	1.623464	1.126387	-2.733524
C	2.218696	2.043961	-6.403234
C	3.069859	2.858717	-3.853753
C	1.728852	1.395134	-5.256811
C	3.139861	3.100033	-6.279434
C	3.561999	3.505185	-4.999250
C	2.148288	1.789509	-3.964974
C	-6.064714	2.698184	-0.149140
C	-4.837086	0.986832	-2.005341
C	-6.746055	2.343822	-1.328304
C	-4.775629	2.199830	0.099474
C	-4.139423	1.336222	-0.824955
C	-6.126373	1.486451	-2.255572
C	-1.149301	-1.559686	4.737808
C	-1.758154	-1.968845	7.476923
C	-1.252496	-2.860216	5.275081
C	-1.385383	-0.437238	5.593501
C	-1.667396	-0.667745	6.960857
C	-1.562994	-3.073146	6.628739
C	5.788900	-0.780562	2.444859
C	7.063624	-2.962111	1.221099
C	5.087344	-1.533850	1.475209
C	7.105877	-1.116029	2.801670
C	7.748980	-2.207876	2.190635
C	5.744555	-2.628773	0.868890
C	-1.296485	0.960262	5.121092
C	-1.732473	1.537792	3.932613
H	-2.291743	1.145574	3.084689
N	-0.736702	1.971143	5.886503
N	-0.781434	3.103947	5.236784
N	-1.395041	2.855541	4.033705

C	-1.477604	3.812151	2.971728
C	-1.604058	5.637982	0.943942
C	-1.520363	3.333223	1.646448
C	-1.551633	5.993029	2.304670
C	-1.580584	4.272306	0.609388
N	-1.497760	5.106840	3.313017
C	1.776673	4.214958	1.135045
C	1.429529	2.006589	2.717353
C	1.337485	2.999554	0.536946
C	2.001944	4.330016	2.514760
C	1.828477	3.194622	3.332714
N	1.182192	1.936112	1.382939
Zn	0.551642	0.203005	0.455872
H	-4.231311	2.490057	1.011707
H	-6.536336	3.376742	0.580152
H	-7.757449	2.735603	-1.524317
H	-6.654206	1.198161	-3.179497
H	-4.351879	0.308847	-2.724920
H	-1.425105	2.265110	1.415609
H	-1.568980	3.921384	-0.433793
H	-1.638250	6.416684	0.166381
H	-1.555263	7.059157	2.600269
H	1.267741	1.081549	3.296917
H	1.907077	5.108221	0.495867
H	2.297163	5.294498	2.967170
H	-2.827730	2.535076	-2.916453
H	-0.403498	2.678811	-4.132088
H	3.376408	3.181138	-2.846269
H	4.274045	4.339947	-4.891434
H	3.526276	3.607760	-7.178311
H	1.883006	1.718169	-7.401625
H	1.011051	0.564636	-5.348747
H	4.459611	0.600776	-3.155708
H	5.202083	-3.218930	0.113533
H	7.557951	-3.821188	0.738467
H	8.782866	-2.469749	2.468736
H	7.636398	-0.514681	3.557927
H	5.283720	0.079311	2.912117
H	3.889338	-2.159022	3.842924
H	1.479165	-2.170139	5.106370
H	-1.810667	0.207405	7.613348
H	-1.985086	-2.120898	8.544760
H	-1.639247	-4.099906	7.022101
H	-1.060179	-3.714009	4.606238
H	-3.569585	-1.580457	3.396644
H	-4.741808	-0.350469	1.249815
H	1.958836	3.234919	4.423855
C	4.548672	-0.378791	-1.162749
H	5.599812	-0.663864	-1.037982

L + pyridine deprotonated in *meta* position

Zero-point correction= 0.757603 (Hartree/Particle)
Thermal correction to Energy= 0.809940
Thermal correction to Enthalpy= 0.810884
Thermal correction to Gibbs Free Energy= 0.669350

solvent: -4429.03718674

N	2.327805	-0.163118	-0.693478
C	2.592826	0.378678	-1.930866
C	3.517295	-0.654979	-0.204847
C	3.993438	0.175933	-2.267655
C	3.720632	-1.188904	1.100266
C	2.718389	-1.343776	2.093418
N	1.378966	-1.078233	1.925108
C	0.754478	-1.378120	3.118380
C	1.739437	-1.835952	4.090563
C	2.954196	-1.841120	3.447149
C	-0.639998	-1.312669	3.346392
C	-1.607956	-0.949687	2.373344
N	-1.340817	-0.293880	1.187470
C	-2.555386	0.052779	0.638835
C	-3.038742	-1.081309	2.554249
C	-3.632443	-0.454253	1.473707
C	-2.734623	0.831814	-0.544799
C	-1.694774	1.169626	-1.451621
N	-0.388430	0.742563	-1.364367
C	0.282901	1.268390	-2.448593
C	-1.852928	2.030369	-2.619750
C	-0.626650	2.086672	-3.240404
C	1.659201	1.082692	-2.741816
C	2.273140	1.959684	-6.416443
C	3.108233	2.811000	-3.872931
C	1.777794	1.325652	-5.264057
C	3.189351	3.020640	-6.300727
C	3.603957	3.444442	-5.024551
C	2.188967	1.740577	-3.976624
C	-6.039865	2.693023	-0.117063
C	-4.797096	1.035572	-2.011033
C	-6.712462	2.372074	-1.310861
C	-4.752583	2.188904	0.127234
C	-4.106059	1.352009	-0.816037
C	-6.084435	1.541167	-2.256741
C	-1.117812	-1.567754	4.746207
C	-1.767660	-2.069012	7.459113
C	-1.190741	-2.885393	5.245358
C	-1.407182	-0.477661	5.624496
C	-1.707842	-0.752135	6.979320
C	-1.521116	-3.143428	6.586482
C	5.841647	-0.842039	2.437019
C	7.065735	-3.043365	1.197627

C	5.118879	-1.576971	1.468334
C	7.153419	-1.205375	2.785431
C	7.771390	-2.307228	2.166734
C	5.752080	-2.682179	0.853902
C	-1.344785	0.932819	5.187623
C	-1.808080	1.538656	4.023372
H	-2.382837	1.163978	3.176508
N	-0.768743	1.923600	5.966139
N	-0.828634	3.071576	5.346683
N	-1.470844	2.854185	4.152427
C	-1.567235	3.843627	3.122559
C	-1.658508	5.743628	1.163927
C	-1.598981	3.409850	1.778444
C	-1.638288	6.048039	2.535839
C	-1.641900	4.391257	0.782474
N	-1.601711	5.120680	3.509319
C	1.667852	4.397229	0.846140
C	1.286612	2.182674	2.564613
C	1.367782	3.093168	0.413050
C	1.766635	4.531133	2.249511
C	1.598222	3.431490	3.119869
N	1.158623	2.014422	1.229944
Zn	0.581882	0.172784	0.433429
H	-4.216992	2.456394	1.051394
H	-6.516794	3.351234	0.627277
H	-7.722632	2.768488	-1.503431
H	-6.605389	1.277042	-3.191748
H	-4.308975	0.375068	-2.744789
H	-1.505071	2.349151	1.509619
H	-1.625870	4.087374	-0.275606
H	-1.661092	6.547529	0.412789
H	-1.647187	7.101083	2.872856
H	1.105300	1.292518	3.190987
H	1.255107	2.848514	-0.661185
H	1.965677	5.520895	2.711162
H	-2.777622	2.537632	-2.918512
H	-0.356426	2.647944	-4.142327
H	3.416201	3.147924	-2.870559
H	4.312773	4.282267	-4.923163
H	3.577812	3.518758	-7.203990
H	1.944121	1.619789	-7.412201
H	1.061802	0.492906	-5.348516
H	4.474318	0.485360	-3.202992
H	5.194135	-3.259164	0.099597
H	7.539843	-3.910479	0.709145
H	8.801271	-2.590821	2.438130
H	7.699890	-0.617361	3.540760
H	5.357996	0.026811	2.910396
H	3.923977	-2.155293	3.850134
H	1.520030	-2.134570	5.122406
H	-1.890591	0.100219	7.651802

H	-2.010339	-2.256794	8.517695
H	-1.572729	-4.182138	6.951249
H	-0.959224	-3.714666	4.558417
H	-3.523510	-1.574164	3.405860
H	-4.702326	-0.360377	1.253419
H	1.653913	3.535513	4.216168
C	4.571807	-0.460212	-1.191153
H	5.621298	-0.751749	-1.069259

Pyridine deprotonated in *para* position

Zero-point correction= 0.071030 (Hartree/Particle)
Thermal correction to Energy= 0.075515
Thermal correction to Enthalpy= 0.076459
Thermal correction to Gibbs Free Energy= 0.043473

solvent: -247.795857117

C	0.639059	-1.542219	0.000167
C	2.049577	-1.516061	0.000204
C	2.832524	-0.317474	0.000093
C	1.955667	0.814256	-0.000063
C	0.547622	0.726878	-0.000105
N	-0.139425	-0.437221	0.000013
H	2.370653	1.853438	-0.000156
H	-0.077100	1.653419	-0.000248
H	0.090888	-2.516106	0.000281
H	2.546785	-2.518571	0.000326

Pyridine deprotonated in *meta* position

Zero-point correction= 0.071028 (Hartree/Particle)
Thermal correction to Energy= 0.075549
Thermal correction to Enthalpy= 0.076494
Thermal correction to Gibbs Free Energy= 0.043447

solvent: -247.793122609

C	0.677514	-1.544615	0.000059
C	2.104108	-1.661605	0.000062
C	2.688089	-0.358068	-0.000125
C	1.941183	0.843386	-0.000190
C	0.536955	0.748848	-0.000071
N	-0.113881	-0.430965	0.000051
H	3.801588	-0.241932	-0.000261
H	2.427043	1.842979	-0.000312
H	-0.090077	1.668281	-0.000137
H	0.062081	-2.479903	0.000485

Pyridine deprotonated in ortho position

Zero-point correction= 0.070188 (Hartree/Particle)

Thermal correction to Energy= 0.074881
Thermal correction to Enthalpy= 0.075825
Thermal correction to Gibbs Free Energy= 0.042462

solvent: -247.782396473

C	0.564544	-1.671245	0.000241
C	2.005736	-1.545140	0.000154
C	2.708366	-0.326436	-0.000084
C	1.962103	0.869140	-0.000203
C	0.558846	0.732747	-0.000081
N	-0.110472	-0.435916	0.000099
H	3.820660	-0.289964	-0.000189
H	2.612068	-2.479593	0.000276
H	2.443508	1.865501	-0.000393
H	-0.062551	1.662891	-0.000163

TS AB - Bpin

Zero-point correction= 1.466399 (Hartree/Particle)
Thermal correction to Energy= 1.597998
Thermal correction to Enthalpy= 1.599116
Thermal correction to Gibbs Free Energy= 1.290514

solvent: -6179.33897100

N	3.144851	-0.478606	-0.615910
C	3.605316	0.103370	-1.777921
C	4.209719	-1.126116	-0.031648
C	5.002854	-0.257837	-1.979529
C	4.189953	-1.794351	1.223110
C	3.037071	-2.027241	2.020023
N	1.754225	-1.637098	1.703590
C	0.933000	-2.069326	2.721288
C	1.728901	-2.750980	3.733594
C	3.035528	-2.717179	3.302858
C	-0.469834	-1.850837	2.796775
C	-1.214394	-1.053209	1.888132
N	-0.665204	-0.255690	0.908232
C	-1.656697	0.587731	0.470043
C	-2.643318	-0.793078	1.980355
C	-2.910793	0.256778	1.130719
C	-1.495302	1.655814	-0.456043
C	-0.397286	1.802390	-1.345037
N	0.689743	0.956568	-1.396686
C	1.549431	1.464068	-2.341564
C	-0.230693	2.879721	-2.313459
C	0.964651	2.652662	-2.954447
C	2.879599	1.020960	-2.585000
C	3.923393	2.121240	-6.091567
C	4.690467	2.577069	-3.422491
C	3.240434	1.481015	-5.043555
C	4.994217	2.987592	-5.809098
C	5.371832	3.214817	-4.472945
C	3.619539	1.693597	-3.698998
C	-3.628058	4.412662	1.053243
C	-3.338347	3.137321	-1.437555
C	-4.438220	4.799902	-0.030473
C	-2.678628	3.392211	0.891669
C	-2.519012	2.738359	-0.357867
C	-4.289664	4.159702	-1.274323
C	-1.201445	-2.429026	3.966525
C	-2.621133	-3.619351	6.127647
C	-1.240833	-3.831774	4.138756
C	-1.888067	-1.613397	4.918743
C	-2.591344	-2.224981	5.981933
C	-1.937790	-4.426373	5.201639
C	6.473867	-1.290733	2.155233

C	7.044896	-4.022204	2.466664
C	5.506143	-2.248455	1.771491
C	7.709306	-1.693274	2.688935
C	7.998693	-3.060791	2.846737
C	5.808627	-3.619356	1.933723
C	-1.912053	-0.140845	4.814776
C	-2.991779	0.717924	5.012345
H	-4.035019	0.524516	5.271583
N	-0.817769	0.613198	4.456070
N	-1.161334	1.865244	4.428243
N	-2.501445	1.972642	4.749086
C	-3.098891	3.239376	4.741549
C	-4.133213	5.785893	4.718300
C	-4.491102	3.399626	4.866343
C	-2.763594	5.541270	4.586895
C	-5.014122	4.695955	4.855296
N	-2.246028	4.288046	4.590726
Ir	-0.112843	3.785360	4.469754
C	3.116234	3.703944	1.339668
C	1.008097	2.136686	2.171781
C	2.880831	2.418279	0.817319
C	1.171895	3.382984	2.806964
C	2.246285	4.176729	2.345812
N	1.846461	1.666309	1.228779
Zn	1.326138	-0.138268	0.304866
B	1.567682	2.808545	5.321740
B	-0.245005	3.924209	6.605834
B	0.698891	5.641366	4.836652
O	-0.081750	6.760208	5.206212
O	2.034367	6.008830	4.738980
C	2.206285	7.355138	5.248359
C	2.657196	7.220175	6.710518
C	3.278628	8.055060	4.410186
C	0.748442	7.951870	5.100442
C	0.338373	8.931633	6.199339
C	0.488041	8.552022	3.709388
O	-1.454162	3.576927	7.220894
O	0.600224	4.565220	7.508744
C	-1.557918	4.369778	8.438242
C	-0.041531	4.563867	8.820965
C	0.276396	5.884181	9.522610
C	0.526420	3.378612	9.610537
C	-2.396235	3.605720	9.460639
C	-2.240458	5.690033	8.039309
H	-2.029661	3.101018	1.734219
H	-3.729711	4.911084	2.030192
H	-5.185008	5.600517	0.093523
H	-4.926802	4.452490	-2.124360
H	-3.235751	2.622687	-2.405776
H	-5.145683	2.522626	4.957460
H	-6.099335	4.852966	4.948218

H	-4.499277	6.822447	4.712101
H	-2.015145	6.345613	4.514957
H	0.119469	3.340752	10.641289
H	1.626902	3.481327	9.672874
H	0.302241	2.425208	9.093103
H	1.359767	5.939937	9.750117
H	0.017307	6.752446	8.888132
H	-0.282015	5.963054	10.478539
H	-2.429456	6.342947	8.915429
H	-3.213231	5.456279	7.560890
H	-1.622444	6.240492	7.299337
H	-2.011764	2.579503	9.611661
H	-3.443652	3.528978	9.104040
H	-2.403315	4.132932	10.437185
H	0.191388	1.451751	2.436359
H	3.528184	1.975241	0.045001
H	2.432487	5.157483	2.803341
H	3.587608	6.618975	6.736285
H	2.858427	8.205641	7.177927
H	1.895400	6.672453	7.296971
H	3.365688	9.125432	4.691175
H	4.258751	7.567736	4.583701
H	3.064698	7.978946	3.327343
H	1.013108	9.812436	6.214875
H	0.363999	8.448884	7.194536
H	-0.695252	9.292374	6.020133
H	1.038679	9.502154	3.556821
H	-0.596433	8.757459	3.600003
H	0.782830	7.838263	2.913808
H	-0.927322	3.710737	-2.472430
H	1.433801	3.259022	-3.737952
H	4.965629	2.777141	-2.373643
H	6.202498	3.901729	-4.246651
H	5.532082	3.489203	-6.629499
H	3.619429	1.939246	-7.134933
H	2.401472	0.800410	-5.258275
H	5.613795	0.029980	-2.842687
H	5.061043	-4.369031	1.630386
H	7.267040	-5.095367	2.581208
H	8.967563	-3.377403	3.264852
H	8.449306	-0.933630	2.988155
H	6.236739	-0.221768	2.036342
H	3.919457	-3.110896	3.817060
H	1.345449	-3.177597	4.667320
H	-3.095013	-1.584475	6.723703
H	-3.167848	-4.073715	6.968740
H	-1.952503	-5.523135	5.302426
H	-0.718836	-4.459072	3.399776
H	-3.348696	-1.336787	2.619268
H	-3.875263	0.739440	0.933129
O	1.385529	1.695368	6.142850

O	2.904204	2.982493	4.999915
C	2.694019	1.124717	6.445089
C	3.591108	1.730726	5.293730
C	2.567326	-0.397813	6.423293
C	3.098749	1.623532	7.837788
C	5.032057	2.053590	5.688007
C	3.567570	0.873063	4.017024
H	3.145729	2.730639	7.856209
H	2.337724	1.298973	8.573609
H	4.079547	1.211021	8.149610
H	3.562225	-0.878288	6.528843
H	1.927037	-0.734225	7.264020
H	2.105712	-0.747837	5.481947
H	5.073720	2.805922	6.498198
H	5.564437	1.137059	6.016558
H	5.568325	2.468898	4.810807
H	3.981278	1.463452	3.176108
H	2.537514	0.575147	3.741169
H	4.169944	-0.050790	4.127035
H	-0.304515	4.306349	2.906452
C	5.380927	-1.009394	-0.890590
H	6.360996	-1.457482	-0.689432
B	4.309025	4.553744	0.821214
O	4.610267	5.817702	1.288804
O	5.181823	4.112408	-0.160129
C	5.640620	6.363673	0.412046
C	6.297626	5.052725	-0.188445
C	4.917616	7.208101	-0.649153
C	6.583352	7.232678	1.243506
C	6.797170	5.186188	-1.626052
C	7.393628	4.462533	0.712903
H	4.294029	7.967740	-0.137023
H	4.248017	6.578179	-1.268740
H	5.632079	7.731088	-1.316258
H	6.974493	6.682728	2.119829
H	6.039219	8.123002	1.618106
H	7.438235	7.582152	0.628538
H	5.970711	5.427413	-2.320182
H	7.250592	4.229646	-1.956104
H	7.570694	5.978158	-1.699237
H	8.318900	5.072748	0.689757
H	7.044651	4.384800	1.762357
H	7.635347	3.441270	0.356881

TS AB - ortho

Zero-point correction= 1.300206 (Hartree/Particle)
Thermal correction to Energy= 1.417382
Thermal correction to Enthalpy= 1.418501
Thermal correction to Gibbs Free Energy= 1.142024

solvent: -5764.35545186

N	3.131692	0.072167	0.306128
C	3.639049	0.641108	-0.847960
C	4.114158	-0.761384	0.800882
C	4.995179	0.166562	-1.076230
C	3.979536	-1.643791	1.904577
C	2.777050	-1.872963	2.619111
N	1.570627	-1.241230	2.393560
C	0.653382	-1.834468	3.236832
C	1.313729	-2.827736	4.069945
C	2.629616	-2.856449	3.684615
C	-0.740156	-1.584842	3.254099
C	-1.393987	-0.629249	2.434870
N	-0.776532	0.263853	1.577844
C	-1.772498	1.041597	1.026003
C	-2.831341	-0.427131	2.396218
C	-3.065213	0.632317	1.548536
C	-1.608704	2.030493	0.020890
C	-0.438083	2.200572	-0.759466
N	0.729084	1.484663	-0.600190
C	1.594870	1.918510	-1.580258
C	-0.300315	3.135937	-1.867700
C	0.954561	2.944162	-2.392068
C	2.949039	1.521606	-1.720371
C	4.127948	2.491950	-5.227674
C	4.817239	2.996907	-2.553833
C	3.397211	1.891585	-4.188755
C	5.204843	3.347631	-4.933233
C	5.547531	3.598094	-3.592195
C	3.731179	2.135731	-2.837018
C	-4.319624	4.603973	0.701355
C	-3.441025	3.026120	-1.453511
C	-4.973240	4.691645	-0.541447
C	-3.230455	3.734048	0.862406
C	-2.770025	2.937617	-0.212466
C	-4.531716	3.897455	-1.615804
C	-1.586576	-2.427741	4.170168
C	-3.238690	-4.069168	5.809397
C	-1.700830	-3.816183	3.937983
C	-2.321232	-1.870781	5.262736
C	-3.142888	-2.691602	6.061293
C	-2.511522	-4.630924	4.745751
C	6.342413	-1.816923	2.792154
C	6.320283	-4.608524	2.507980
C	5.182169	-2.448586	2.288979
C	7.471959	-2.568721	3.155464
C	7.464265	-3.967988	3.017508
C	5.191196	-3.855866	2.145585
C	-2.254876	-0.411525	5.454218
C	-3.278648	0.530982	5.385608

H	-4.365505	0.426311	5.339228
N	-1.050855	0.243482	5.366086
N	-1.279336	1.504631	5.188333
N	-2.641025	1.729223	5.204994
C	-3.136054	3.014289	4.913850
C	-3.955367	5.553313	4.308587
C	-4.440090	3.392344	5.277087
C	-2.675316	5.082509	3.993160
C	-4.856032	4.690463	4.958919
N	-2.265331	3.826908	4.275440
Ir	-0.080684	3.012309	4.283712
C	1.113856	5.056868	0.602026
C	2.350078	4.438113	0.345252
C	0.755784	3.469778	2.425747
C	0.319672	4.572234	1.646811
Zn	1.279217	0.479311	1.209151
B	1.759104	2.198545	4.722605
B	0.000058	3.773185	6.325958
B	0.716388	4.973857	4.521532
O	-0.062682	6.097130	4.875803
O	1.986903	5.368806	4.155655
C	2.188076	6.753362	4.508722
C	2.921014	6.754032	5.859833
C	3.047812	7.404926	3.422957
C	0.704648	7.306499	4.595683
C	0.464156	8.315627	5.719292
C	0.184970	7.868459	3.264031
O	-1.234899	3.760597	6.984632
O	1.012530	4.170539	7.191274
C	-1.098561	4.467606	8.245744
C	0.451514	4.326790	8.533650
C	1.091831	5.551544	9.187071
C	0.801551	3.056474	9.321241
C	-2.009958	3.803825	9.279072
C	-1.546140	5.912871	7.978929
H	-2.708879	3.649537	1.826278
H	-4.655992	5.216211	1.553577
H	-5.828819	5.373281	-0.672561
H	-5.048002	3.949636	-2.587849
H	-3.109010	2.389676	-2.288474
H	-5.095428	2.694578	5.816681
H	-5.868038	5.028360	5.229147
H	-4.234536	6.584922	4.050000
H	-1.927930	5.725336	3.510931
H	0.426238	3.106688	10.363242
H	1.901926	2.938828	9.355567
H	0.382258	2.157477	8.827690
H	2.179915	5.383025	9.313902
H	0.958950	6.459596	8.570075
H	0.649282	5.737034	10.187735
H	-1.509014	6.540391	8.892250

H	-2.590387	5.894118	7.605547
H	-0.923372	6.366072	7.184826
H	-1.821000	2.716147	9.350133
H	-3.069643	3.947513	8.984938
H	-1.867848	4.257813	10.281643
H	3.036829	4.802333	-0.432592
H	0.782255	5.923689	0.007539
H	-0.627235	5.070060	1.900769
H	3.860632	6.178702	5.743765
H	3.167933	7.778672	6.205351
H	2.315812	6.231644	6.625402
H	3.144105	8.497757	3.591286
H	4.062232	6.959219	3.441490
H	2.624733	7.225767	2.416270
H	1.077006	9.226928	5.560884
H	0.718797	7.893168	6.708677
H	-0.603460	8.615394	5.738965
H	0.677148	8.825944	3.000116
H	-0.905629	8.055224	3.348964
H	0.353858	7.147776	2.441751
H	-1.056722	3.858153	-2.193340
H	1.421172	3.478826	-3.226733
H	5.080194	3.192250	-1.502032
H	6.387171	4.269995	-3.352286
H	5.777395	3.818079	-5.748395
H	3.857613	2.285644	-6.275725
H	2.555777	1.218202	-4.415362
H	5.622860	0.431603	-1.934485
H	4.298699	-4.354530	1.736365
H	6.309089	-5.703381	2.384530
H	8.349619	-4.558439	3.302412
H	8.363208	-2.056772	3.552350
H	6.346446	-0.721826	2.897796
H	3.436171	-3.469442	4.100555
H	0.833236	-3.419766	4.856513
H	-3.694443	-2.239000	6.900931
H	-3.876140	-4.703393	6.445175
H	-2.581827	-5.710023	4.536146
H	-1.145738	-4.251774	3.092857
H	-3.569862	-1.043638	2.921533
H	-4.030900	1.054814	1.247812
O	1.962772	0.886267	5.160664
O	2.946199	2.921268	4.737500
C	3.286536	0.858424	5.779039
C	4.026095	1.997153	4.985732
C	3.909973	-0.523835	5.641347
C	3.082870	1.218585	7.259056
C	5.121905	2.723790	5.767309
C	4.548885	1.528192	3.619037
H	2.677343	2.245433	7.344851
H	2.344314	0.517305	7.696797

H	4.025277	1.147697	7.840054
H	4.922162	-0.534617	6.097034
H	3.282825	-1.279245	6.154687
H	3.998105	-0.827135	4.585229
H	4.706091	3.230666	6.658732
H	5.915030	2.016963	6.089828
H	5.587562	3.498495	5.124514
H	4.821445	2.418087	3.018327
H	3.766024	0.976159	3.063169
H	5.443798	0.883248	3.723491
H	-0.375873	1.985879	3.034120
C	5.291142	-0.700911	-0.051834
H	6.207982	-1.283559	0.091597
N	1.816475	2.731898	1.982602
C	2.622784	3.251142	1.033851
H	3.499886	2.635700	0.777781
